# The impact of economic sanctions on health and health systems in low-income and middle-income countries: a systematic review and narrative synthesis

**DOI:** 10.1136/bmjgh-2022-010968

**Published:** 2023-02-09

**Authors:** Matteo Pinna Pintor, Marc Suhrcke, Christoph Hamelmann

**Affiliations:** 1 Living Conditions, Luxembourg Institute of Socio-Economic Research, Esch-sur-Alzette, Luxembourg; 2 University of York Centre for Health Economics, York, UK; 3 EMRO, Cairo, Egypt

**Keywords:** Health economics, Epidemiology, Health systems, Prevention strategies, Systematic review

## Abstract

**Introduction:**

Economic sanctions restrict customary commercial and financial ties between states to induce change in political constitution or conduct of the targeted country. Although the stated goals of sanctions often include humanitarian objectives, prospective procedures for health risk assessment are not regularly incorporated in their implementation. Moreover, past experience suggests that the burden of economic isolation may fall on the civilian population. We present key findings from a WHO-sponsored evidence review on the impact of economic sanctions on health and health systems in low-income and middle-income countries, aiming at comprehensive coverage and explicit consideration of issues of causality and mechanisms.

**Methods:**

Broad searches of PubMed and Google Scholar (1970–2021) were designed to retrieve published and grey English-language literature expected to cut across disciplines, terminology and research methods. Studies providing an impact estimate were rated by a structured assessment based on ROBINS-I risk of bias domains, synthesised via vote counting and contextualised into the broader literature through a thematic synthesis.

**Results:**

Included studies (185) were mostly peer-reviewed, mostly single-country, largely coming from medicine and public health, and chiefly concerned with three important target countries—Iraq, Haiti and Iran. Among studies providing impact estimates (31), most raised multiple risk-of-bias concerns. Excluding those with data integrity issues, a significant proportion (21/27) reported consistently adverse effects of sanctions across examined outcomes, with no apparent association to assessed quality, focus on early episodes or publication period. The thematic synthesis highlights the complexity of sanctions, their multidimensionality and the possible mechanisms of impact.

**Conclusion:**

Future research should draw on qualitative knowledge to collect domain-relevant data, combining it with better estimation techniques and study design. However, only the adoption of a risk assessment framework based on prospective data collection and monitoring can certify claims that civilians are adequately protected.

**WHAT IS ALREADY KNOWN ON THIS TOPIC**

- Economic sanctions restrict customary economic ties between states to pursue foreign policy goals. Low-income and middle-income countries (LMICs) figure prominently among targeted states.
- Lack of prospective risk assessment and experience of key past episodes raise concerns over possible adverse health impacts on civilians. However, claims of adverse effects have often been controversial, and current summaries are limited in geographical coverage and quality assessment.

**WHAT THIS STUDY ADDS**

- First systematic review of the literature for all LMICs, providing a more structured evaluation of causality and mechanisms.
- A significant proportion of studies reporting impact estimates (21/27) consistently detects adverse effects on health and health systems. A thematic narrative reveals possible dimensions and mechanics of exposure.

**HOW THIS STUDY MIGHT AFFECT RESEARCH, PRACTICE OR POLICY**

- Substantial limitations of the evidence base can be addressed by a combination of targeted data collection and quasi-experimental techniques. Civilian harm can be prevented by the adoption of a risk assessment framework based on prospective data collection and monitoring.

## Introduction

Economic sanctions (henceforth ‘sanctions’ or ‘embargo’) are restrictions to customary commercial and financial ties imposed by one or more states on a target, usually a state, to induce change in its political conduct or constitution.^1–5^ Measures adopted reflect the evolving opportunities to inflict economic losses in the global economy: prohibition of import and/or export of goods and services, either broad-based or limited to strategic commodities like weapons and natural resources; withholding of financial transactions (eg, foreign direct investment, military assistance, humanitarian or development aid); confiscation of assets and travel bans applied to listed persons and entities (Garfield^5^ p. 5 presents a richer typology).

**Figure 1:**
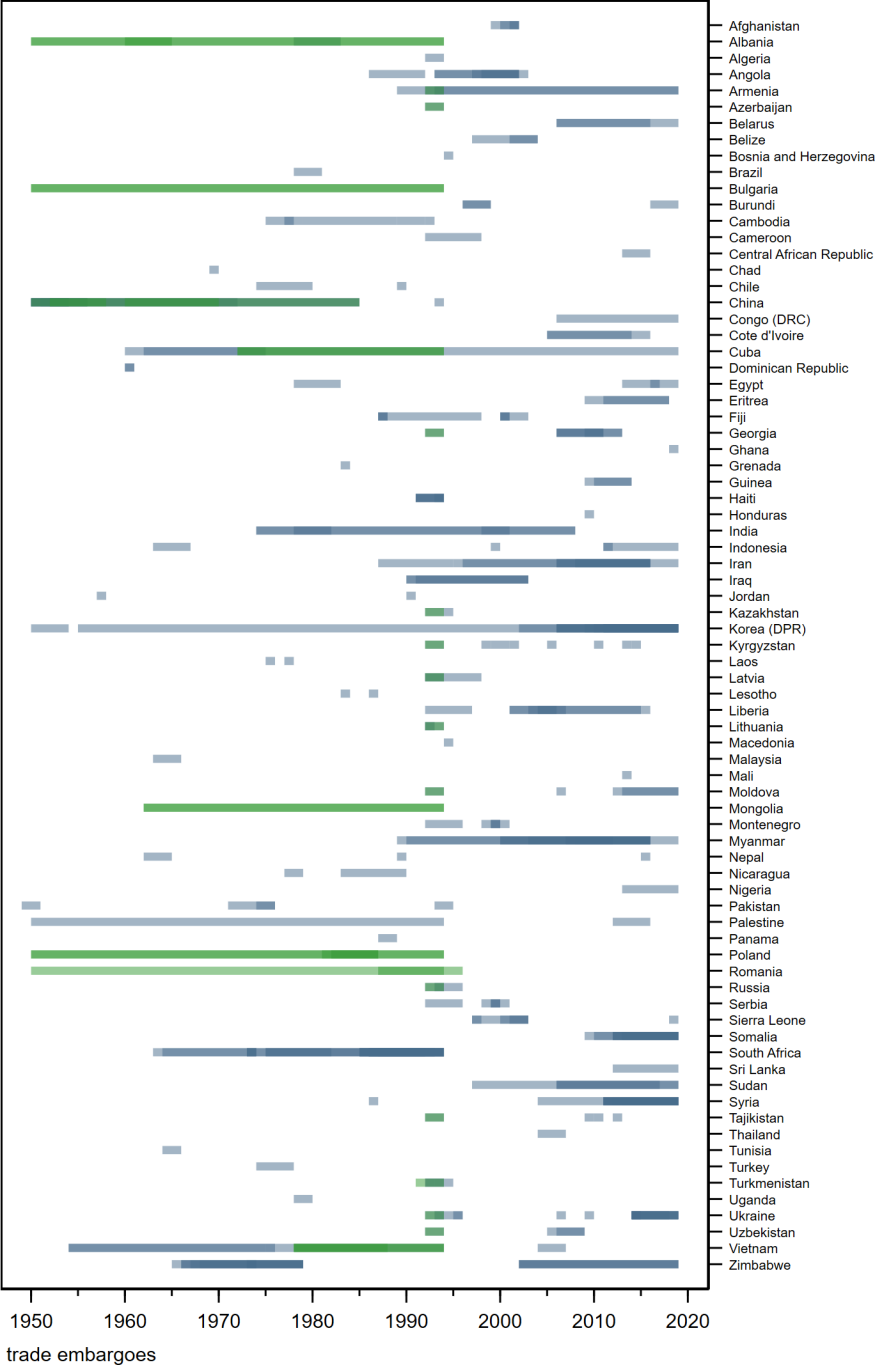
Timeline of economic sanctions in LMICs, 1950-2019. Green: Cold War-related. Greater colour intensity denotes overlapping measures. For notes see online supplemental figure A5.1.

Figure 1 plots episodes of trade sanctions against low-income and middle-income countries (LMICs) between 1950 and 2019 (for other types of sanctions, see online supplemental figures A5.1– A5.5). During the Cold War, alliances within blocks limited their scope^4^ and the UN Security Council used its power to impose ‘measures not involving the use of armed force’ including ‘the complete or partial interruption of economic relations’^6^ only twice (Rhodesia 1965–1979, South Africa 1962–1994). In the early 1990s the use of sanctions increased, as alternative or supplement to military intervention—and often portrayed as a benign method of dispute resolution, consistent in essence with human rights. However, accounts of salient episodes in Haiti (1991–1994) and Iraq (1990–2003) raised widespread concern that the burden of economic isolation might fall on civilians.^4 5 7^ These events mobilised the medical profession^8–11^; spawned controversy on the ethics of sanctions and the possibility to remove their potentially indiscriminate character^12–16^ and stimulated empirical research on the causes and consequences of sanctions.^17–19^ Recently, the issue gained renewed prominence as sanctions were imposed or tightened against Iran, Syria, Venezuela and Russia.

10.1136/bmjgh-2022-010968.supp1 Supplementary data

When societies choose their conduct during international disputes, adverse consequences for civilians should be considered. Any such welfare analysis will depend on societal goals, which may allow in various extent for the balancing of political and humanitarian considerations; on all (health and non-health) costs and benefits of sanctions; and on the costs and benefits of alternative options, which may include ‘going to war or leaving unpunished important crimes, such as genocide’.^5^ This latter point underlines the importance of counterfactual thinking in providing empirical ground to assist normative deliberation about sanctions. Recently, the WHO has commissioned a review of the evidence on whether—and if so, why—sanctions affect health and health systems in LMICs. This article presents the review’s key findings, improving on previous summaries in terms of comprehensiveness of coverage and providing a more systematic assessment of internal validity and mechanisms.

## Methods

### Search strategy, inclusion criteria and task division

Expecting a body of scholarship characterised by lack of specialised terminology, extensive grey literature and contributions from disciplines in which evidence synthesis is not well established, we opted for a broad-coverage, high-recall search strategy (details in online supplemental file A1). Moreover, as remarked by Petticrew,^20^ when uncertainty about effects cannot be measured by pooling information into precision estimates, the value of additional searches may diminish rapidly. We, thus, searched two large multidisciplinary databases, PubMed and Google Scholar (GS), covering the period January 1970–December 2021; and references identified in prominent papers or while retrieving records from systematic searches.

Accessible sources underwent two screening stages (title/abstract, full text), with the following inclusion criteria: English language; studies on (or inclusive of) countries classified as LMICs by the World Bank during the relevant period; and peer-reviewed publication or reports and working papers from UN agencies and research institutions, prioritising published versions. Anticipating a highly heterogeneous set of research methods, we adopted a two-tiered relevance criterion. Studies providing an ‘impact estimate’ (definition in online supplemental file A2) for any outcome domain related to health and health systems, however, measured, qualified as ‘core’ references. They were inspected in detail, and their findings form the building blocks of the review. A group of ‘non-core’ sources was defined, to preserve content deemed insightful in terms of subject-matter knowledge—including qualitative and mixed-methods studies, reviews, commentaries and correspondence. Identified records were exported or transcribed in table-formatted Excel spreadsheets. For GS, extraction and preliminary automatised removal of exact duplicates was facilitated by a dedicated software.^21^ Study characteristics were stored in separate datasets to generate summary statistics: bibliographic characteristics including editorial format, discipline and publication year; substantive and design features including geographical focus, type of contribution and research method (definitions in online supplemental file A2); characteristics of core studies, including the outcome variables employed, effect estimates, data structure and sample sizes. Visualisations and synthesis method were based on attributes without missing observations. Screening and data extraction were conducted independently on an even split of records by two authors (MPP and MS), with sample cross-validation. The review complies with the Preferred Reporting Items for Systematic Reviews and Meta-Analyses 2020 statement (see online supplemental file A6).

### Quality assessment and synthesis method

To assess evidence on their health consequences, sanctions can be usefully conceptualised as natural experiments (see ‘Conceptual framework’ box). The applicability of existing risk-of-bias protocols for observational studies^22^ of natural experiments and exposures is currently debated.^23 24^ We reviewed the ROBINS-I tool (Risk Of Bias In Non-randomised Studies - of Interventions) and identified various impediments to the construction of ‘target trials’ usable as quality benchmarks (see online supplemental file A3). Instead, we provide semistructured assessments based on the tool’s ‘bias domains’, deriving a simpler quality score (see online supplemental file A3). Given the heterogeneity of study designs, outcomes and effect measures, we eschew meta-analysis, and follow current guidelines^25^ to assess the existence of an effect through vote-counting—a more realistic and relevant objective in the analysis of sanctions (see online supplemental file A3). We then use these quality and effect-direction scores to visualise, and test hypotheses about, the quantitative kernel of the literature. As this approach to quality assessment is more heavily reliant on reviewers’ statistical knowledge and judgement, each rating was thoroughly discussed and established by consensus among the reviewing authors (MPP and MS). We see this score as a valuable tool to further characterise the ‘high-risk spectrum’ occupied by the virtual entirety of our sample of core studies. Finally, to explore possible causes of heterogeneity and leverage on existing background knowledge, the synthesised evidence was contextualised in the broader literature through a thematic narrative in the spirit of Ogilvie *et al*,^26^ who advocated a ‘dry stone wall’ approach to the integration of quantitative and qualitative information in population health research.

### Patient and public involvement

Neither patients nor the public were involved in any stage of the realisation of this review.

**Conceptual framework: sanctions as natural experiments**

Natural experiments are probabilistic events, with an unknown allocation mechanism, which are external to the subjects of the study population.^108^ When assessing consequences on civilians, this recent definition neatly applies to sanctions.

*Unknown allocation mechanism*. While the stated objectives of sanctions might include humanitarian considerations, and monitoring guidelines have been developed for this purpose,^97^ relevant data collection is not regularly incorporated into their implementation. Moreover, sanction policy-making is often opaque. As a result, public information on the determinants, timing and characteristics of sanctions is unavailable in advance and incomplete in retrospect. This contrasts with the classical experimental setting, where the probability that each unit is assigned to each of the study’s groups and associated exposures—the ‘allocation mechanism’—is specified prospectively by the evaluators. The allocation mechanism of sanctions is unknown, and their assessment lies firmly in the realm of retrospective observational studies.

*External exposure*. The risk of a country incurring sanctions is largely determined by political decisions and behaviours of the national and foreign governments. In particular, while governments may underestimate or overestimate the risk associated to a particular conduct, they are in general able to anticipate an order of risk. However, civilians in the country have typically little influence on these processes. Influence on governments' political behaviour is mediated by institutions and collective action, and is thus indirect and uncertain, especially in policy domains that influence the risk of sanctions. Civilians also face clear limits in influencing their own individual exposure if their country is sanctioned or anticipated to be so. The large size of targets—usually entire countries—limits the general ability of populations to avoid exposure by relocating, and individuals face obvious constraints in preserving for themselves formally interrupted economic ties. In sum, there are *prima facie* reasons to believe that civilians do not self-select into sanctions. Following econometric terminology,^109^ the imposition of sanctions is thus ‘external’ to (ie, not directly affected by) decisions by members of the population at risk. Finally, the implementation of sanctions usually proceeds much faster than the time needed for a country’s government and population to reorganise its economy and prevent all consequences. Instead, these strategic responses likely modify—and thus belong to—the effect of sanctions.

This definition, based on general subject-matter features, represents a useful heuristic. Its value lies in suggesting desirable elements of study design. The externality assumption must be supported by background evidence and operationalised by precise measurement of the timing and geographical coverage of sanctions. As externality corroborates but does not imply unconfoundedness,^108 109^ there is undiminished need to control for baseline differences in outcomes, likely caused by confounders not stemming from individual decisions, for example, the correlated shocks that often anticipate, accompany or follow sanctions—armed conflict, natural disasters and large-scale political and economic instability.

## Results

**Figure 2:**
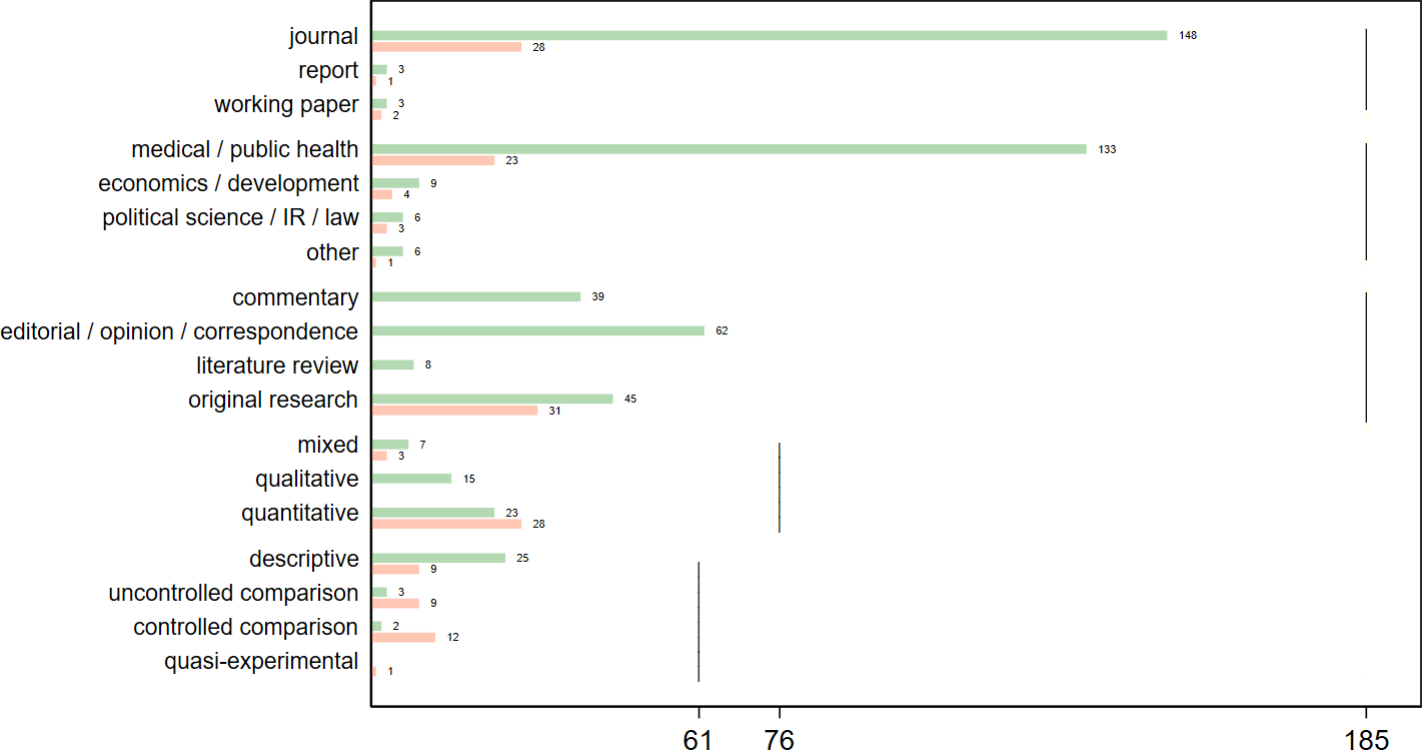
Included sources by editorial status, discipline, type of contribution and research method. Red: core studies; green: non-core studies. Definitions of categories in online supplemental file A2. Disciplinary sector attributed by journal, or by institutional affiliation of first author or publisher for grey literature and general-purpose journals. IR, International Relations.

**Figure 3:**
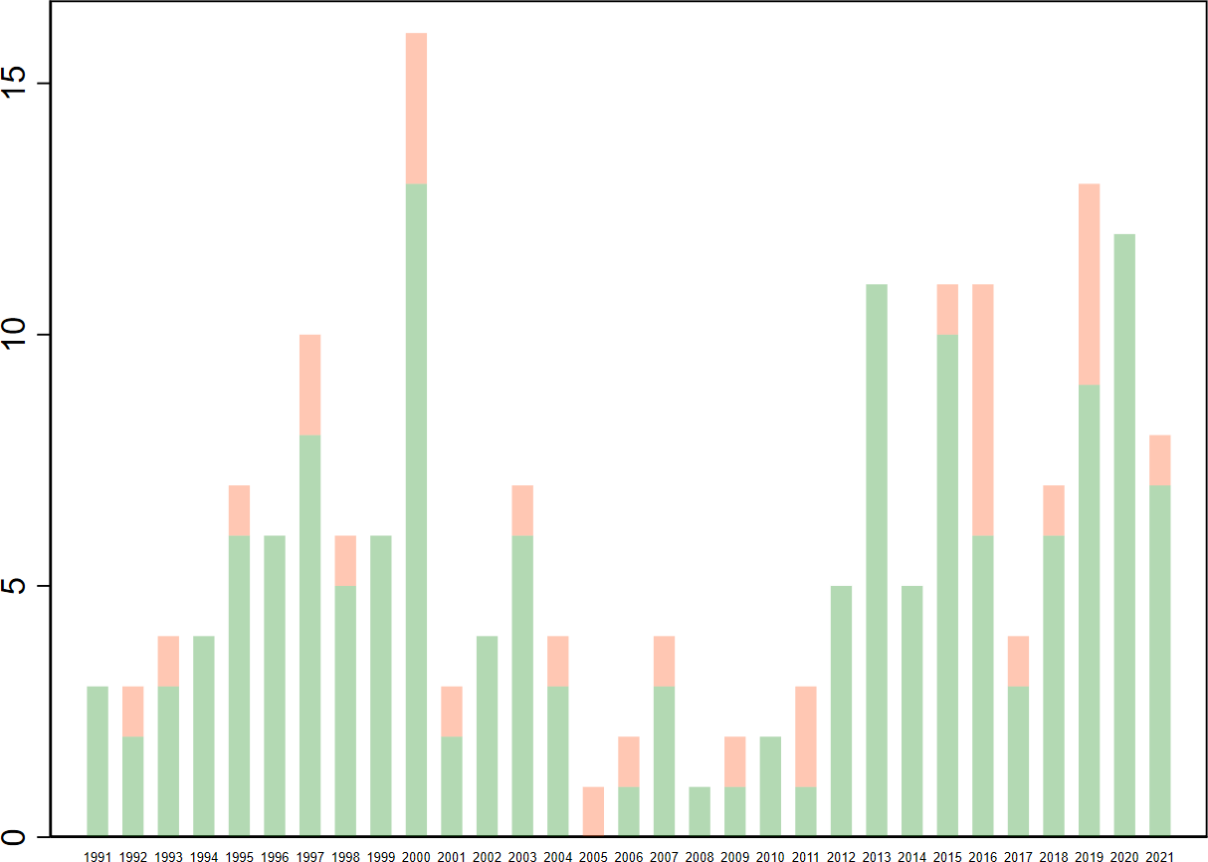
Included sources by publication year. Red: core studies; green: non-core studies.

**Figure 4:**
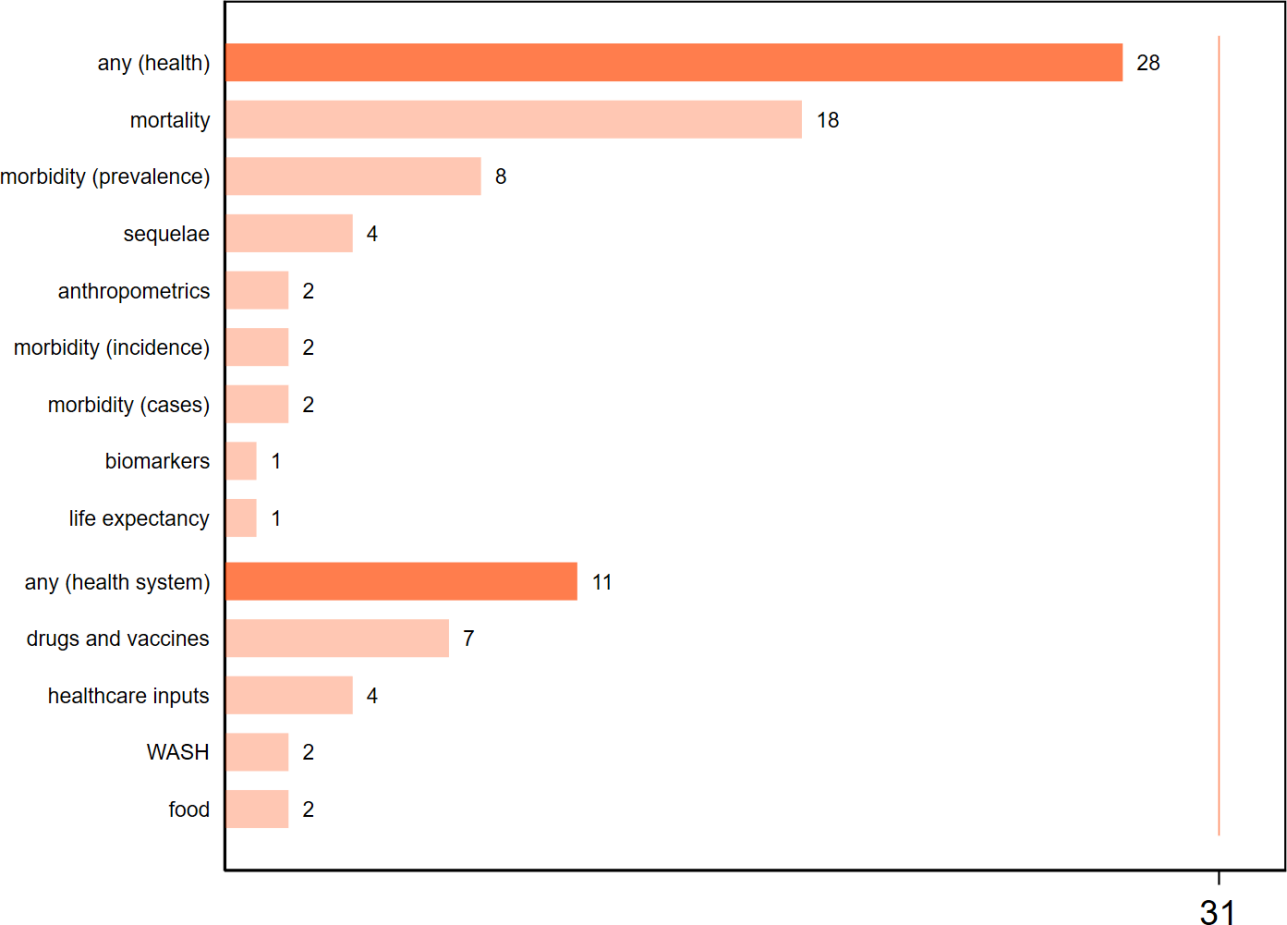
Outcome indicators in core studies, by measurement domain and subdomain. Outcome subdomains include the following specific indicators. Mortality: 1 month mortality rate (neonatal mortality rate); 1–12 months mortality rate (postneonatal mortality rate); infant mortality rate, under-3 risk of death, 1–4 mortality rate, under-5 mortality rate, under-6 mortality rate, under-2 risk of death 12 months after first visit, child deaths due to measles, maternal mortality rate, deaths due to cardiovascular diseases, 5-year survival rate after bone cancer treatment, all-cause mortality, all-age cause-specific mortality, under-15 crude HIV/AIDS-related death rate. Morbidity (prevalence): low birth weight (kg <2.5), type 2 diabetes, overweight/obesity, dental caries, HIV/AIDS among women, ‘stunting’ based on HAZ<2, ‘wasting’ based on WHZ<2, ‘underweight’ based on WAZ<2. Sequelae (health states caused by disease or injury): population in need of disaster relief, arthropathy score (haemophilia patients), annual bleedings (haemophilia patients), seizure frequency (epilepsy patients). Anthropometrics: 6–59 months HAZ, under-1 weight Z score, under-3 height Z score. Morbidity (incidence): new TB cases per 100 000 population, new HIV/AIDS cases among under-15 population. Morbidity (cases): diagnosed hepatitis B, diagnosed retinopathy. Biomarkers: serum ferritin (thalassaemia patients). Life expectancy: life expectancy at birth. Drugs and vaccines: city-level deliveries of hepatitis B vaccine, self-reported adherence to epilepsy treatment, self-reported ease of access to epilepsy treatment, self-reported (patient and doctor) access to thalassaemia treatment, facility-level availability of asthma medications, defined daily dose per 1000 population, unit dose per 1000 population per day, unit price of imported retail medicines in dosage form. Healthcare inputs: annual health expenditure per capita, X-rays per year, laboratory tests per year, size of national formulary, value of medical imports, annual blood transfusions (thalassaemia patients), public expenditure on disaster preparedness. Water, sanitation and hygiene (WASH): share of contaminated water samples, share of population with access to chlorinated water. Food: per capita calorie availability, per capita protein availability, free-sugars consumption. HAZ, Height-for-age Z score. WHZ, Weight-for-height Z score. WAZ, Weight-for-age Z score. TB, Tuberculosis.

Out of 185 included sources, 31 qualified as core (table 1, online supplemental figure A1.1). The literature mostly originates from peer-reviewed medical journals (figure 2), and tracks major sanction episodes in the 1990s (Iraq, Cuba, Haiti) and 2010s (Iran) (figure 3, online supplemental figure A5.6). Although most original research contains quantitative information, few studies estimate group differences or regression coefficients, and only one applies quasi-experimental methods (figure 2). Core studies assessed mainly health outcomes, especially early-age mortality and undernutrition. Studies of health system outcomes focused mainly on access to pharmaceuticals (figure 4).

**Table 1:** Direction of effects in core studies

Author/year	Effect 1	Effect 2	Effect 3	Effect 4	Effect 5	Effect 6	Effect 7	$D_{i}$
Al-Ani et al. 2011^29^	Mortality ▲							▲
Ali 2004^51^	Morbidity ▲							▲
Asadi-Pooya et al. 2019^74^	Drugs/vaccines ▲*	Sequelae ▲						▲
Ascherio et al. 1992^54^	Mortality ▲†							▲
Bundervoet and Verwimp 2005^32^	Anthropometrics ▲†							▲
Daponte and Garfield 2000^40^	Mortality ▲							▲
Garfield 2001^62^	Mortality ▲†	Mortality ▲	Mortality ▲	Morbidity ▲	WASH ▲	Healthcare ▲*		▲
Garfield and Leu 2000^110^	Mortality ▲							▲
Garfield and Santana 1997^56^	Mortality ▲†	Morbidity ▲	Morbidity ▲	Food ▲	WASH ▲	Healthcare ▲*	Drugs/vaccines ▲	▲
Ghiasi et al. 2016^73^	Drugs/vaccines ▲*							▲
Gutmann et al. 2021^42^	Life expectancy ▲†‡							▲
Karimi and Haghpanah 2015^35^	Sequelae ▲	Biomarkers ▲	Healthcare ▲§					▲
Kheirandish et al. 2018^31^	Drugs/vaccines ▲*¶							▲
Kim 2019a^95^	Morbidity ▲							▲
Kim 2019b^111^	Mortality ▲	Morbidity ▲						▲
McLean and Whang 2019^90^	Sequelae ▲‡	Healthcare ▲‡						▲
Mladenovich and Langeggen 2009^112^	Morbidity ▲							▲
Mulder-Sibanda 1998^39^	Mortality ▲	Morbidity ▲						▲
Parker et al. 2016^30^	Mortality ▲‡							▲
Reid et al. 2007^37^	Mortality ▲	Morbidity ▲						▲
Sharma et al. 2017^76^	Drugs/vaccines ▲*							▲
Asadi-Pooya et al. 2016^34^	Drugs/vaccines ▼*	Sequelae ▼						▼
Joury et al. 2016^113^	Morbidity ▼	Morbidity ▼†	Morbidity ▼	Food ▼				▼
Berggren et al. 1993^38^	Mortality ▲†	Mortality ▲	Morbidity ◄►**					◄►
Peksen 2011^28^	Mortality ◄►‡††	Mortality ▼‡††	Mortality ▲‡††					◄►
Petrescu 2016^33^	Mortality ▲	Anthropometrics ▲	Anthropometrics ▼					◄►
Zaidi 1997^114^	Mortality ◄►†							◄►
Ali and Shah 2000^115^								NA
Ali 2003^116^								NA
Dyson 2006^117^								NA
Zaidi and Fawzi 1995^118^								NA

Arrows denote harm (▲), benefit (▼) or conflicting evidence (◄►). $D_{i}$ is the overall direction-of-effect score. Studies are sorted in order of direction of effect, with four studies excluded due to data integrity concerns. Details on the construction of direction-of-effect scores in online supplemental file A3. Composition of outcome sub-domains in footnote of figure 4. Detailed version of the table in online supplemental file A5.

*Interpreted as measure of availability/supply.

†Synthesises effects for one or more combinations of mutually exclusive and collectively exhaustive sub-samples (eg, age classes, men/women, urban/rural).

‡Synthesises effects from alternative regression specifications.

§Interpreted as measure of need/demand.

¶Synthesises effects for multiple therapeutic or disease groups.

**Synthesises effects for different sub-periods, with no overall effect provided.

††Synthesises effects for alternative outcome or exposure datasets.

NA, not applicable;WASH, water, sanitation and hygiene;

In core studies, we uncovered a wide range of limitations in design and estimation (online supplemental figure A5.7). Problems of outcome measurement error and missing or non-comparable data likely reflect limited data collection capacity in LMICs, where under-reporting of vital events is common.^27^ Under-reporting may persist in survey-adjusted datasets (used by Peksen^28^), and share unobserved determinants with sanctions—thus requiring adequate control for baseline outcomes. Sanctions themselves may impair data quality by reducing resources and incentives to report, and inducing internal displacement that complicates survey design and alters the catchment population of health facilities. Hence, the increase in infant mortality under sanctions displayed by the registry of Haditha, Western Iraq is difficult to interpret.^29^ Sanctioned governments may manipulate data collection to inflate humanitarian costs, and four core studies were based on surveys later implicated in charges of fraud. To clarify the record, we reviewed these studies for separate discussion (online supplemental file A4), excluding them from the synthesis.

Available information was sometimes underused, and only one study^30^ performed systematic sensitivity analyses of exposure definition. Kheirandish *et al*^31^ performed structural break tests on time series of pharmaceutical availability, but did not explore the timing of the assumed change. A risk of selection bias affected studies where data collection could have been altered by sanctions-induced outmigration^30 32^ or changes in mortality in survey areas.^33^ Some studies minimised the issue using recalls or repeated observations.^30 34 35^ Confounding concerns were identified as the main problem, due primarily to omitted variables, but also to insufficiently justified regression models, limited sensitivity analyses or inclusion of ‘bad controls’^36^ (ie, covariates affected by sanctions). For example, Reid *et al*^37^ estimated the impact of sanctions against Haiti on mortality of young children enrolled at a health facility, controlling for undernutrition at enrolment. As sanctions could have altered the facility’s catchment population in terms of pre-existing nutritional status, this step could be useful. However, sanctions might have also causally affected nutritional status, which complicates the interpretation of estimated coefficients. Sanctions are often accompanied by unmeasured correlated shocks, which can confound or modify estimates (online supplemental figure A5.8). For example, Haiti faced widespread instability and political violence in the run-up to sanctions.^37–39^ Such instability could modify simple before-and-after estimates if it persisted during sanctions, or confound them if it subsided with time. Consistent with the latter, mortality in the facility study was higher both during and before sanctions vis-à-vis a postsanction period.^37^ In studies of Iraq, the First Gulf war is often a likely confounder and almost always a plausible effect modifier. Exceptionally, one study^40^ exploited the short period between the imposition of sanctions and the onset of military operations. In general, almost all studies suffer from important limitations in multiple bias domains. As clarified in the discussion below, some of these weaknesses have readily available remedies, whereas others are likely to reflect more fundamental subject-matter challenges—such as the complexity of the exposure, its low-frequency and context-specific nature, and the likely broad range of possible causal pathways.

Among 27 synthesised studies, 21 reported consistent adverse effects of sanctions on examined outcomes (table 1, online supplemental figure A5.9 and table A5.1). This proportion is significantly higher than expected assuming no effect and a range of probabilities of false positives and negatives, from an even chance up to a 10 percentage-points greater likelihood of false positives—which might stem from publication or reporting bias (online supplemental table A3.1). Effect direction appears unrelated to assessed quality, focus on earlier sanction episodes and publication period (online supplemental table A3.2, A3.3 and figure A5.10). Perhaps reflecting better data, study quality was higher for recent episodes, while no association was found with publication period.

## Discussion

**Figure 5:**
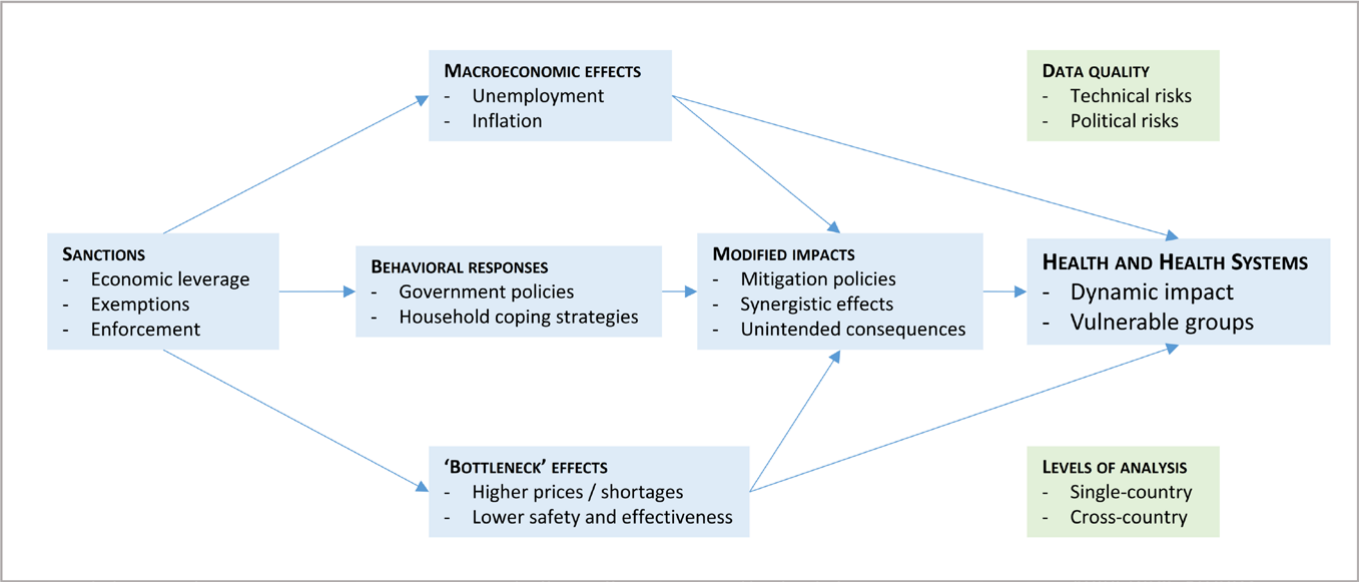
Structure of the thematic narrative and causal model of sanction impacts on health and health system. Causal diagram in blue.

In what follows, we contextualise impact estimates in the broader literature through a thematic narrative (online supplemental table A5.2 details non-core studies). Its structure, developed inductively in the early stages of the review, provides an analytical map of the subject (figure 5). The causal model nested in the figure represents a proposed explanatory framework, highlighting two features of sanctions that have been taken as markers of complexity in health interventions research^41^: multidimensionality and multiplicity of channels.

### Multidimensionality of sanctions

Sanctions are not homogeneous constructs and can vary, *inter alia*, in scope, restrictiveness and enforcement. Impacts may, therefore, differ across these dimensions, and change as measures are tightened or relaxed.

For example, sanctions by large trade partners and multilateral organisations can be expected to exert greater damage.^4 5^ Consistent with this intuition, Gutmann *et al*^42^ report that UN sanctions have a larger negative impact on life expectancy than US sanctions. A study of under-5 mortality^28^ under US and multilateral sanctions found an opposite pattern, but arguably provided weaker adjustment for baseline differences between sanctioned and non-sanctioned countries. As neither study allows for separate baselines according to sanctioning parties, which may target systematically different countries, more evidence is needed.

Most sanctions contain clauses exempting essential commodities, including food and medications. However, qualitative research suggests that, due to implementation frictions, substantial trade barriers often remain. Trade in exempted items requires participation in a licensing and monitoring system, which raises transaction costs. In 1992, tightened US sanctions against Cuba required federal inspection of all shipments on Cuban territory.^43^ Ambiguous definitions of exempted categories entail a risk of involuntary violation, further raising expected costs and discouraging risk-averse firms—an ‘overcompliance’ effect noted in reports on Syria and North Korea.^44–46^ Definitional ambiguity often surrounds ‘dual use’ items—with both military and civilian applications. In extreme cases, claims of dual use invoked to justify exclusion from exemption lists turned out to be unfounded. Examples include ‘the denial of purchasing rights for spare parts for breast X-ray equipment for Cuba for the stated reason of the potential for ‘medical terrorism’ and […] permits to import nitro-glycerine paste for Iraqi angina patients due to the mistaken belief that the medicine had a potential application in building bombs’ (p. 24).^5^ Recently, Iranian professional bodies raised concerns over the banning of intermediate inputs for radiopharmaceuticals.^47 48^ In India, World Bank funds blocked under UN sanctions were later unfrozen ‘thanks to a liberal interpretation of these loans as humanitarian aid’,^49^ suggesting political bargaining over operational terminology. Exemption-related costs and risks also affect providers of complementary services, for example, trade banking and shipping insurance, which reportedly constrained Iran’s pharmaceutical imports.^50^ Where health and social services are publicly provided, bans on engagement with government personnel may further hamper deliveries of exempted items.^38 45^

Despite extensive discussion, exemptions lack systematic measurement to operationalise them as possible determinants of the ‘severity’ of sanctions. Two studies of Iraq^51 52^ report annual variations in child morbidity across the implementation of major exemptions through the Oil-for-Food Programme, but no discontinuity is defined. As data on other legal and administrative features of scope and enforcement is also unavailable, quantitative studies assess severity through various proxies, eg the estimates of ‘sanction-related economic losses’ computed by Hufbauer *et al*.^3^ This outcome-based measure may usefully capture sanctions’ macroeconomic impacts (see below p 11), and its documented association with under-5 mortality^28^ fits existing evidence on early-age mortality and income shocks in LMICs.^53^ However, it is unlikely to identify the severity of sanctions separately from other effect modifiers. Finally, the life expectancy impact of US sanctions has been shown to attenuate for more distant countries.^42^ Distance likely captures variation in trade volumes affected, but may also reflect differences in implementation, for example, if policy makers treat distance as a constraint or a factor to offset. These complexities reinforce the case for direct measurement of structural characteristics of sanctions in future data collection efforts.

### Channels of impact

Different impacts across episodes may also reflect the multiplicity of possible causal pathways. While quantitative studies emphasise total effects with limited analysis of mediators, the broader literature suggests two types of channels: changes in supply and demand, and deliberate behavioural or policy responses.

#### Total and partial impacts

Most core studies estimate impacts on population health indicators—typically affected by many factors. Cross-country analyses found sizeable adverse effects of UN and US sanctions on life expectancy,^42^ and of US sanctions on under-5 mortality.^28^ In single country studies, sanctions were associated with increases in undernutrition in Burundi,^32^ infant mortality in the Democratic Republic of Congo,^30^ under-3 mortality in Haiti,^37^ and mortality for various early-age groups in Iraq.^40 54^

Some studies explored mediators for these effects. In their study of US sanctions against guerrilla-controlled mining in Congo, Parker *et al*^30^ model the estimator as a triple difference, interacting indicators for implementation period, areas affected and proximity to mines. Results suggest that sanctions increased infant mortality by reducing mining-related incomes. Other strategies require careful examination. In the same study, the robustness of estimates to additional control for armed clashes in children’s locations is interpreted as lack of mediation through reduced guerrilla activity. In another study, Bundervoet and Verwimp^32^ replace the sanction indicator with food price indices, and see the negative coefficient obtained as evidence that sanctions increased child stunting through food prices. In both cases, no explicit consideration was given to possible determinants of proposed mediators other than sanctions—for example, persistent and long-run effects of Burundi’s civil war on food prices. Auxiliary regressions were sometimes performed, relating to sanctions candidate mediators: primary healthcare and bed net use (mediating infant mortality rate)^30^; under-5 mortality, cholera deaths and government health expenditure (mediating life expectancy).^42^ Although suggestive, these single-equation assessments of multiple mediators overlook possible reciprocal influences. This affects interpretation: if child mortality increases under sanctions due to a cholera outbreak caused by reduced health spending, ring-fencing health spending in similar instances may be crucial. However, if cholera deaths rise for separate reasons, eg, a shortage of water treatment chemicals, different policy implications may be warranted. Recent advances in mediation analysis may assist future work in addressing these limitations.^55^

#### ‘Bottleneck’ effects

Field observations and correspondence often report large reductions in the supply of specific health inputs under sanctions, leading to higher market prices, queues for public provision and in extreme cases complete unavailability. Items mentioned include chemicals used as laboratory reagents, to chlorinate water or synthetise proteins^56–58^; fertilisers and pesticides^38 57^; prosthetic materials^59^; barium for X-ray machines^60^; medical textbooks and online courses^57 61^; and general-purpose energy and mechanical goods affecting, *inter alia*, ambulance transport,^56 58 62 63^ blood and vaccine storage.^38 58 64 65^ As an input’s price increases, households and health-sector organisations will be expected to look for cheaper substitutes, and adjust expenditure priorities to the new, reduced level of purchasing power.^66^ As in those LMICs at risk of experiencing sanctions substitution possibilities may be limited, for example, due to intellectual property rights,^43^ and price increases may be large relative to income for many households, bottlenecks may substantially constrain health-seeking decisions.

Evidence for bottlenecks is scanty. A somewhat consistent picture emerges for Iran, where shortages and higher prices of pharmaceuticals have been frequently reported on imposition of additional sanctions.^67–72^ Reduced availability was documented for asthma medications in a survey of Tehran’s community pharmacies,^73^ and for 13 out of 26 pharmaceuticals in a time series analysis of national supplies.^31^ In both cases, effects were larger for imported final products, but domestic medications with imported content were also affected. A facility-based study^34^ of epilepsy patients found no significant changes in self-reported adherence after tightened international sanctions, but another study^74^ on a similar (and possibly overlapping) sample found that, under renewed US sanctions, patients on imported medications were more likely to report reduced availability and the occurrence of seizures. Finally, a facility-based study of haemophilia and thalassaemia patients documented a significant worsening of clinical outcomes under tightened international sanctions vis-à-vis presanction trends.^35^

These studies rely on binary exposures poorly suited to Iran’s complex sanction timeline, and make minimal use of background information and sensitivity analyses to corroborate their comparisons. Recent time series work in economics^75^ illustrates the possibility of using continuous measures of sanction severity. Nevertheless, these findings can be seen as snap-shots of a complex dynamic impact, collectively suggesting shortages of final and intermediate products. Even more direct evidence of a bottleneck effect, a spike in medication unit import prices, was documented for Nepal,^76^ where the 2015 Nepal-India border blockade was estimated to have caused 22.3 million USD extra costs for ‘retail dosage-form’ drugs.

When sanctioned items have cheaper substitutes, their affordability may reflect lower effectiveness or safety. Anecdotal evidence includes a series of cases of blindness after eye surgery attributed to substandard equipment in Iran^77^; unintentional poisoning from caustic soda used as surrogate for soap in Cuba^56^; and worsening dietary quality in Haiti,^38^ Serbia-Montenegro^62^ and Cuba, where lack of animal proteins was later implicated in an epidemic of neurological disorders.^57 78^ It has been argued that shortages induce consumers to accept risks associated to expired, counterfeit, or mishandled pharmaceuticals,^50^ stimulating the emergence of black markets, a frequently noted phenomenon under sanctions.^38 50 62 72^ This in turn has raised concerns about the spread of antimicrobial resistance.^63 79 80^ However, no impact estimate for similar hazards was found.

#### Macroeconomic effects

Reductions in trade can depress export demand and revenues, raise production costs and reverberate throughout domestic sectors leading to a contraction of economic activity. Recessions can, in turn, affect population health.^81–83^ For this macroeconomic channel to operate, various conditions must hold:

1. A sufficiently severe shock, in terms of volume and composition of flows involved and degree of integration between importing/exporting firms and other producers—as in Iraq, where revenues from a single export industry (oil) played a crucial role in financing domestic investment.^84^
2. Limited ability of firms to absorb the shock by establishing alternative trade channels—for example, due to market entry barriers and greater transport costs.^85^
3. Inability or unwillingness of sanctioned governments to counter the slowdown by adequate macroeconomic policies.

In a related literature, studies of sanctions’ economic outcomes corroborate this sequence, documenting lower firm profitability,^86^ output and employment,^75 87^ and higher poverty rates.^88^ In the reviewed literature, field reports mention declines in industrial output and investment, especially in exports, and high inflation and unemployment.^38 56 62 84^ As mentioned previously, a study of US sanctions in Congo suggests that mining-related incomes declined, affecting healthcare use.^30^

In sum, plausible theory and evidence suggest that both bottlenecks and macroeconomic effects can occur. Their relative contribution, and the possible mediating role of pre-existing institutional factors affecting income distribution and inequality, and access to health inputs, remain largely unknown.

#### Responses to sanctions: policies and institutions

The consequences of sanctions may extend beyond changes in prices and quantities in markets and public sectors, and include more complex policy and societal responses. A long-standing position in political science argues that sanctions frequently backfire because governments are able to offload costs on internal opponents and cement consensus through feelings of national solidarity.^17 18^ Somewhat analogously, reviewed studies suggest that sanctioned governments can reorganise existing resources to mitigate health impacts.

A comparative assessment^89^ of three well-studied episodes argued that Haiti and Cuba were able to maintain ongoing secular declines in infant mortality, despite rising undernutrition^39^ and mortality^37 56^ in older children, through targeted food supplementation, community-based health education, sustained promotion of breast feeding and liberalisation of tightly regulated agricultural markets.^56^ In Iraq, instead, large increases in infant mortality in the immediate aftermath of sanctions^40^ heralded persistently high levels of undernutrition^52^—partly attributed to a healthcare model biased against primary services and prevention.^5 89^ Reports on Serbia-Montenegro^62^ and Iran^50^ suggest that sanctions may induce governments to alter regulatory policies, including price subsidies, in ways that benefit special interest groups, for example, well-connected pharmaceutical companies, potentially aggravating shortages or impeding equitable access.

Two cross-country analyses provide more systematic information. McLean and Whang^90^ report that, under sanctions, spending on disaster preparedness declines 8%–18%, while disaster-related economic losses and population affected increase 88% and 95%, respectively. They argue that sanctions harden the targeted government’s budget constraint and simultaneously signal a risk of armed conflict—prompting cuts to ‘low-visibility’ civilian spending. Some effects are smaller for low-income countries, perhaps reflecting lower data quality; and strategies to control for confounding fall short of ruling out that, for example, results are affected by country-specific baseline trends, or differences in disaster severity. Notwithstanding these limitations, the study breaks new ground in exploring government responses, proposing a plausible theory. Gutmann *et al*^42^ model government responses as the principal component of three indicators of institutional quality, showing that impacts on life expectancy are concentrated in countries with worse ‘political environments’. While a causal interpretation of this result is plausible, differences in political environment may also track unobserved differences in sanction characteristics—for example, tougher sanctions being imposed against less democratic countries. Deepening interpretation with cross-country studies is complicated by the coarseness of available indices, which may be poorly predictive of long-term health system models and relevant short-term policies.

#### Responses to sanctions: households’ coping strategies

The literature confirms the well-established importance of household behavioural responses to resource and health shocks in LMICs.^91^ Qualitative studies of Cuba,^58^ Serbia-Montenegro,^62 63^ Haiti^64^ and Iraq^84^ identify a number of coping strategies: changes in dietary habits, frequency of meals and resort to ‘famine foods’ (Iraq, Haiti); urban-to-rural migration to seek food and farmland (Haiti, Serbia-Montenegro); ‘distress sales’ of land, livestock and consumer durables (Haiti, Iraq); changes in living arrangements, including the consolidation of households, partitioning of dwellings and sharing or outsourcing of food preparation (Haiti, Serbia-Montenegro); disruption of family-formation by postponement of marriages, cohabitation and planned fertility (Haiti, Iraq, Serbia-Montenegro); school dropouts (Iraq, Haiti) and an increase in informal income-generating activities, including prostitution, smuggling and crime (Cuba, Haiti, Serbia-Montenegro, Iraq). These individually adaptive strategies can lead to unintended societal consequences: dissaving in bad times provides limited benefits, while overcrowded housing, migration and informality all carry potential health hazards and may produce a mismatch between population and local infrastructure.

Coping strategies are essentially unmeasured, and in two core studies,^30 32^ estimates might have been confounded (or modified) by unobserved migration, selecting into the exposed group children facing greater independent risks (or impacts). Parker *et al*^30^ employ mother-level fixed effects, excluding bias due to mothers experiencing below-average infant mortality fleeing from targeted to other villages within the survey area. However, they cannot exclude that similar movements left behind, and exposed, mothers facing worsening infant mortality—for example, due to a greater impact of lower resources at increasing parities. These estimates likely retrieve the impact of sanctions specific to those left-behind mothers. While this is useful to highlight groups with limited coping opportunities, only an understanding of mobility and other survival strategies will allow their prospective identification.

In conclusion, it is the consistency between government and household responses which likely determines their joint effectiveness in mitigating adverse changes in incomes and prices—an issue that requires more attention from researchers and policy makers.

### Short-term and long-term effects

Variation in sanction characteristics and in the timing of alternative channels can give rise to a complex dynamic impact. Its estimation is difficult, and attempts are bound to be marred by uncertainty and controversy, as an in-depth look at the Iraqi episode demonstrates (online supplemental file A4). Yet, if needs evolve under sanctions as they do after other societal shocks (eg, natural disasters and armed conflict), such knowledge may be useful in designing effective mitigation. A preliminary issue is whether impacts display a cumulative pattern. This seems not to be generally the case in the study of life expectancy,^42^ where regression estimates of the effect of one additional year of sanctions are insensitive to the use of a non-linear functional form.

### Impact on vulnerable groups

A final important theme in the literature is vulnerability—the set of factors predisposing certain groups to greater adversity for a given hazard.^92^ The main vulnerability investigated in the literature, again with considerable gaps, relates to differential health outcomes across men and women. Differences generally arise from a combination of genetic, developmental and cultural determinants,^93^ and may be modified by sanctions. Gutmann *et al*^42^ report a larger average adverse impact on life expectancy for women, and a larger impact of an additional year under sanctions for men. Hence, impact differentials are not the same in sanctions of average and (sufficiently) above-average duration. This result matches current evidence of attenuated female longevity advantage during mortality crises,^94^ and suggest an interplay between multiple time-varying factors. Evidence for early-age mortality is limited to one study,^37^ reporting insignificant interactions between child sex and exposure to sanctions. A cross-country study^95^ reporting an elevated female share of HIV-AIDS prevalence under sanctions has important limitations, but is consistent with qualitative evidence on high-risk coping strategies.^38 56 58^ In Cuba’s epidemic of neurological disorders, a study sampling all severe cases in a region ended up with two-thirds male patients,^78^ but the role of sanctions in the outbreak remains conjectural. Future studies should respond to calls to incorporate sex and gender into global health,^96^ striving to explore how sanctions affect men and women specifically.

Evidence on other vulnerable groups is either anecdotal, for example, a possible neglect of the elderly in Cuba’s mitigation policies^56^; or implicit in studies of patients on advanced treatments, emphasising their peculiar risks.^34 35 74^

## Conclusions

A large, heterogeneous literature investigates the impact of economic sanctions on health and health systems in LMICs. Few studies quantify those impacts addressing challenges to causal inference. Looking at the proportion of studies consistently reporting harmful effects, the evidence strongly suggests the existence of adverse consequences. The finding is in line with previous reviews, but stems from a more comprehensive search strategy and state-of-the-art evidence synthesis methods.

As our thematic synthesis reveals, however, generating impact estimates consistent with a plausible causal model is challenging. Sanctions are multidimensional hazards, and their impact varies depending on many factors, including the economic leverage of the sanctioning party, the exemption system in place and the evolution of measures over time. Impacts can originate from a combination of changes in prices and quantities of specific health inputs; a general decline in incomes due to inflation and unemployment; and responses by governments, communities and households—which may exert subtle and contradictory influences. Learning about these factors can improve mitigation policies, on which insufficient attention has been paid by existing research. While inadequate data currently hampers the design of accurate studies, we also observed room for straightforward improvements. In general, regression-based studies can readily benefit from incorporating quasi-experimental techniques and new methods for effect modification and mediation.

Additional caution in interpreting these findings stems from limitations of the review itself. First, our simplified risk-assessment procedure relies on reviewers’ statistical judgement more heavily than the original tool. Second, while effect heterogeneity appears to be important, we could only explore it through a structured narrative. While we believe these methods to be reasonable adaptations to key characteristics of the literature and subject-matter, they do generate additional uncertainty over the results. It is also possible that outlying results were overlooked, if published in languages other than English or contained in grey literature that was neither retrieved nor fully incorporated into retrieved publications. Moreover, due to little pre-existing methodological guidance on the subject matter, no preregistered protocol was prepared. Finally, systematic reviews, even if adequately implemented, can be fruitfully complemented by other assessments, such as Delphi-method interviews of expert panels—which exceed the scope of our contribution but are likely to cast further light on the topic.

A more fundamental solution to the many limitations of this research domain, however, likely depends on its transformation into a routine monitoring activity, enabled by an adequate institutional framework. The systematic application of prospective assessment, and the extent of information exchange between governments and evaluators that goes with it, would shift research activities, at least in part, away from *ex post* documentation and towards risk reduction and control. Evaluators would face new decisions, first and foremost about data collection. While a full discussion lies beyond the scope of this review, a few points can be highlighted here with respect to the aim of reducing bias.

First, any assessment will have to begin with a comprehensive retrieval of baseline information, including the scoping of existing qualitative and quantitative data such as knowledgeable local actors, censuses, administrative registers, sample surveys—with an emphasis on determining their usefulness (eg, if a survey’s target population is known); a country analysis, documenting relevant pre-existing temporal and spatial trends; and an analysis of sanctions to establish plausible impact channels to investigate.

New data will have to be collected for a minimum sufficient set of indicators, which might change throughout the episode to track the evolving set of possible impacts. The expert process needed to identify such set might benefit from the recommendation of a '4+4 human security measurement domains' set made by the only fully developmend sanction assessment methodology we identified^97^; and from initiatives in humanitarian response,^98 99^ where a similar issue arises.^100 101^ When a useful baseline exists, data collection must aim at ensuring whatever extent of comparability is possible–for example, replicating the methodology of a cross-sectional survey fielded before sanctions to generate a pseudopanel. Credible reconstruction of a *de facto* baseline through fast rollout of data collection might be possible in special cases–for example, for outcomes that change gradually or that can be measured with well-formulated, pretested recalls. Most other data can probably be best acquired under sanctions through compact, high-quality panel surveys of households and health facilities. Given survey resources, the utility of larger samples must be carefully weighted against that of investing in training and tracking capacity to minimise nonresponse and attrition. Methods might have to incorporate safeguards against respondent mistrust and political pressures, for example, a pharmaceuticals price survey may be implemented by rotating panels of pharmacies if attempts to exaggerate shortages are suspected.

In analysing the data, evaluators can gain insights from multiple approaches to causal inference: graph-theoretic,^102 103^ counterfactual,^104 105^ structural^106^ and qualitative.^107^ General concepts that must be considered include the existence of mediators; the difference between sufficient and necessary causes, and between practical and statistical significance; time-varying, lagged and persistent effects. Focus should lie on the identification of causes of practical significance that are modifiable and related to sanctions—although importantly, this relation need not be direct and may depend on the conduct of the sanctioned country. In issuing recommendations to sanctioning and sanctioned countries, evaluators might consider a principle of redundancy, whereby health risks are best managed if more parties act than is strictly necessary.

The problem of who is to implement such assessments remains outstanding. In the past, assessments have been entrusted on a case-by-case basis to entities such as the UN Secretariat, *ad hoc* expert panels and the UN Office for the Coordination of Humanitarian Affairs.^97^ The creation of a permanent body will have to overcome important technical and political barriers, although trends such as the growth of south-south trade might favour this solution. Sanctions are increasingly equipped with incentives to elicit compliance from third parties, such as extraterritorial provisions and diplomatic exceptions or compensations.^1^ To the extent that the practice reveals an increasingly pivotal role of regional state actors in determining the viability of sanctions, these states might support the creation of a body that can further improve their bargaining position in negotiating adequate protection of exempted trade. Whether a critical mass of supportive countries can be reached, and whether such a mechanism could distribute enough material and reputational costs and benefits to avoid being undone by bilateral actions or dishonest communication, is a pressing problem to be addressed with intellectual and political ingenuity.

We conclude by stressing that the existing evidence, despite clear limitations, should command serious attention by the international community. At a minimum, it strengthens the expectation that sanctions can hurt civilian populations. Ultimately, only the incorporation of risk assessment procedures based on prospective data collection into the administrative machinery of sanctions can certify claims that civilians are adequately protected. The failure of the community of states to evolve a legal custom of reciprocal monitoring against these hazards represents a self-imposed obstacle on the road towards ‘Health for All’ undertaken in Alma Ata, more than 40 years ago.

## Data Availability

All data relevant to the study are included in the article or uploaded as online supplemental information.
