# Supplementary material for: The impact of economic sanctions on health and health systems in low-income and middle-income countries: a systematic review and narrative synthesis
Source: BMJ Glob Health. 2023 Feb 9;8(2):e010968. doi: 10.1136/bmjgh-2022-010968 (PMC9923316; doi:10.1136/bmjgh-2022-010968)
Supplement: Supplementary data [file bmjgh-2022-010968supp001.pdf]

Supplementary materials

Table of Contents

A1: Search strategy 1
A2: Classification of studies by research method 6
A3: Assessment of study quality and synthesis of effects 7
A4: Case study. The dynamic impact of sanctions in Iraq 11
A5: Additional graphs and tables 14
A6: Research reporting guideline checklists 108
A7: Author reflexivity statement. 112
References 114

A1: Search strategy

We undertook a broadly defined PubMed search, comprising the union of the words "sanctions" and "health" in the title or abstract. The searches were carried out on 19/10/2019 and 9/12/2021. Search commands used were:

((Sanctions[Title/Abstract]) AND health[Title/Abstract]) AND ("1970/01/01"[Date - Publication] : "2019/10/19"[Date - Publication])

((Sanctions[Title/Abstract]) AND health[Title/Abstract]) AND ("2020/01/01"[Date - Publication] : "2021/12/9"[Date - Publication])

Recent assessments of the alternative properties of existing search systems shows that Google Scholar is unsuitable as a primary search system, but is useful as a complement.[1] Google Scholar searches are detailed in Table A1.1, with green shading denoting selected searches. In the course of retrieving sources from the first Google Scholar search, various additional sources of potential relevance were identified and screened for inclusion (Table A1.2). No such incidental findings occurred when retrieving sources from the second Google Scholar search. Some sources first retrieved in unpublished format were later found in published format. Priority was accorded to published versions unless this conflicted with other inclusion criteria – eg the published version of Bundervoet and Verwimp (2005) dropped the analysis of sanctions entirely (Table A1.3). A small amount of sources were included despite failing to satisfy inclusion criteria, with associated justification for the decision (Table A1.4). Table A1.5 presents the PRISMA flowchart of the screening process. Study authors were queried via mail for additional information when needed.

Table A1.1: Google Scholar searches. Green shading indicates searches selected for the review.

search terms	time window	N	search date
allintitle: sanction|sanctions|embargoes|embargo health|mortality|humanitarian|impact|impacts|nutrition|nutritional|malnutrition|expectancy|survival|infection|infections|noncommunicable|incidence|prevalence|"risk factors"	1970 – 2019	999	21/10/2019
allintitle: sanction|sanctions|embargoes|embargo health|mortality|humanitarian|nutrition|nutritional|malnutrition|expectancy|survival|infection|infections|NCDs|incidence|prevalence|"risk factors"|healthcare|medicines|drugs|"chronic conditions"	1970 – 2019	500	21/10/2019
allintitle: sanction|sanctions|embargoes|embargo health|mortality|humanitarian|nutrition|nutritional|malnutrition|expectancy|survival|infection|infections|noncommunicable|incidence|prevalence|"risk factors"	1970 – 2019	453	21/10/2019
allintitle: sanction|sanctions|embargoes|embargo health|mortality|morbidity|"humanitarian impact"|"humanitarian impacts"|nutrition|malnutrition|"life expectancy"|survival|"death rate"|infection	1970 – 2019	323	21/10/2019
allintitle: sanctions|embargoes|embargo health|mortality|morbidity|"humanitarian impact"|"humanitarian impacts"|nutrition|malnutrition|"life expectancy"|survival|"death rate"|infection	1970 – 2019	306	21/10/2019
allintitle: sanction|sanctions|embargoes|embargo health|mortality|humanitarian|nutrition|nutritional|malnutrition|expectancy|survival|infection|infections|NCDs|incidence|prevalence|"risk factors"|healthcare|medicines|drugs|"chronic conditions"	2020-2022	100	18/3/2022

Table A1.2: incidental findings in Google Scholar search.

Found	while searching for
1. https://doi.org/10.1111/j.1365-3156.2011.02941.x	*wrong link saved*

2.	https://www.ncbi.nlm.nih.gov/pmc/articles/PMC3464859/	
3.	https://www.jstor.org/stable/29768138	https://www.ncbi.nlm.nih.gov/pubmed/9354048
4.	https://pcr.uu.se/research/smartsanctions/literature/sanctions-a-bibliographic-manual/	https://europepmc.org/abstract/med/9695765
5.	https://www.jstor.org/stable/4418840	http://www.casi.org.uk/info/garfield/dr-garfield.html
6.	https://www.jstor.org/stable/4329469	
7.	https://www.nejm.org/doi/full/10.1056/NEJM199704243361711	https://www.bmj.com/content/315/7120/1393.2
8.	https://www.bmj.com/content/315/7120/1474	
9.	https://www.bmj.com/content/315/7120/1463.2	
10.	https://www.thelancet.com/journals/lanonc/article/PIIS1470-2045(18)30751-4/fulltext	https://www.europeanleadershipnetwork.org/wp-content/uploads/2018/11/FINAL-ELN-BB-HSPV-Policy-Brief-271118-for-ONLINE.pdf
11.	http://gozaresh-nakhande.ir/ref/3/27/sanctions_medical_supply_shortages_in_iran.pdf	
12.	https://www.nature.com/articles/520157b	https://www.nature.com/articles/520623c
13.	https://www.nature.com/articles/d41586-017-08580-z	
14.	https://www.ncbi.nlm.nih.gov/pmc/articles/PMC5568715/	https://www.ncbi.nlm.nih.gov/pmc/articles/PMC1446193/
15.	https://www.ncbi.nlm.nih.gov/pmc/articles/PMC6730615/	https://www.ncbi.nlm.nih.gov/pmc/articles/PMC4512265/
16.	https://www.ncbi.nlm.nih.gov/pmc/articles/PMC6352058/	
17.	https://www.ncbi.nlm.nih.gov/pubmed/23697503	https://www.ncbi.nlm.nih.gov/pubmed/22864067
18.	https://www.ncbi.nlm.nih.gov/pubmed/10968469	https://www.ncbi.nlm.nih.gov/pubmed/10866440
19.	https://www.ncbi.nlm.nih.gov/pubmed/10968470	
20.	https://www.sciencedirect.com/science/article/abs/pii/S0168851014003455	https://www.sciencedirect.com/science/article/abs/pii/S016885101500216X
21.	https://www.sciencedirect.com/science/article/pii/S0140673615012957	

22.	https://www.thelancet.com/journals/lancet/article/PIIS0140-6736(13)60116-6/fulltext	https://www.thelancet.com/journals/lancet/article/PIIS0140-6736(13)61024-7/fulltext
23.	http://www.cesr.org/sites/default/files/Health_and_Welfare_in_Iraq_after_the_Gulf_Crisis_1991.pdf	Found when searching for "Report on the changes in the nutritional status of Iraqi children: one year following the Gulf War and sustained sanctions".
24.	https://adc.bmj.com/content/88/1/92.1	Found when searching for "niazi al-kubaisi The humanitarian and health impact of war and embargo on Iraq".
25.	https://www.icrc.org/en/international-review/article/humanitarian-implications-wars-iraq	
26.	https://www.thelancet.com/journals/lancet/article/PIIS0140-6736%2803%2912716-X/fulltext	
27.	https://www.thelancet.com/pdfs/journals/lancet/PIIS0140-6736(03)12619-0.pdf	
28.	https://www.ncbi.nlm.nih.gov/pubmed/7798873	Found while searching for "AK Kirkpatrick Economic sanctions and health".
29.	https://www.ncbi.nlm.nih.gov/pubmed/7872589	
30.	https://www.ncbi.nlm.nih.gov/pmc/articles/PMC1227827/pdf/cmaj_157_3_281.pdf	
31.	https://www.sciencedirect.com/science/article/pii/S014067369607376X	
32.	https://www.sciencedirect.com/science/article/pii/S0140673605658849	
33.	https://www.karger.com/Article/Abstract/26161	
34.	https://www.econstor.eu/bitstream/10419/178615/1/ile-wp-2018-12.pdf	Found while searching for "carneiro Economic Sanctions, Political Survival, and Human Rights: An Empirical Investigation APSA".
35.	https://www.ncbi.nlm.nih.gov/pubmed/23729786	https://www.ncbi.nlm.nih.gov/pubmed/23179240

Table A1.3: inclusion of sources with multiple versions

unpublished version	published version
Bundervoet T, Verwimp P. Civil war and economic sanctions: an analysis of anthropometric outcomes in Burundi. Brighton:University of Sussex, 2005	Bundervoet T, Verwimp P, Akresh R. Health and civil war in rural Burundi. *Journal of Human Resources* 2009;44:536–63.
Garfield R. Morbidity and mortality among Iraqi children from 1990 to 1998: assessing the impact of economic sanctions. Notre Dame IN: Joan B. Kroc Institute for International Peace Studies and Fourth Freedom Forum, 1999.	Garfield R, Leu C. A multivariate method for estimating mortality rates among children under 5 years from health and social indicators in Iraq. *Int J Epidemiol* 2000;29:510–15.
Gutmann J, Neuenkirch M, Neumeier F. Sanctioned to death? The impact of economic sanctions on life expectancy and its gender gap, Trier: Trier University, 2018.	Gutmann J, Neuenkirch M, Neumeier F. Sanctioned to death? The impact of economic sanctions on life expectancy and its gender gap. *Journal of Development Studies* 2021;57:139–62.

Table A1.4: exceptions to inclusion criteria

reference	justification for inclusion
Garfield R. Suffer the innocents. *The Sciences* 1999;39:19–23.	No peer-review, but authoritative contributor
Garfield R. The public health impact of sanctions: contrasting responses of Iraq and Cuba. *Middle East Report* 2000;215:16–9.	No peer-review, but authoritative contributor

Pinna Pintor M, *et al. BMJ Global Health* 2023; 8:e010968. doi: 10.1136/bmjgh-2022-010968

Figure A1.1: PRISMA™ flowchart. GS=Google Scholar, P=PubMed. For each search system, numbers for searches for the first and second chronological periods are given separated by slash bars. For more information see Page and colleagues[2] and http://www.prisma-statement.org.

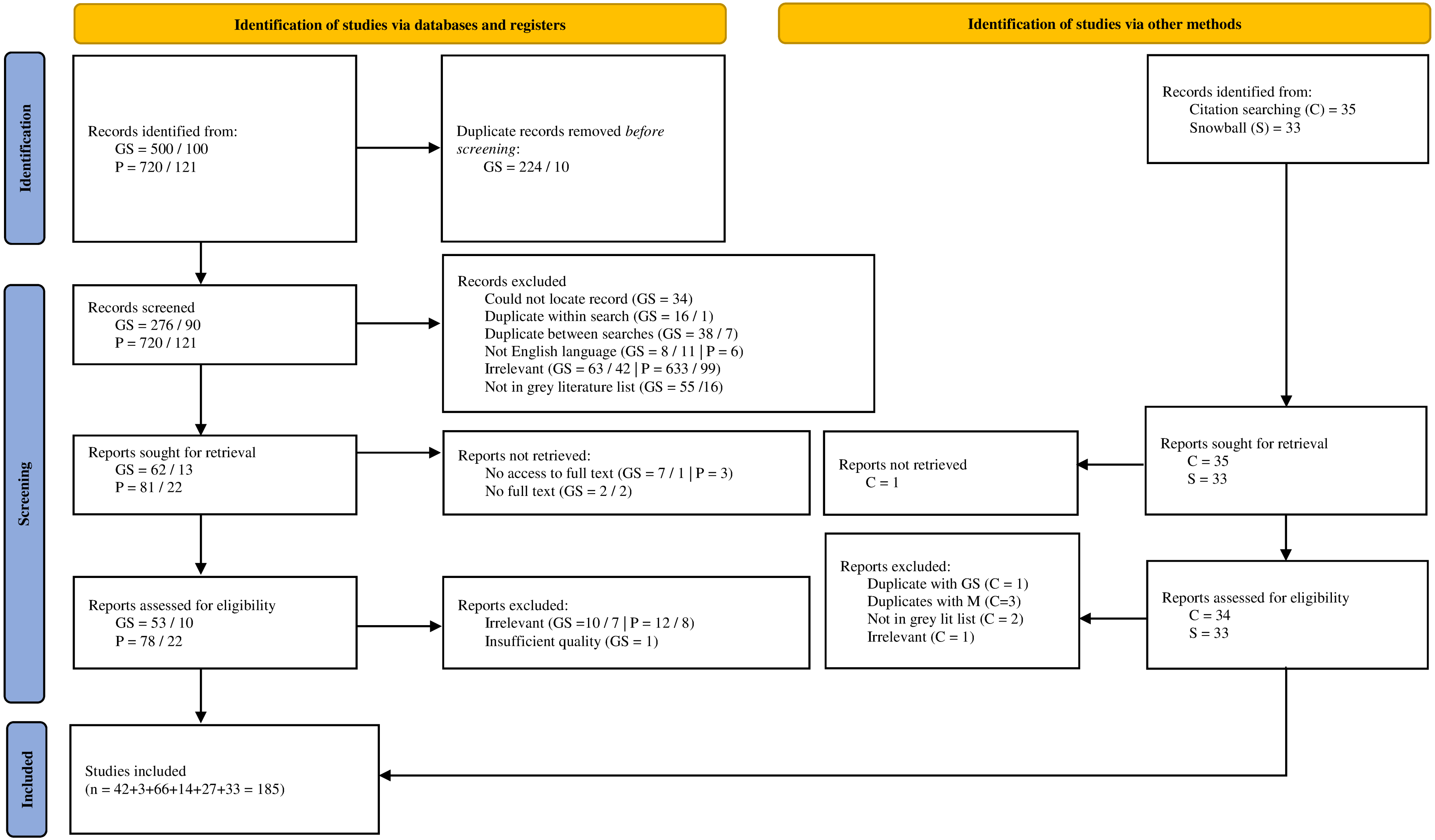

A2: Classification of studies by research method

Each source was classified according to its research method, applying the following definitions to the main approach adopted.

We designated as ORIGINAL RESEARCH a source which reports, discusses and manipulates observations to generate a novel assessment of the research question. We required the element of novelty, be it on the type of evidence provided or on the technique employed to manipulate it, to be clearly identifiable as separate from simple matters of opinion and personal judgment about existing findings. Original research was divided into studies adopting quantitative, qualitative and mixed-method approaches. We classified as MIXED-METHOD studies adopting both quantitative and qualitative techniques in a complementary fashion. Given the definition of *core* sources as necessarily including a quantitative element, all *core* studies using qualitative techniques are mixed-method studies.

We defined as QUALITATIVE sources reporting and discussing evidence elicited from interested parties by means of interviews, usually but not necessarily in person, and systematic observations and experiences made by the study author(s). To qualify as qualitative, we required sources to include a description of the context in which observations were made, a description of subjects encountered and interviewed, and details of field travel. We excluded memoires and unstructured reports claiming first-hand, in-person experience.

We defined as QUANTITATIVE sources entailing the manipulation of raw numerical data, coming from primary or secondary sources including collations of data from existing literature. We further divided them into DESCRIPTIVE studies, in which numerical summaries and sample statistics are used but no estimation or inference is performed, although logical inferences can be found in terms of informed judgments; UNCONTROLLED COMPARISON studies, which compare an outcome for two or more groups of study units known to differ in terms of an exposure of interest, without taking into consideration any other potential determinant of the outcome; CONTROLLED COMPARISON studies, which perform this comparison conditional on a series of chosen control variables, by means of techniques like regression, matching, stratification, standardization and adjustment of variables; QUASI-EXPERIMENTAL studies, which perform the controlled comparison by relying, with adequate justification and motivation, on one of the following techniques: regression discontinuity and interrupted time series; instrumental variables; difference studies (before-and-after studies, difference-in-difference and fixed effects estimators). We base our list on recent taxonomies,[3–5] but we exclude 'natural experiments', as the definition we use does not represent, or map to, a specific statistical technique. As emphasized by the literature, the designation of a quasi-experimental study must be partly based on a qualitative judgment about the pertinence of the employed technique and its assumptions. Some studies were not designated as quasi-experimental due to the perceived failure to satisfy this requirement.

We defined a quantitative study as providing an IMPACT ESTIMATE when it presented information relating variation in measures of health and health system performance to variation in exposure to sanctions. Some quantitative studies failed to satisfy this criterion because, for example, they compared an outcome at different point in times under sanctions, without any known or documented change in one or more variable aspect of sanctions; or because they performed analyses of outcomes immediately after sanctions were lifted, presenting interpretations related to sanctions but failing to report any observation under sanctions. In a small number of limit cases, an impact estimate could be inferred from information reported separately by different studies; or from a strong reason to assume a specific value for missing information – for example, a baseline value of zero for the occurrence of a condition never recorded before sanctions. These limit cases have been conservatively taken not to provide an impact estimate, and therefore excluded from the *core* sample.

We classified as REVIEWS sources reporting results of existing research to generate a comprehensive summary of current knowledge; SYSTEMATIC REVIEWS included details about the search strategy originating the list of references, and a pre-specified rule about the presentation, assessment and aggregation of findings from reviewed sources. We classified as COMMENTARIES sources discussing selective parts of the literature for various reasons (e.g. a comparative perspective, focus on a specific case) without generating comprehensive summaries of the previous literature or adding an original contribution, but reformulating existing evidence to support specific claims; and as EDITORIALS, OPINION PIECES AND CORRESPONDENCE brief expert opinions or exchanges of opinions about a given article or set of articles, with limited referencing and quoting of data.

Pinna Pintor M, *et al. BMJ Global Health* 2023; 8:e010968. doi: 10.1136/bmjgh-2022-010968

A3: Assessment of study quality and synthesis of effects

We reviewed the ROBINS-I tool[6] and discussed the applicability of its criteria to the core studies. For some of the tool's bias domains, we noted the following issues and associated conceptual solutions.

Confounding. The nature and current knowledge of the subject imply that the effect of interest has a very extensive definition: the impact of sanctions on population health and health systems in low-income and middle-income countries (LMICs). The range of study designs, outcomes, and effect measures consistent with this definition is large, and fails to identify a unique pre-specified list of key confounders. Retrieved studies are too few and scattered across these possibilities to enable synthesis for separate groups. As a solution, it was decided to assess confounders by iterative comparisons between all core studies, with additional information from non-core studies when relevant. Background information was also deemed insufficient to fully evaluate some implementation choices. For example, in designs with difference estimators, an unobserved factor may be a confounder if time-varying, or an effect modifier if fixed. However, information required to evaluate this choice was often unavailable. Current subject matter knowledge is also insufficient to fully evaluate specification choices in regression analyses, such as functional form, the re-scaling of variables and the construction of indices. We therefore concluded that information about confounding so conjectural ought not to be given a scalar score, and is best left in verbal form.

Bias in measurement classification of interventions. As sanctions are neither interventions nor prospectively monitored, and are instead approximately external exposures, incentives and opportunities for self-selection are limited. Therefore, retrospective ascertainment of exposure status *per se* is a poor indicator of the risk of misclassification. Instead, this must be evaluated by assessing the quality of the information used and whether robustness checks or placebo tests are implemented to investigate the sensitivity of results to alternative definitions of exposure. We decided to evaluate studies by focusing on these aspects, on which the tool provides little guidance.

Bias due to deviations from intended interventions. The nature and current knowledge of the subject suggests that no intended or typical experience of sanctions can be identified. As the effect of interest is presumed to be harmful, there is no intended course of action conditional on exposure. Hence, notions related to compliance (e.g. adherence, implementation failure, etc.) have no meaningful analogue in this setting. A typical course of events under the exposure might exist, but cannot be defined with current background knowledge. Absent those pre-specifiable benchmarks, we assess suggestions and analyses for all proposed effect size modifications and mediators, regarding them as relevant components of the effect of interest.

Bias in selection of the reported result. The lack of a clear benchmark for determining the list of confounders and the operationalization of estimates complicates the assessment of reporting, as there might be no way to adjudicate between multiple, equally plausible analysis plans. This makes it difficult to demarcate between, for example, exploratory specification searches and more arbitrary selection of hypotheses to test and report. An overall judgment at the literature level was deemed more appropriate, while data integrity was found to be an important issue omitted by the tool, and included in this domain.

As an alternative, a semi-structured qualitative risk-of-bias assessment was developed. The taxonomy of bias domains of the ROBINS-I tool[7] was used to develop a simple checklist aimed at detecting 'bias concerns' in core studies (Table A3.4). Bias sub-domains were developed by iteration and consensus among reviewers, aiming at capturing the most significant aspects of quality in each study. For each effect reviewed of each study, a qualitative judgment was made about whether any of the analyses raised any of the specified problem. An inclusive approach to the effects included in the assessment was followed, as almost no study presented a clear demarcation between primary and secondary outcomes and analyses, and as the main aim of the synthesis is to test for the existence of effects on all health and health system outcomes. However, concerns were not attributed when originating from analyses presented to illustrate an acknowledged problem, preliminary to the presentation of further analyses devised in order to address it. Analyses of effect size modification and mediation performed after a main analysis were only assessed in terms of the modified bias domain referring to them. An additional category was introduced to represent concerns with external validity and data integrity issues.

For each study i we develop the overall quality score

$$\mathbf{Q}_i = 7 - n_i$$

Where n_i is the number of bias domains in which concerns are identified (excluding the added domain). We do not compute this statistic for studies with data integrity concerns.

To synthesize impact estimates through vote counting, we construct a standardized metric of effect direction. For each study i, we assign to each reported effect δ a score D taking on two possible values:

$$D_\delta = \begin{cases} 1 & if\ \delta\ harmful \\ -1 & if\ \delta\ beneficial \end{cases}$$

Where ‘harmful’ denotes an adverse impact on health or health system performance, with a sign depending on the nature of the outcome. We exclude effects for auxiliary analyses of modification or mediation. In each study, effect scores were merged into a ‘synthesized effect’ based on considerations of homogeneity and complementarity, such as when analyses were presented for a combination of mutually exclusive groups that collectively summed up to (or nearly so) the relevant population (e.g. bilateral and multilateral sanctions, men and women, infant and 1-4 mortality for under-5 mortality). Hence, for each study i and set of synthesizable effects S, the resulting indicator takes on three possible values:

$$D_{S_i} = \begin{cases} 1 & if\ D_\delta = 1\ \forall \delta \in S_i \\ 0 & if\ \exists \delta' \neq \delta'' |\ D_{\delta'} \neq D_{\delta''}\ \delta', \delta'' \in S_i \\ -1 & if\ D_\delta = -1\ \forall \delta \in S_i \end{cases}$$

where zero denotes conflicting evidence. For effects without homogeneous counterparts $D_\delta = D_{S_i}$. The same aggregation rule was repeated to obtain an overall study score D_i that was either equal to the scores for synthesized effects in case these were identical, or coded as ‘conflicting evidence’ if otherwise.

$$D_i = \begin{cases} 1 & if\ D_{S_i} = 1\ \forall\ S_i \\ 0 & if\ \exists S_i' \neq S_i'' |\ D_{S_i'} \neq D_{S_i''} \\ -1 & if\ D_{S_i} = -1\ \forall\ S_i \end{cases}$$

This conservative aggregation rule safeguards against false positives in studies performing many analyses, and avoids the frequent arbitrariness and poor performance validity of more complex algorithms.[8] In keeping with best practice, we do not consider statistical significance as a criterion in any of these steps.

Overall scores visualized with oriented triangles (▲, ▼, ◄►) are presented in an effect direction plot[9] (Table 1) and incorporated into a more extensive presentation (Table A5.1). Univariate and bivariate harvest plots[10] are used to visualize the distribution of quality and direction-of-effect scores. Stratification for outcome domains did not uncover relevant patterns, and is thus omitted.

Binomial probability tests for the proportion of studies reporting an overall adverse effect were carried out using the -bitest- command in Stata 17.0 MP[11] (Table A3.1). We tested one-sided hypotheses towards an excess of studies reporting adverse effects because we assume that studies are designed to test for adverse effects; and because it has been noted that, in the presence of heterogeneous effects and sample sizes, the power of the test is not monotonically increasing in the proportion of favorable cases, leading to inconsistent results for two-sided hypotheses.[12] The command -twoway tabulate- with the -exact- option was used to compute Fisher’s exact test for whether the proportion of studies reporting an overall adverse effect was different between early and recent studies, and between studies of early and recent sanction episodes (Table A3.2). The command -ttest- was used to compute Student’s t test for differences in the overall quality score between studies reporting an overall adverse effect of sanctions and all other studies, early and recent studies, and studies of early and recent sanction episodes, allowing for unequal group variances (Table A3.3).

Table A3.1: one-sided binomial probability tests for excess detection of effects in core studies. (a) observed number of studies reporting consistent adverse effects; (b) expected number of studies reporting consistent adverse effects under the hypothesis of no adverse effect and a probability of false positive π. **p <.05 ***p<.01

	N	π	a	b	(a) - (b)
**1**	27	0.5	21	13.5	7.5***
**2**	27	0.6	21	16.02	4.98**

Table A3.2: Fisher's exact test for association between direction of effect and publication or sanction episode period in core studies. Cross-country studies omitted from the analysis of differences in direction of effect by sanction episode periods. (†) Cross-country studies omitted.

		no harm	harm	p value
**publication period**	**Early (1992-2005)**	2	8	1.00
	**Recent (2006-2019)**	4	13	
**sanction episode period†**	**Early (before 2010)**	3	11	1.00
	**Recent (in/after 2010)**	1	6	

Table A3.3: two-sided t tests for differences in quality by direction of effect, publication period, and sanction episode period in core studies. (a) average overall quality score for (in row order): studies not reporting consistent adverse effects, early studies, studies of early sanction episodes (as in Tab A3.2); (b) average overall quality score for: studies reporting consistent adverse effects, recent studies, studies of recent sanction episodes. (†) Cross-country studies omitted. **p <.05

	N	a	b	(b) - (a)
**direction of effect**	27	4	4.42	0.42
**publication period**	27	4.2	4.41	0.21
**sanction episode period†**	21	4.14	5.14	1**

Table A3.4: bias domains and subdomains for semi-structured risk-of-bias comments. (†) cross-country studies only.

Confounding	Model choice	Does the study contain an acceptable discussion of the analytic method employed and the choice of variables (eg regression specification)?
	Model sensitivity	Does the study perform adequate sensitivity analyses to test if results change after motivated changes in analytic method (eg regression specifications, functional forms, transformation of key variables)?
	Omitted control	Has any factor likely to affect the outcome and the probability of exposure to sanctions been omitted from the analysis? Were the factors included in the adjustment procedure adequately measured and operationalized? Was the adjustment procedure correctly implemented?
	Bad control	Is any of the variables used to adjust the estimated impact of exposure to sanctions on the outcome likely to have been affected by exposure to sanctions?
Selection	Registry reporting	If the study uses administrative data, is the probability of reporting information to the administrative body likely to be affected by exposure to sanctions (or one of its causes) and by the outcome (or one of its causes)?
	Facility enrolment	If the study uses facility data, is the probability of enrolment into (or use of) the facility likely to be affected by exposure to sanctions (or one of its causes) and by the outcome (or one of its causes)?
	Outmigration	If the study uses survey data, is the probability of leaving the survey area before being interviewed likely to be affected by exposure to sanctions (or one of its causes) and by the outcome (or one of its causes)?
	Mortality	Is the probability of dying and therefore being excluded from the study sample likely to be affected by exposure to sanctions (or one of its causes) and by the outcome (or one of its causes)?
Error in exposure	Limited information	Is the information used to define groups or variables representing exposure to sanctions reliable and consistent? Does the study provide a sufficient description of the sanction episode or dataset?
	Unclear definition	Is the definition of groups or variables representing exposure to sanctions sufficiently precise in terms of timing and geographical coverage to assess the risk of incorrect classification of exposure status?
	Definition sensitivity	Is there any attempt to test the sensitivity of results to small changes in the definition of groups or variables representing exposure to sanctions?
Modification/mediation	Omitted modifier/mediator	If analyses were performed to identify factors mediating or modifying the impact of sanctions, was any factor likely to exert this role omitted?
	Biased modifier/mediator	If analyses were performed to identify factors mediating or modifying the impact of sanctions, were analytic methods adequate? Could interactions be given a causal interpretation?
Missing data	Sparse data	Are outcomes and additional variables observed only for a small subset of the study period?
	Complete cases only	Are observations with missing information for any of the variables employed in the study excluded from the analysis? If so, is exclusion likely to be associated to exposure to sanctions?
	Imputation issues	Is the method used to impute missing data clearly described, and results adequately discussed?
Error in outcome	Definition comparability	Is the definition of the outcome changing across time or units of observation? Is any change in definitions related to exposure to sanctions?
	Definition sensitivity	Is there any attempt to test the sensitivity of results to motivated changes to the definition of the outcome (e.g. log-transformations, outcome placebo test)?
	Self-reports only	Were all outcome variables based on information reported by study subjects or other individuals?
Reporting of results	Graph only	Is any of the study analyses discussed verbally but reported only in graphical form?
	Not shown	Is any of the study analyses discussed verbally but reported in neither table nor graphical form?
	Data integrity	Is any of the study analyses based on data from the FAO/NRI or ICMMS surveys?
Other	Unclear population	Is the relation between the study sample and the population it comes from clarified? Does the study provide sufficient information to understand to whom the results should apply, other than to the sampled units?
	No error clustering	If the study uses survey data, are standard errors of sample statistics and estimates clustered at the level of survey cluster to account for the design effect?
	Old sampling frame	If the study uses survey data, is the survey's sampling frame likely to accurately track the distribution of the study population at the time of the survey?
	Quoted data integrity	Does any quoted material originate from studies based on the FAO/NRI or ICMMS surveys?
	Omitted episodes†	Is any of the following sanction episodes missing from the dataset? Iraq (1990-2003), Serbia-Montenegro (1991-2001), Haiti (1991-1994), Cuba (1992-ongoing), Iran (various post-2010)

Pinna Pintor M, *et al. BMJ Global Health* 2023; 8:e010968. doi: 10.1136/bmjgh-2022-010968

A4: Case study. The dynamic impact of sanctions in Iraq

The largest research effort to track the impact of sanctions across time has been made in the case of Iraq (1990-2003). Early findings focused on the first four months of sanctions, before military operations, and the first eight months of 1991, which include the period of active armed conflict. These show a several-fold increase in mortality risk among children of various age classes, relative to selected comparison periods.[13,14] The nationally representative survey on which these findings are based is not free from concerns. Recall bias in children's year of death, and possibly in deaths themselves, may have biased upward the estimated rate ratios – which come from a complete-case analysis. Bounds to these estimates based on assumptions as to the extent of recall bias were not provided. On the other hand, some of the mortality risk due to war and sanctions faced by children might have also impacted their mothers, and the resulting 'survivor bias' might have biased estimates downward. Overall, the ability to replicate expected patterns of heterogeneity (by age, maternal education and rural vs. urban location) supports the quality of the survey. Findings are also consistent with qualitative evidence.[15,16]

Substantial uncertainty and controversy has surrounded assessments of the evolution of these effects throughout the entire episode, first and foremost due to the lack of reliable, routinely collected vital statistics. After 1991, the only two surveys of child mortality implemented before the end of sanctions in May 2003 cannot be deemed reliable.

The first[17] was carried out in 1995, sponsored by FAO and the Nutritional Research Institute of the Iraqi Ministry of Health (NRI), and followed a subset of the clusters sampled by the nationally representative study of Ascherio and colleagues[14] in Baghdad. Based on complete birth histories going back to 5 years before the imposition of sanctions, under-5 mortality was found to have increased from 40.6 per 1000 live births in this baseline period to 198.2 in the subsequent 5 years. This nearly five-fold increase (RR 4.88; 95% CI:3.43−6.94) thus appeared to suggest further deterioration from the estimate of Ascherio and colleagues[14] – in which the sanction period for this age band was truncated by the survey date. The study also found that the prevalence of undernutrition increased from 12% to 28% for stunting, from 7% to 29% for underweight, and from 3% to 12% for wasting. However, the mortality findings were later revised,[18] as a second follow-up in 1996 showed no increase in child mortality (38 per 1000) and a large mismatch in reported deaths – mostly deaths reported only in the first follow-up. Investigation led to only partial reconciliation, and uncovered misclassification of miscarriages and stillbirths as infant deaths. Zaidi, the leading investigator, conjectured that "an accurate estimate of child mortality in Iraq probably lies between the two surveys".[18] Various authors have pointed out that in the first follow-up interviewers were government-appointed and oversight by international team members was limited, suggesting that the data was vulnerable to manipulation.[19,20] Spagat reports a personal communication by Zaidi supporting these suspicions.[20]

The second survey implicated in charges of fraud is the Iraq Child and Maternal Mortality Survey (ICMMS), carried out in 1999 by UNICEF in collaboration with the Iraqi central government and the Kurdish autonomous region, leading to two separate survey branches for the Centre/South and North of the country. The survey showed a very large increase in infant and under-5 mortality in the Centre/South region, from 47 and 56 in 1984-1989 to 108 and 131 in 1994-1999. The respective figures for the Northern region show continued – if slower – secular decline from 64 and 80 to 59 and 72 in the same periods, with only temporary upward fluctuation in between.[21] Concerns over the reliability of the Centre/South branch of the survey were first raised in the context of an independent assessment of the Oil-for-Food Programme established by UN Secretary General Kofi Annan. The expert panel observed that most of the surge in child mortality was abnormally concentrated in the first year of life and could not be found in sources deemed more reliable, such as Iraq's 1997 population census, concluding that this part of the survey "could conceivably have been tampered with".[22] The ICMMS was defended by considering possible infant-specific causes of death related to Iraq's exceptional circumstances and possible reconciliation with census data.[23] Dyson[24] speculated that the lower estimates from the ICLS and 1997 census reflected a surge in under-reporting of deaths under sanctions, as food rationing encouraged households to retain access to their dead children's rations – although the presumed exemption of the ICMMS from this problem was left unexplained. However, after later surveys replicated the pattern depicted by ILCS data, he concluded that manipulation of the ICMMS dataset must have occurred.[25,26]

However, uncertainty remains even after deprecating this data. Under sanctions, information on child nutritional status was collected by a number of cross-sectional surveys.[27] Using some of this data and other available indicators, Garfield and Leu[19] fitted logit models of under-5 mortality for a cross-section of countries to predict the rate in 1996 Iraq. Their preferred specification includes adult literacy, stunting prevalence, and the share of population with access to potable water as covariates, and yields a rate of 87 (95% CI: 80−95). Importantly, the model performs reasonably well in replicating the estimates of Ascherio and colleagues,[14] as well as expected patterns of regional variation. The exercise is limited by uncertainty over the model chosen, which may not fully capture changes in the mortality-undernutrition link in Iraq. These results suggest a scenario in which, after an acute mortality crisis in the first year of sanctions, child mortality stabilized downward, but nonetheless substantially above the level prevailing in the immediate pre-sanction period.

A different picture emerges from surveys implemented after the 2003 US invasion, suggesting that child mortality did not rise sharply under sanctions, and instead fluctuated around pre-sanction levels throughout the entire episode (Figure A4.1). Some of these surveys suffer from acknowledged weaknesses. In the Iraq Living Conditions Survey (ILCS), which generated the lowest mortality profile, the administration of the birth history module had to be repeated after a sub-sample check uncovered under-reporting of births and deaths.[28] This might have been due to respondent fatigue, as the median interview length was reported to be 83 minutes.[28] As a comparison, average lengths for long DHS questionnaires in South Africa (2016), Kenya (2014) and India (2015-16) were 32, 60 and 38 minutes respectively.[29] Whether the problem was addressed satisfactorily is unclear, and some judged the final figures as too low.[30,22] The use of these surveys to assess the dynamic impact of sanctions is further complicated by the long recall period involved, which might distort trends and mute short-term fluctuations. In this respect, it is not clear whether these findings question those of Ascherio and colleagues[14] or *vice versa*. In this second scenario the burden attributable to sanctions might amount to a decade-long interruption of mortality decline. As put by the ILCS final report, “the steady decline in child mortality rates in Iraq in the 1970s and 1980s was sharply interrupted at the time of the Gulf War in 1991”.[28]

Two interpretive points should be mentioned. First, nothing suggests the ruling out of intermediate scenarios. An early, large increase in mortality could have been driven by self-limiting and temporary factors, such as the presence of high-risk cohorts of children used to adequate pre-natal nutrition and the breakdown of immunization coverage[31] in the early 1990s. As immunization was re-established and later cohorts experienced undernutrition since conception, mortality due to undernutrition might have stabilized at a relatively lower level. This possibility underscores the need to go beyond an exclusive focus on mortality, as the high levels of non-fatal undernutrition documented under sanctions ought to be taken into account.[27] Second, in light of heated debate around child mortality figures, it is perhaps worth stressing that any adjudicated scenario would still require interpretation. In long sanctions episodes like that of Iraq, the confounding effect of short-term correlated shocks might be presumed to eventually fade away, but complexity is added by unfolding societal responses. Hence, descriptive long-term trends such as those in Figure A4.1, even if free from bias of practical significance, can only be attributed to sanctions in the literal and counterfactual sense which includes such responses – notably, government policies. For Iraq, Garfield[32, 31] has made a forceful case that a combination of pre-existing institutional weaknesses and perverse government decisions aggravated the situation.

Figure A4.1: under-5 mortality (deaths per 1000 live births) in Iraq, 1960-2010. (†) model-based prediction; (*) based on summary birth histories, one or two most recent observations omitted from series due to known design bias (for a discussion, see page 230 in the handbook of Preston and colleagues[33]). Sources: Iraq Fertility Survey 1974 (IFS), Demographic Sample Survey and Sample Registration System, 1973-4 (DSS/SRS), 1987 Census, Immunization, Diarrhoeal Disease, Maternal and Childhood Mortality Survey 1990 (EPI/CDD)[34]; International Study Team survey (IST)[14]; Multiple Indicator Cluster Survey 1996 (MICS 2)[19]; Iraq Living Conditions Survey (ILCS), Multiple Indicator Cluster Survey 2006 (MICS 3)[25]; Iraq Family Health Survey (IFHS)[28]; Multiple Indicator Cluster Survey 2011 (MICS 4): Tim Dyson, personal communication.

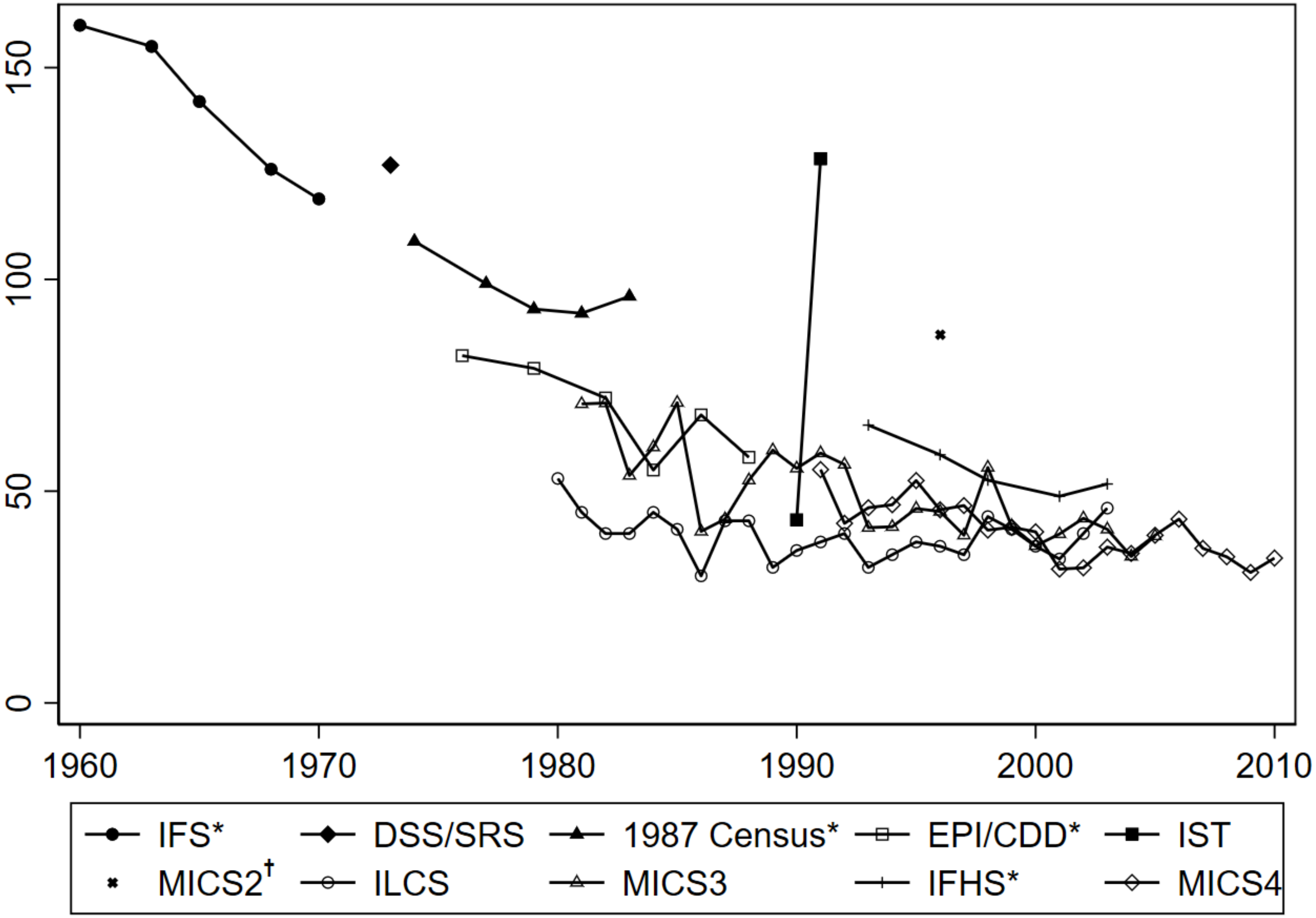

A5: Additional graphs and tables

Figure A5.1: timeline of financial sanctions in LMICs, 1950-2019. Source: Global Sanctions Database (GSDB) [2] V. 2, available at GSDB@drexel-edu. The following target aggregates were split into component countries: Balkans, Western Countries, Economic Community of West African States (ECOWAS), International Criminal Court (ICC) Rome Statute Signatories, League of Arab States, European Economic Community (EEC), European Union (EU), Council for Mutual Economic Assistance (COMECON). Episodes involving the Union of Soviet Socialist Republics (USSR) and Yugoslavia were respectively recoded to post-Soviet states, and both Serbia and Montenegro after 1991. Countries were included if classified as ‘Low income’ or ‘Lower middle income’ during their entire available period. In addition, countries were included limited to episodes initiated in a year when they were so classified, or using the closest year available; or if they were so classified for at least half of the available years of the episode. The classification used is the World Bank Analytical Classifications 2019. Zimbabwe refers to Rhodesia before 1979, Vietnam refers to North Vietnam before 1975. Cold War-related sanctions include sanctions against members of COMECON not imposed by the Soviet Union; sanctions imposed by the Soviet Union against countries not members of COMECON; sanctions imposed by the CoCom and ChinCom organizations; and exclude North Vietnam and Cuba before COMECON membership, sanctions against Cambodia, China, North Korea, Yugoslavia; and sanctions against USSR from neutral European states (Austria, Finland, Sweden and Switzerland). Episodes started and ended in the same year were coded to end in the subsequent year to allow visualization. LMICs, low-income and middle-income countries.

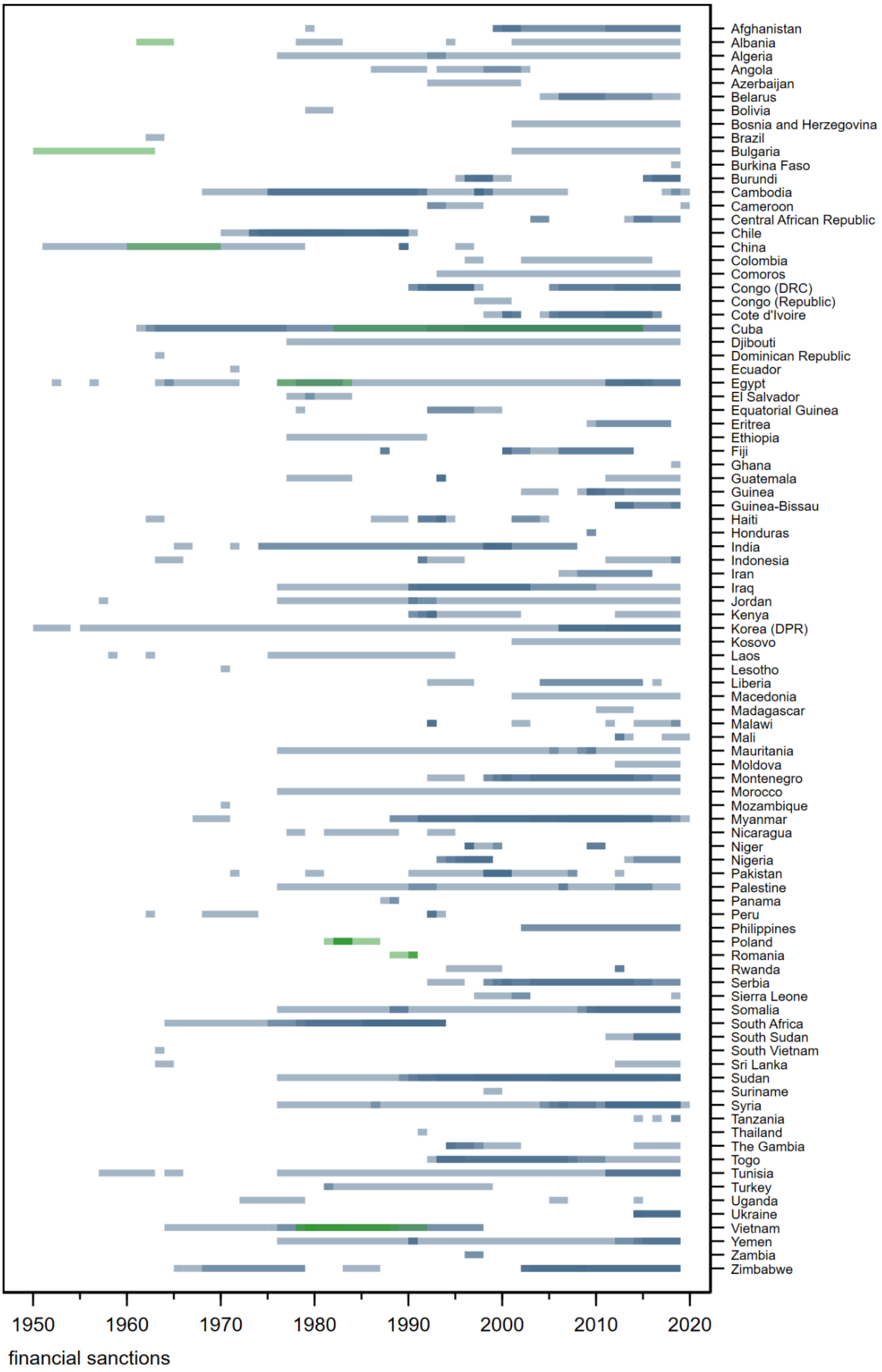
Afghanistan
Albania
Algeria
Angola
Azerbaijan
Belarus
Bolivia
Bosnia and Herzegovina
Brazil
Bulgaria
Burkina Faso
Burundi
Cambodia
Cameroon
Central African Republic
Chile
China
Colombia
Comoros
Congo (DRC)
Congo (Republic)
Cote d'Ivoire
Cuba
Djibouti
Dominican Republic
Ecuador
Egypt
El Salvador
Equatorial Guinea
Eritrea
Ethiopia
Fiji
Ghana
Guatemala
Guinea
Guinea-Bissau
Haiti
Honduras
India
Indonesia
Iran
Iraq
Jordan
Kenya
Korea (DPR)
Kosovo
Laos
Lesotho
Liberia
Macedonia
Madagascar
Malawi
Mali
Mauritania
Moldova
Montenegro
Morocco
Mozambique
Myanmar
Nicaragua
Niger
Nigeria
Pakistan
Palestine
Panama
Peru
Philippines
Poland
Romania
Rwanda
Serbia
Sierra Leone
Somalia
South Africa
South Sudan
South Vietnam
Sri Lanka
Sudan
Suriname
Syria
Tanzania
Thailand
The Gambia
Togo
Tunisia
Turkey
Uganda
Ukraine
Vietnam
Yemen
Zambia
Zimbabwe
1950
1960
1970
1980
1990
2000
2010
2020
financial sanctions

Figure A5.2: timeline of arms embargoes in LMICs, 1950-2019. See Fig. A5.1 for notes.

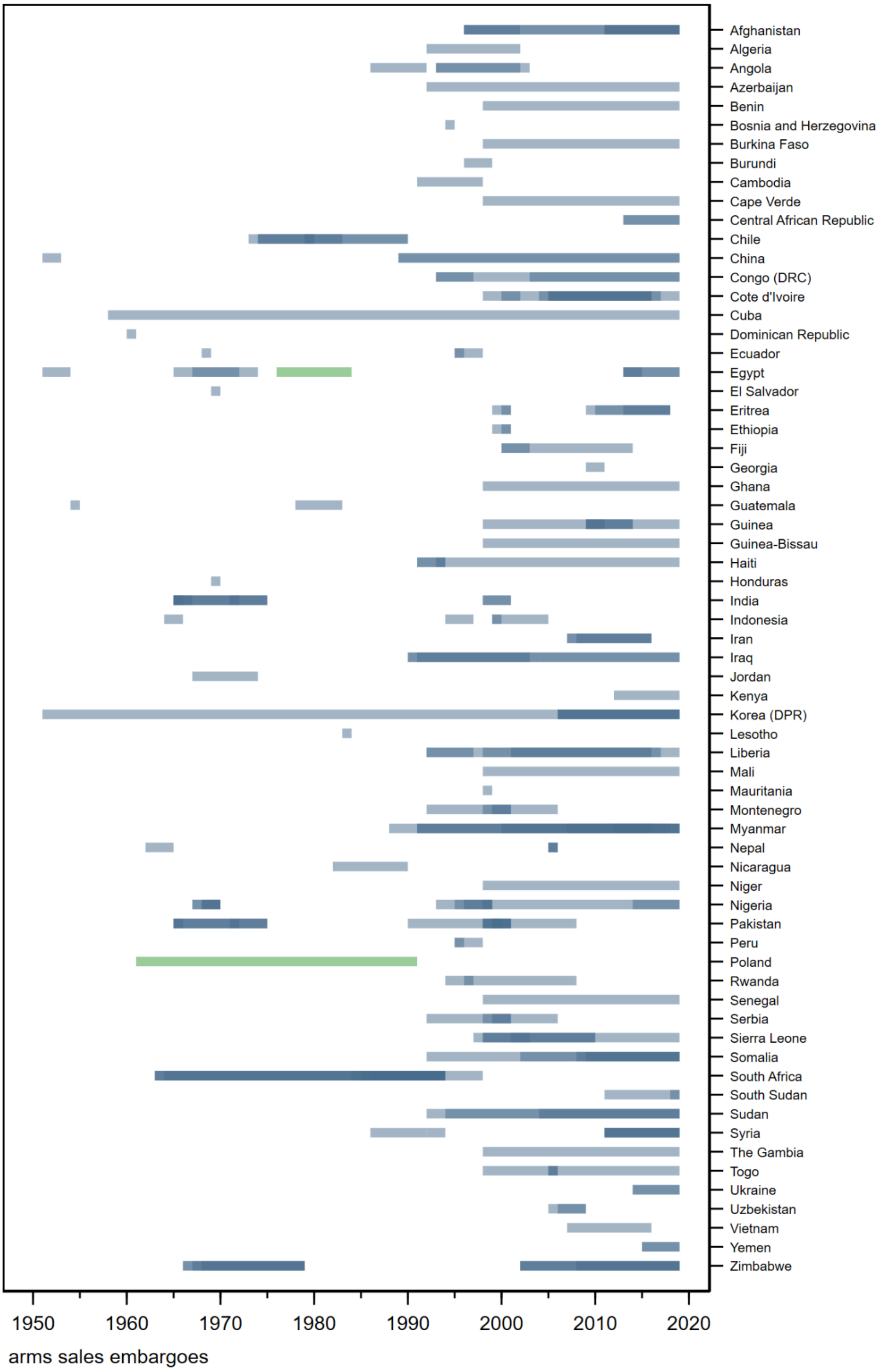

Figure A5.3: timeline of limitations to military assistance in LMICs, 1950-2019. See Fig. A5.1 for notes.

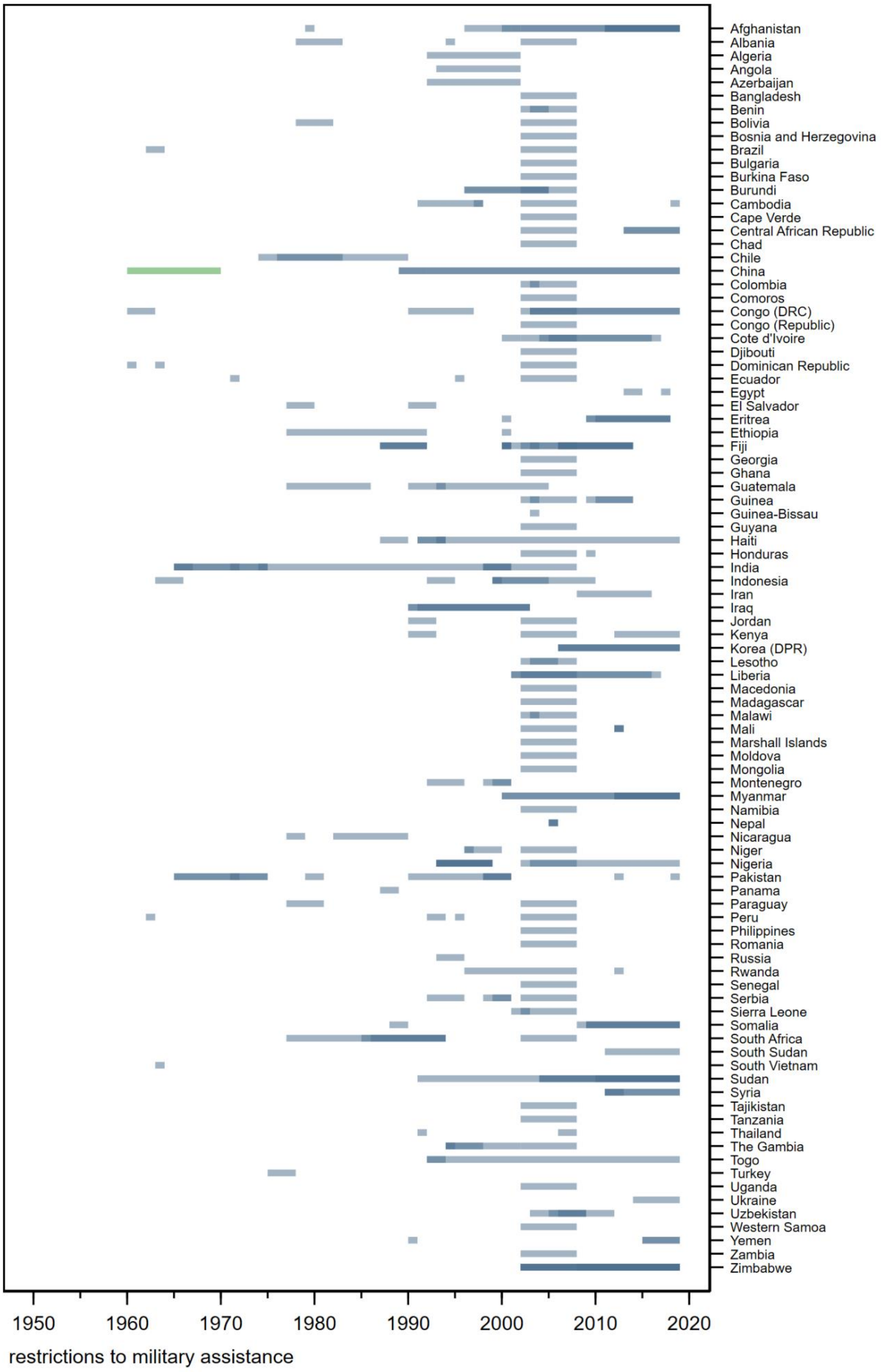

Figure A5.4: timeline of travel bans in LMICs, 1950-2019. See Fig. A5.1 for notes.

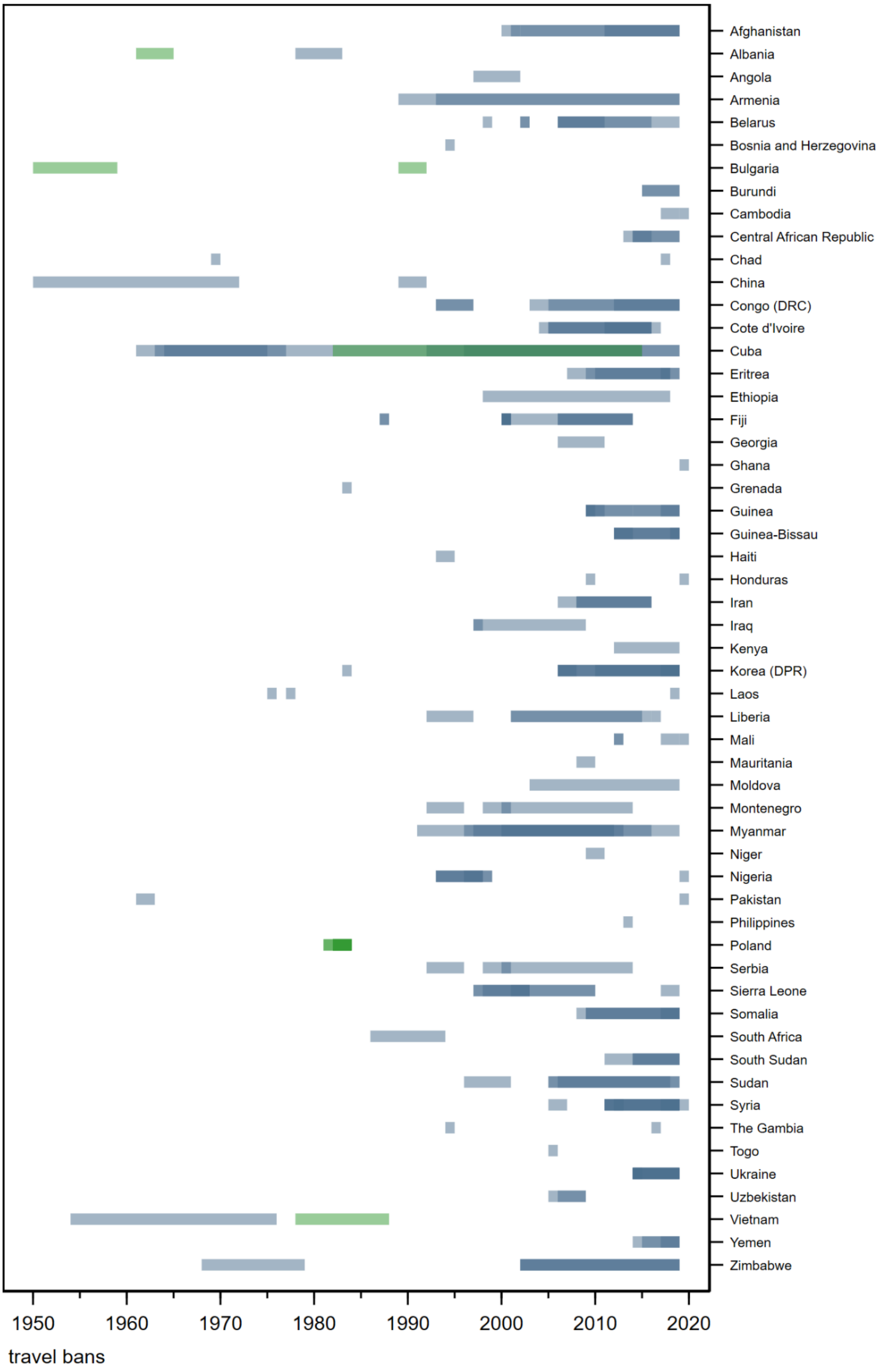

Figure A5.5: timeline of other types of sanctions in LMICs, 1950-2019. See Fig. A5.1 for notes.

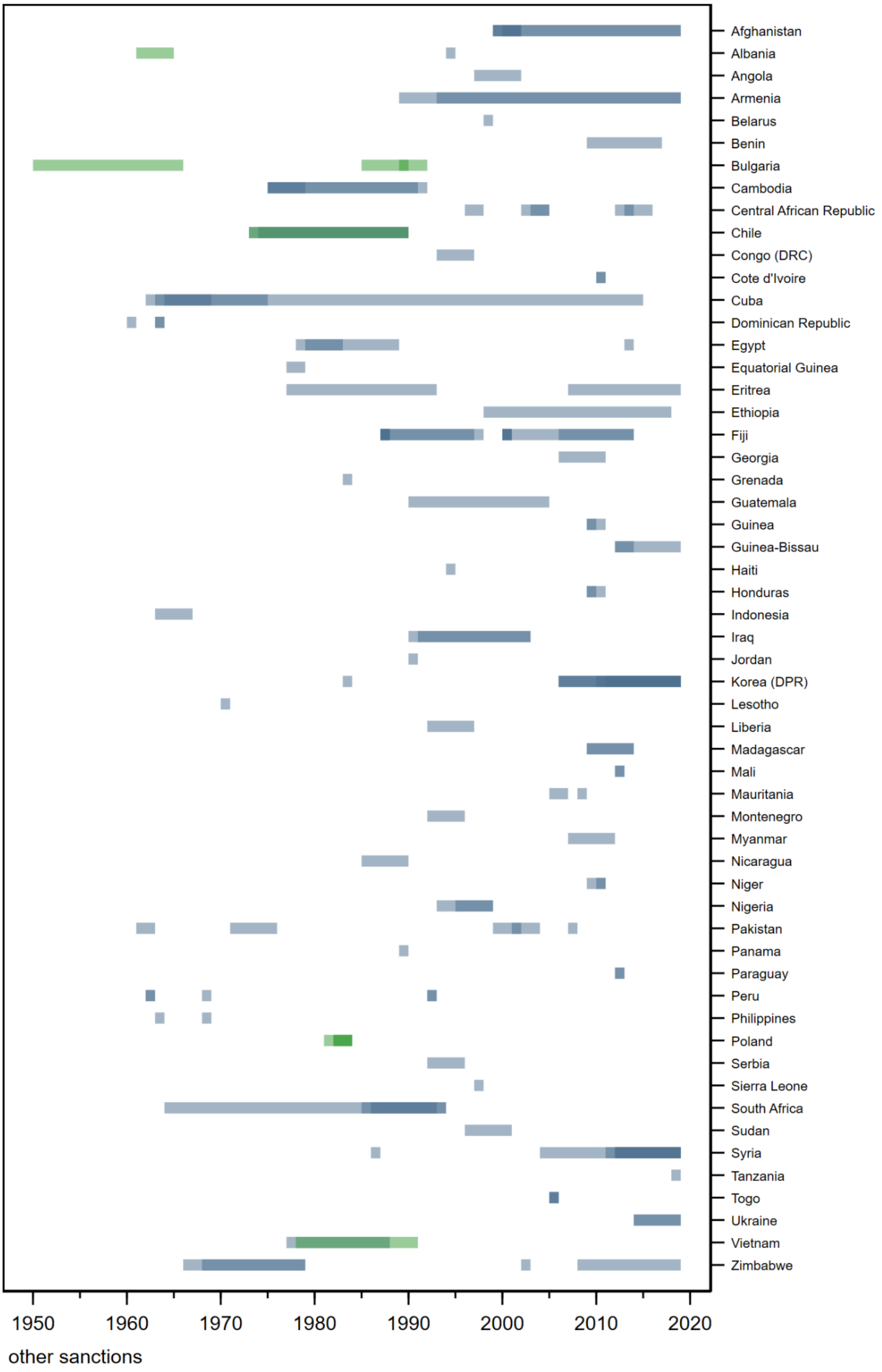

Figure A5.6: included sources by type of contribution and geographical focus. Red: core studies; green: non-core studies. Studies focusing on multiple selected countries (as opposed to cross-country studies) are observed repeatedly; 'general' includes studies without appreciable geographical focus.

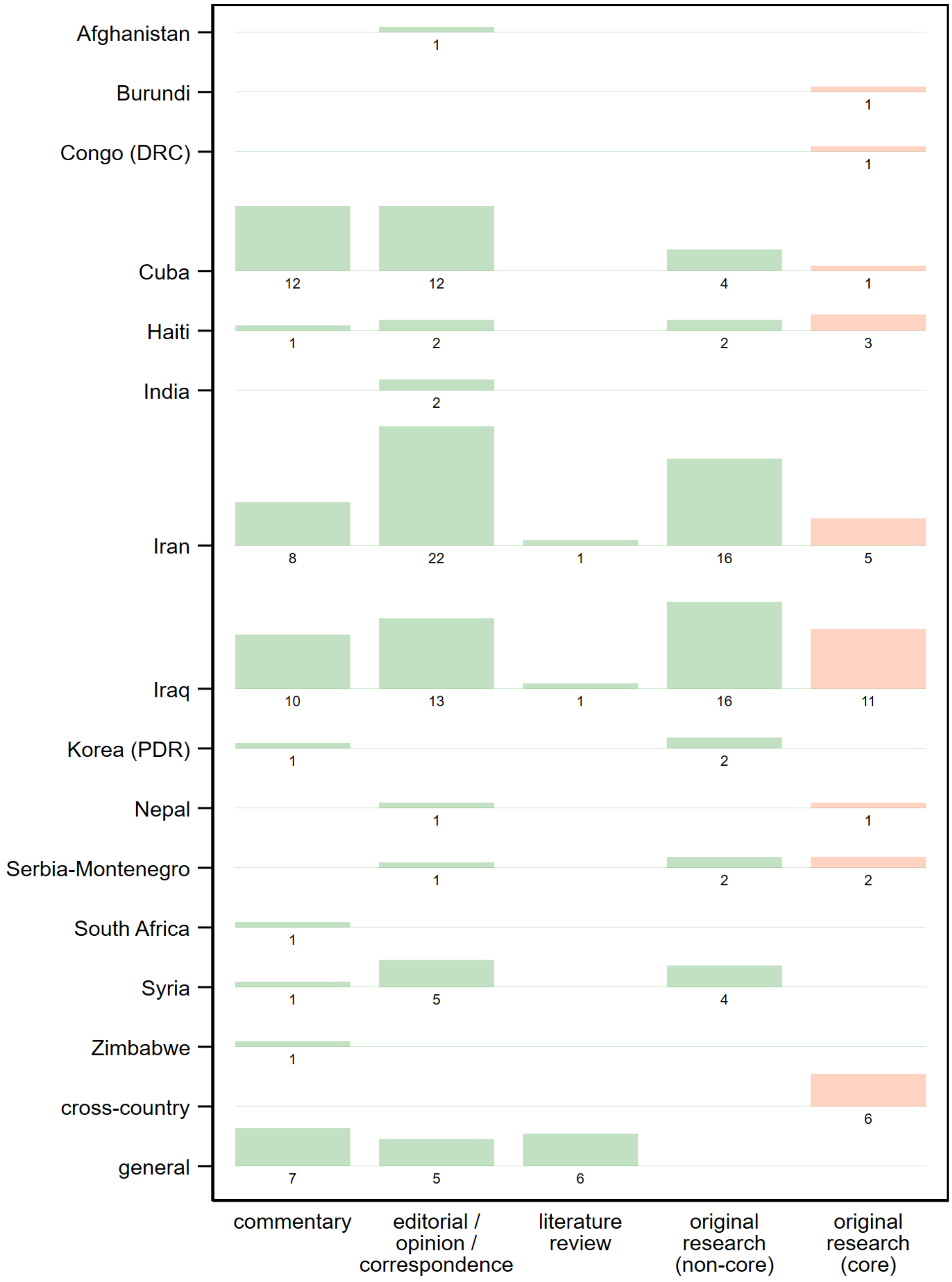

Figure A5.7: risk-of-bias concerns in core studies, by bias domain and. For definitions of bias domains and sub-domains see Table A3.4.

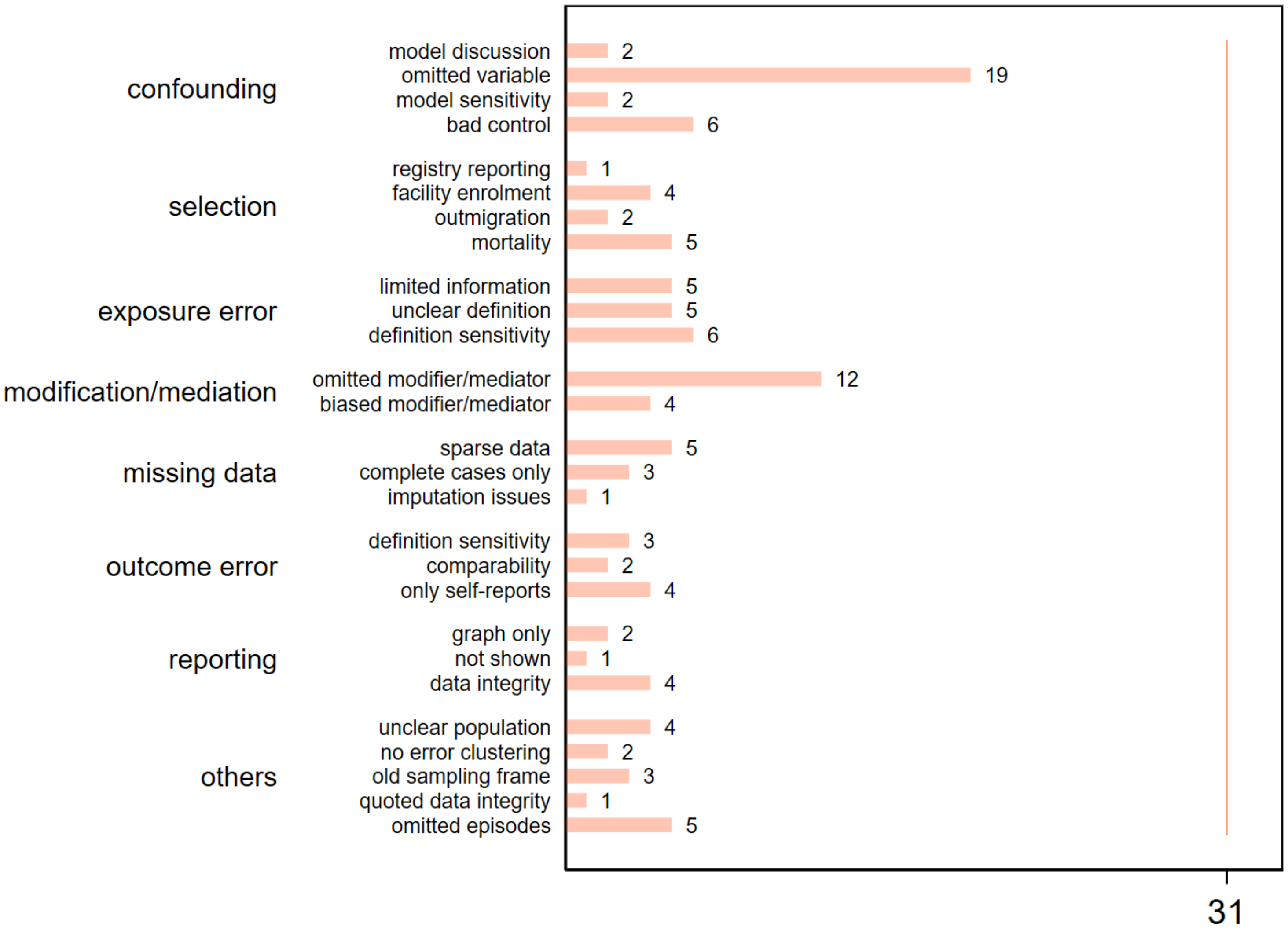

Figure A5.8: correlated shocks in core studies. Full dots denote studies not addressing plausible confounding due to correlated shocks, including confounding arising only under specific assumptions about time-varying structure and lagged effects. Empty dots indicate studies with an explicit strategy to control for such confounding, including the presence of additional control observations plausibly unaffected by the shock. For references see Table A5.1.

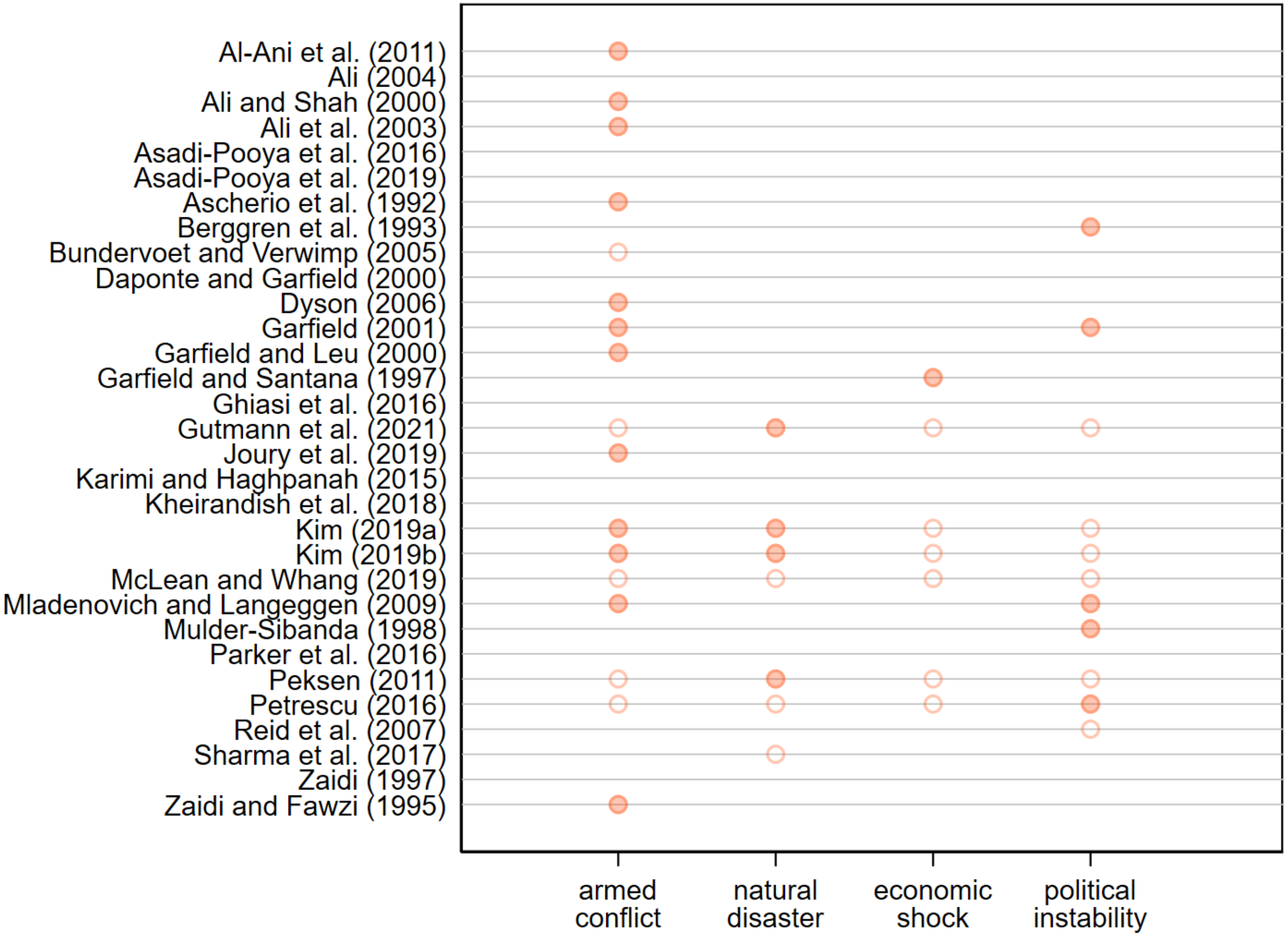

Figure A5.9: harvest plot of overall direction-of-effect scores in core studies, adjusted by overall quality score. Arrows denote adverse effect (↑), beneficial effect (↓), and conflicting evidence (↔). Details on the construction of the scores above, pp 7–10. Four studies with data integrity issues omitted.

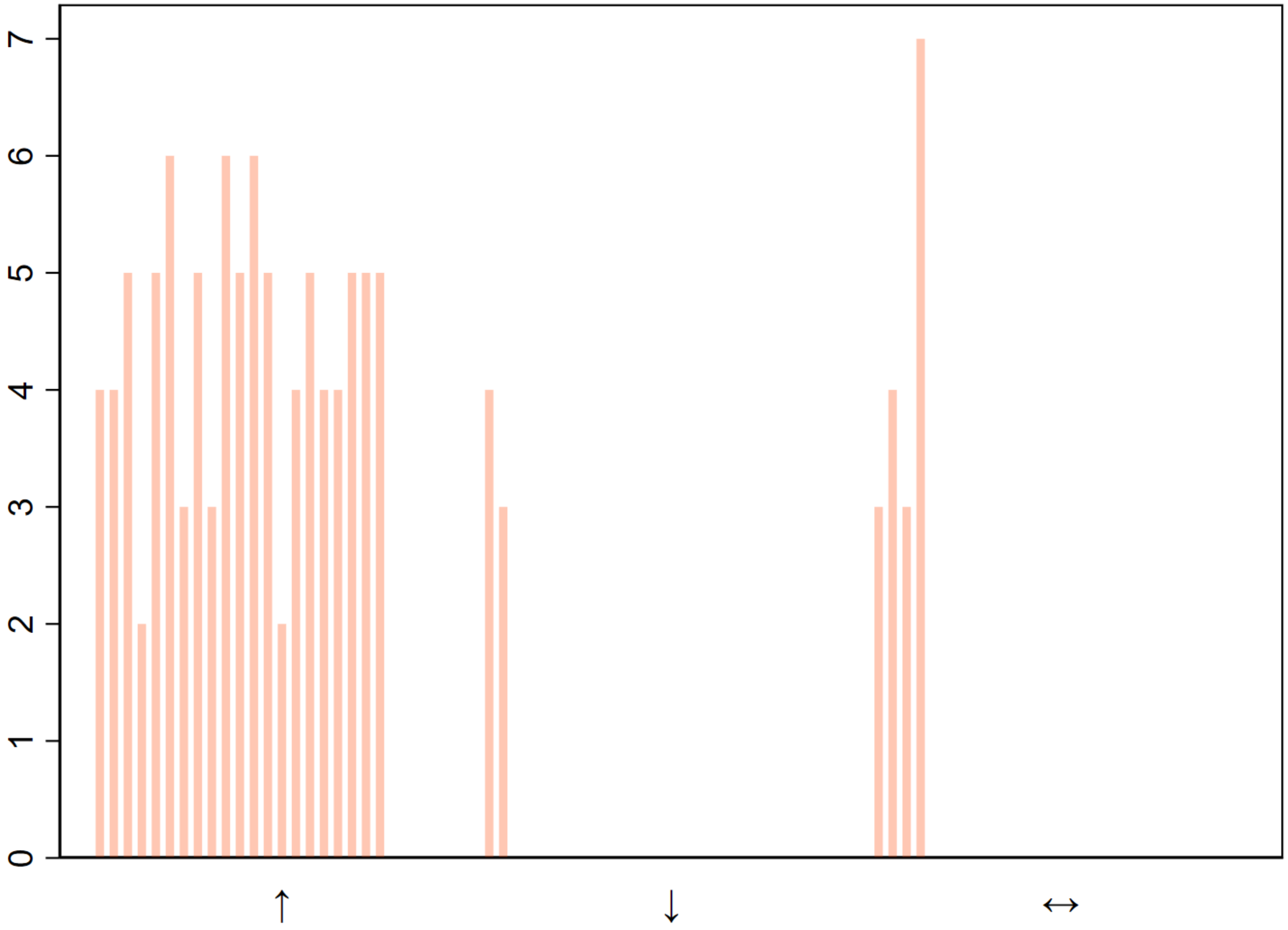

Figure A5.10: overall direction-of-effect scores in core studies, by overall quality score. See Fig. A5.9.

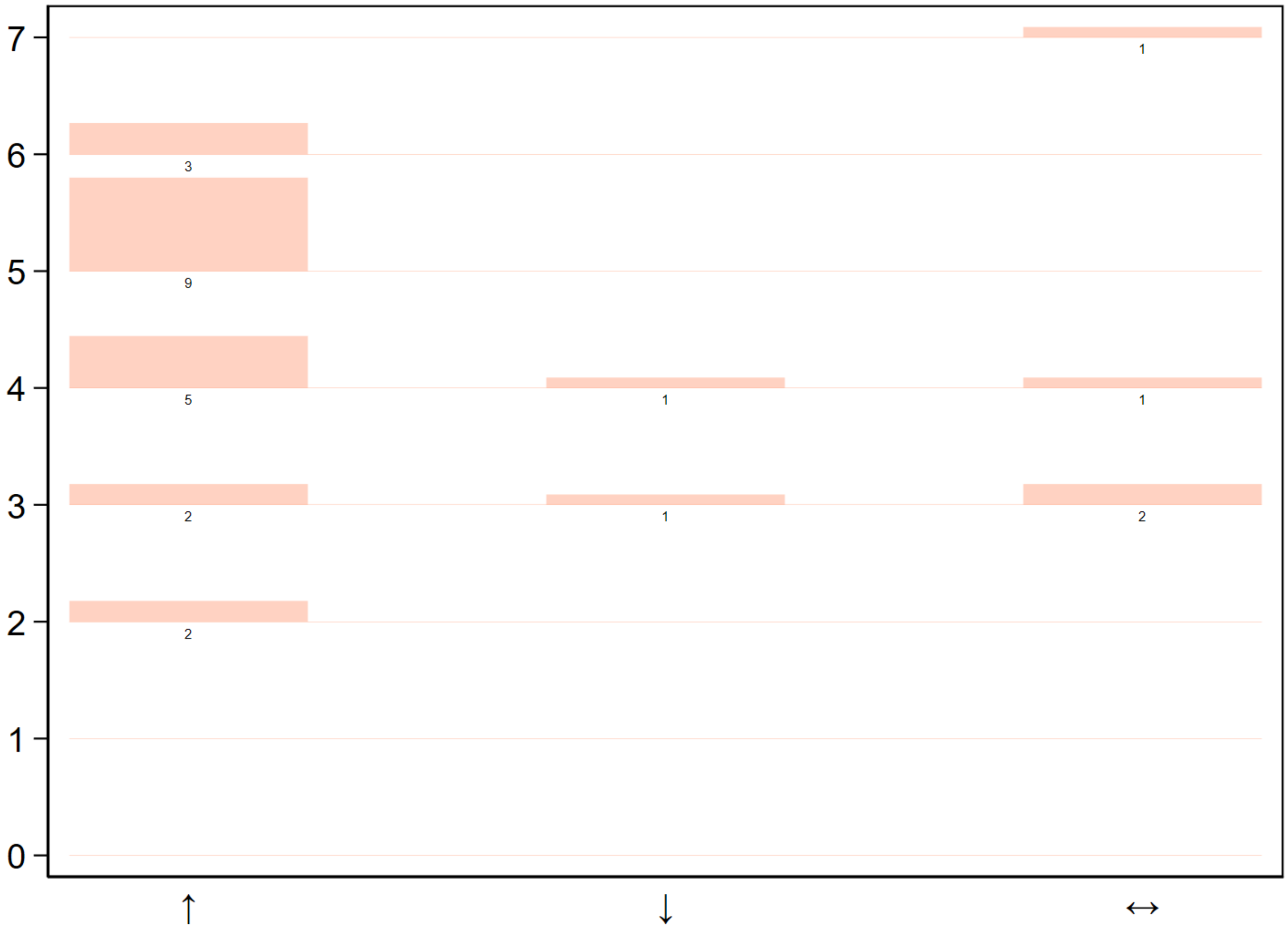

Table A5.1: detailed table of findings, core studies. See Table 1 for legend.

D_i	1st Author / year	Sanction episode	Country	Health-related outcomes	Health system outcomes	Methods	Main findings	Modification/mediation	Limitations / Comments
▲	Al-Ani et al (2011)[35]	UN sanctions (Aug 1990–May 2003).<br><br>Correlated shock: First Gulf war (Jan–Feb 1991).	Iraq (city of Haditha)	IMR		Descriptive statistics from time-series of administrative register (Sample: 59424 live births).	▲ Average annual IMR was 34.7 in 1987–1988, 29.5 in 1989-1990, 38.8 in 1991–2002, 28.6 in 2003–2007 and 27.7 in 2008–2010. The highest and lowest values were 46 in 2002 and 16 in 2006.<br><br>IMR declined from 1987 to 1990, increased from 1991 to 2002, decreased until 2006, fluctuated upward until 2008 and declined afterwards.	Increase during 1991–2002 attributed to sanctions.	Confounding: no adjustment for armed conflict (if lagged effect).<br><br>Selection: no adjustment for changes in reporting of vital events.<br><br>Modification/mediation: no analysis of interaction with armed conflict (if lagged effect).<br><br>Other: some comparisons with national and regional figures rely on findings from UNICEF's ICMMS survey, the reliability of which has been questioned.<br><br>$\mathbf{Q}_i = 4$
▲	Ali (2004)[36]	UN sanctions (Aug 1990–May 2003).<br><br>Correlated shock: First Gulf war (Jan–Feb 1991).	Iraq (city of Mosul)	Cases of viral hepatitis B	Hepatitis B vaccine availability (doses per year)	Descriptive statistics from facility register (Sample: 254 children aged 2–7)	▲ Of 254 children referred to screening for Hepatitis B in June 2000 to June 2001, 74 tested positive, and 62 of them attended an immunization clinic. A majority of those (32/62) were born in the two years registering lower availability of vaccine doses, while the lowest number of births occurred in the year registering the highest availability (8/62).	Variation in vaccine stocks attributed to sanctions and the implementation of the OFFP.	Selection: no control for changes in facility enrolment.<br><br>Measurement error in exposure: unclear definition of exposure groups (OFFP); limited information on exposure (OFFP).<br><br>Modification/mediation: no analysis of interaction with armed conflict (if lagged effect).<br><br>Other: no information on target population.<br><br>$\mathbf{Q}_i = 4$
▲	Asadi-Pooya et al (2019)[37]	US sanctions (Nov 1979–ongoing), esp. measures reintroduced	Iran (cities of Shiraz, Tehran, Ahvaz)	Self-reported changes in seizure control.	Self-reported difficulty in obtaining antiepileptic medication.	Pearson Chi-square test on cross-sectional survey from 3 facilities	▲ Significantly larger share of patients on imported medications reporting 'significant hardship' in obtaining treatment after	Impact attributed to shortage of imported pharmaceutical intermediate and finished products, caused by	Measurement error in exposure: unclear definition of exposure period.

D_i	1st Author / year	Sanction episode	Country	Health-related outcomes	Health system outcomes	Methods	Main findings	Modification/mediation	Limitations / Comments
		after withdrawal from the Joint Comprehensive Plan of Action (Aug 2018–ongoing).				(Sample: 244 patients with more than a year of epilepsy diagnosis).	tightened sanctions than patients on domestic medications (72%, 30%, p<0.0001).<br>▲ Incidence of seizures significantly higher in patients experiencing significant hardship in obtaining treatment than in patients who reported no difficulty (36%, 22%, p<0.05).	restrictions on international transactions due to sanctions	Measurement error in outcome: only self-reported outcomes (no control for recall bias or biased reporting).<br><br>Other: limited information on target population.<br><br>$\mathbf{Q}_i = \mathbf{5}$
▲	Ascherio et al (1992)[14]	UN sanctions (Aug 1990–May 2003).<br><br>Correlated shock: First Gulf war (Jan–Feb 1991).	Iraq	IMR, U5MR, under-1 month, 1–12 months and 12–60 months mortality rates.		Unadjusted and adjusted (Poisson regression) relative mortality rates from cross-sectional survey (Sample: 16172 live births).	▲ Relative to various baseline periods before 1991, in the first 8 months of 1991 under-1 month mortality was 1.8 (95% CI 1.4–2.4) times higher; 1–12 months mortality was 4.1 (95% CI 3.3–5.2) times higher; 12–60 months mortality was 3.8 (95% CI 2.6–5.4) times higher. Controlling for age of child and mother, education of mother and area of residence, estimates are 1.8, 4.3, 5.2 respectively.<br><br>The associated IMR and U5MR changes between the two periods are 32.5 to 92.7 and 43.2 to 128.5, respectively.<br><br>Extrapolating results to a projected national population with stable growth and fertility returns 46900 excess child deaths in the first 8 months of 1991.	Between the two periods, age-adjusted mortality due to diarrhoea rose from 2.1 to 11.9 per 1000 person-years.	Confounding: no control for armed conflict.<br><br>Selection: no adjustment for changes in mortality of eligible respondents.<br><br>Modification/mediation: no analysis of interaction with armed conflict.<br><br>Missing data: incomplete cases excluded.<br><br>Measurement error in outcome: only self-reported outcomes (no control for recall bias in birth histories).<br><br>Others: limited adjustment of sampling frame for population change due to armed conflict and sanctions; no clustering of standard errors at survey cluster level; possible bias in excess deaths due to fertility decline during war and sanctions.<br><br>$\mathbf{Q}_i = 2$
▲	Bundervoet and Verwimp (2005)[38]	Sanctions by the Great Lakes Regional Peace Initiative (Jul 1996–Jan 1999).	Burundi	Height-for-age		OLS and logit regressions on cross-sectional survey (Sample: 2575 children	▲ In rural areas, a percentage point increase in exposure to sanctions during the early growth spurt (6–24 months) reduces height-for-age by 0.005 (p<0.01)	If monthly exposure to sanctions is replaced by a set of relative food prices, the price of beans has a significant and negative effect on height	Selection: no control for selective migration, mortality selection.<br><br>Modification/mediation: no control for exposure-

D_i	1st Author / year	Sanction episode	Country	Health-related outcomes	Health system outcomes	Methods	Main findings	Modification/mediation	Limitations / Comments
		Correlated shock: Burundian civil war (Oct 1993–Aug 2005)				aged 6–59 months).	standard deviations, conditional on any exposure to armed conflict – which has a similar effect size. The effect implies a -0.5 standard-deviation for exposure to sanctions throughout the early growth spurt period. Insignificant positive effect of sanctions in urban areas.	for age. This price is thus suggested as a channel of the impact of sanctions.	mediator interaction (food prices) and armed conflict (if lagged effect).<br><br>$\mathbf{Q}_i = \mathbf{5}$
▲	Daponte and Garfield (2000)[13]	UN sanctions (Aug 1990–May 2003).<br><br>Correlated shock: First Gulf war (Jan–Feb 1991).	Iraq	Under-6 mortality risk		Adjusted relative risks (Cox regression) on cross-sectional survey (Sample: 14118 children under 6).	▲ Controlling for maternal education and age, child sex, parity and urban vs. rural location, the risk of death for the reference group (rural males born from primiparous women aged 25–29 with high-school education) increased by 4.54 (β 1.51, SE 0.09) after the imposition of sanctions (January 1985–August 1990 vs. September–December 1990).	Impact attributed to sanctions, including potential hoarding due to anticipated shortages.	Missing data: incomplete cases excluded.<br><br>*Notes*: Re-analysis of subsample from Ascherio et al (1992).<br><br>$\mathbf{Q}_i = \mathbf{6}$
▲	Garfield (2001)[39]	Sanctions by UN, EU and US (May 1991– Jan 2001, with gaps).<br><br>Correlated shock: Yugoslav wars and breakup (Mar 1991–Nov 2001).	Serbia-Montenegr o	Prevalence of undernutrition and low birthweight (Kg<2.5); IMR, U5MR, maternal mortality rate, deaths from CVDs, 5-year survival after bone cancer treatment.	Health expenditure per capita, water quality.	Descriptive statistics from national datasets, household survey, secondary literature; field observations from focus groups and semi-structured interviews (Sample: not shown).	▲ Between 1991 and 1993 IMR and U5MR increased 15.4 to 17.3 and 17.6 to 19.4 respectively, temporarily reversing previous decline.<br><br>▲ Deaths from cardiovascular diseases increased from about 50000 in 1990 to around 60000 in 1996.<br><br>▲From 1996 to 2000, prevalence of under-5 acute and chronic undernutrition increased from 0.05% to 1.9% and from 2.1% to 5% respectively. Insignificant increase in the prevalence of low birthweight.	▲ Five-year survival rate after bone cancer treatment declined from 50% (late 1980s) to 33% (late 1990s) due to decline in the cure rate from 90% to 10%.<br><br>▲ Health expenditure per capita declined from 200 to 40 USD in 1990–1999.<br><br>▲ Share of contaminated water samples rose from 30% in 1989 to a 30/40% range in 1992–1998.	Confounding: no control for armed conflict and political instability; limited control for pre-sanctions trends.<br><br>Modification/mediation: no analysis of interaction with armed conflict.<br><br>Missing data: sparse data (data gaps within exposure periods).<br><br>Reporting of results: some data in graphical display only.<br><br>$\mathbf{Q}_i = \mathbf{3}$
▲	Garfield and Leu (2000)[19]	UN sanctions (Aug 1990–May 2003).	Iraq	U5MR (predicted)		Prediction from logistic regression on cross-sectional survey and	▲ Predicted mortality increases from 36 (95% CI 33–40) in 1990 to 103 (95% CI 90–119) in 1991 and 87 (95% CI 80–95) in 1996.	Increase in undernutrition, decline in adult literature and access to potable water.	Confounding: limited sensitivity analysis to alternative specifications; no control for armed conflict (if lagged effect).

D_i	1st Author / year	Sanction episode	Country	Health-related outcomes	Health system outcomes	Methods	Main findings	Modification/mediation	Limitations / Comments
		Correlated shock: First Gulf war (Jan–Feb 1991).				national statistics (Sample: 195 countries)	The 1990 prediction is not statistically different from the IST survey estimate (Ascherio et al, 1992). The prediction for 1991 is statistically different and smaller, a fact attributed to war-related factors omitted in the model.		Modification/mediation: no analysis of interaction with armed conflict (if lagged effect).<br><br>$\mathbf{Q}_i = \mathbf{5}$
▲	Garfield and Santana (1997)[40]	US embargo (Jul 1963–ongoing), esp. Cuban Democracy Act (Oct 1992–ongoing).<br><br>Correlated shock: economic crisis of *período especial* (1991–2000)	Cuba	Prevalence of low birth weight (Kg<2.5), TB incidence, IMR, MMR, all-cause, all-age cause-specific mortality rates.	Per capita calorie and protein availability, coverage of chlorinated water supply, value of medical imports, lab exams and x-rays per year, size of national formulary.	Descriptive statistics from national datasets and other secondary sources (Sample: not shown).	▲ Small temporary increase in IMR between 1993 and 1994, MMR increased in some years throughout 1988–1996. All-cause mortality rose from 6.4 per 1000 in 1989 to 7.2 in 1994. From 1989 to 1993, deaths per 100000 rose 8.3 to 13.9 for infectious and parasitic diseases, 23 to 40.7 for influenza and pneumonia.<br><br>▲ Prevalence of low birthweight increased from 7.3% in 1989 to 9% in 1993.<br><br>▲ TB incidence rose 5.5 per 100000 in 1990 to 15.3 in 1994.	▲ Per capita protein and calorie availability declined by 25% and 18% in 1989–1992.<br><br>▲ Share of people covered by chlorinated water declined from 98% to 26% in 1990–1994.<br><br>▲ In 1990–1994 laboratory exams declined 36%, x-rays declined 75%, the national formulary shrunk from 1300 to 889 items.<br><br>▲ The value of medical imports was 227 mln. USD in 1989, 67 in 1993, 104 in 1995.	Confounding: limited control for pre-sanction trends, no control for economic crisis.<br><br>Modification/mediation: no analysis of interaction with economic crisis.<br><br>Missing data: sparse data (data gaps within exposure periods).<br><br>Reporting of results: some data in graphical display only.<br><br>$\mathbf{Q}_i = \mathbf{3}$
▲	Ghiasi et al (2016)[41]	US and EU sanctions against Central Bank of Iran (unspecified).	Iran (city of Tehran, 22 districts)		Availability of asthma medicines.	Student *t* test on panel survey (Sample: 40 community pharmacies).	▲ After tightened sanctions (July 2012–March 2013), availability declined by 15 (p<0.1), 35 (p<0.01), 42.5 (p<0.01) and 17.5 (p<0.05) percentage points for four classes of imported medicines; and by 7.5 (not significant), 25 (p<0.05), 32.5 (p<0.01), 20 (p<0.1), 32.5 (p<0.01) and 20 (p<0.1) percentage points for six classes of domestically produced medicines.	Decline attributed to sanctions on imports of finished products and intermediate inputs.	Measurement error in exposure: limited information on exposure.<br><br>Other: limited information on target population.<br><br>$\mathbf{Q}_i = \mathbf{6}$
▲	Gutmann et al (2021)[42]	32 sanction episodes, 1977–2012.	98 less developed and newly	Life expectancy at birth.		WLS regression after matching (entropy	▲ UN sanctions associated to life expectancy reductions of 1.16 and 1.44 years for	UN sanctions increase U5MR by 4.25% (p<0.05) and cholera	Confounding: no control for pre-sanction outcome trend and natural disasters.

D_i	1st Author / year	Sanction episode	Country	Health-related outcomes	Health system outcomes	Methods	Main findings	Modification/mediation	Limitations / Comments
			industrializ ed countries			balancing) on country panel (Sample: 2483 country-years before matching).	men and women respectively (p<0.01). US sanctions associated to life expectancy reductions of 0.37 (p<0.1) and 0.46 (p<0.05) for men and women respectively. Differences across sex and sender are statistically significant. Annual impacts become significant after 2 years, except that of UN sanctions on women – which is always significant. The impact becomes not significantly different, and significantly larger for men, after 8 and 3 years for UN and UN sanctions respectively.<br><br>The impact of US sanctions decreases with distance to the US. Estimates broadly robust to control for unimplemented sanction threats, excluding episodes longer than 10 years, excluding Cold War period.	deaths by 1.08% (p<0.01) for the first 2 and 3 years respectively; and decrease per capita healthcare spending by 0.3% (p<0.01) every additional year. No significant effect of US sanctions.<br><br>These outcomes are interpreted as possible channels of sanction effects on life expectancy.	Modification/mediation: no control for pre-existing outcome differences by sanctioning party, no control for multiple mediators.<br><br>Other: major episodes excluded (Cuba, Iraq, Iran, Serbia-Montenegro).<br><br>$\mathbf{Q}_i = \mathbf{5}$
▲	Karimi and Haghpanah (2015)[43]	US and EU sanctions in 2012 (unspecified).	Iran (city of Shiraz)	Clinical outcomes for thalassemia (serum ferritin levels, annual transfusions) and hemophilia (arthropathy score, annual bleedings).	Self-reported access to pharmaceutical s	Student-t and Chi-square tests on longitudinal facility register and cross-sectional survey (Sample: 69 thalassemia and 40 hemophilia patients).	▲▲ In hemophilia patients, severity of arthropathy was significantly higher in 2012 (32.5) than in 2006 (23.3, p<0.01) and 2009 (24.5, p<0.01). Average annual bleedings in 2012 (40) were significantly higher than in 2006 (12, p<0.01) and 2009 (13, p<0.01).<br><br>▲▲In thalassemia patients, serum ferritin was higher in 2012 (3201 ng/ml) than in 2006 (2345, p<0.05) and in 2009 (2358, p<0.1). Blood transfusion volume in 2012 (196) was insignificantly higher than in 2006 (195) and 2012 (192).	Self-reported availability of iron chelators and blood clotting factors declined between 2009 and 2012. Domestically produced drugs exhibited smaller reported declines.<br><br>Higher morbidity attributed to drug shortages, and shortages to poor mitigation of sanction impacts.	Measurement error in exposure: unclear definition of exposure groups; limited information on exposure.<br><br>Other: no information on target population.<br><br>$\mathbf{Q}_i = \mathbf{6}$

D_i	1st Author / year	Sanction episode	Country	Health-related outcomes	Health system outcomes	Methods	Main findings	Modification/mediation	Limitations / Comments
							No change across 2006–2009 was significant.		
▲	Kheirandish et al (2018)[44]	US and EU sanctions against Central Bank of Iran in 2011 and 2012 (unspecified).	Iran		Monthly averages of Defined Daily Dose per 1000 population (DID), mg per 1000 population/day, unit dose per 1000 population/day, for diabetes, asthma, cancer, and multiple sclerosis.	Chow test for structural break (OLS and GLS models) on time series (Sample: 26 time series of 68 months).	▲ Statistically significant reductions in 13 of 26 therapeutic groups: 6 included only imported products, 1 included both imported and domestic products, and 6 included both imported and domestic products. Other 10 groups showed statistically insignificant reductions.	Macroeconomic effects (currency devaluation, inflation, income declines), inadequate policies (supply chain mismanagement, regulatory capture).	Confounding: limited discussion of model specification.<br><br>Measurement error in exposure: unclear definition of exposure groups; limited information on exposure; no sensitivity analysis to alternative definition of exposure periods.<br><br>$\mathbf{Q}_i = \mathbf{5}$
▲	Kim (2019a)[45]	51 sanction episodes, 1990–2012	64/84 LMICs	Female proportion of above-15 HIV/AIDS prevalence		Fixed effects regression (country level) on country panel (Sample: 584/600 country-years)	▲ Sanctions increase the female proportion of HIV-AIDS prevalence by 0.66 (p<0.05) percentage points, a 1.5% increase at the sample mean (42.94).	If female labor market participation is included in the model, a negative impact is estimated (0.412, p<0.01) and the coefficient of sanctions is one-fourth smaller (0.50, p<0.05).<br><br>Reduced labor market participation is proposed as a channel to higher female prevalence.	Confounding: no control for armed conflict and natural disasters; limited control for pre-sanction outcome levels; no control for pre-sanction outcome trend; some controls potentially affected by exposure.<br><br>Measurement error in exposure: no sensitivity analysis to alternative exposure dataset.<br><br>Modification/mediation: no control for exposure-mediator interaction (labor market participation).<br><br>Measurement error in outcome: no sensitivity analysis to alternative outcome dataset.<br><br>Reporting of results: various tests not reported.<br><br>Other: major episodes excluded (Cuba, Iraq, Iran, Haiti).<br><br>$\mathbf{Q}_i = \mathbf{2}$

D_i	1st Author / year	Sanction episode	Country	Health-related outcomes	Health system outcomes	Methods	Main findings	Modification/mediation	Limitations / Comments
▲	Kim (2019b)[46]	51 sanction episodes, 1990–2012	59/71 LMICs	Ratio of new HIV/AIDS cases among under-15 children, under-15 crude HIV-related death rate.		Fixed effects regression (country level) on country panel (Sample: 740/878 country-years)	▲▲ Sanctions increase the ratio of new child HIV cases by 11.4% ($p<0.01$) and the crude HIV-related death rate by 4.9% ($p<0.1$).		Confounding: no control for armed conflict and natural disasters; limited control for pre-sanction outcome levels; no control for pre-sanction outcome trend; some controls potentially affected by exposure.<br><br>Measurement error in exposure: no sensitivity analysis to alternative exposure datasets.<br><br>Measurement error in outcome: no sensitivity analysis to alternative outcome datasets.<br><br>Other: major episodes excluded (Cuba, Iraq, Iran, Haiti).<br><br>$\mathbf{Q}_i = \mathbf{4}$
▲	McLean and Whang (2019)[47]	602 sanction episodes, 1945–2005.	201 countries	Individuals in need of disaster relief per event.	Public spending on disaster preparedness and defense as a share of GDP; annual value of disaster-related economic losses per event.	Mixed-effects regression on country panel (Sample: disaster outcomes, 3009 country-years; public expenditure, 239/115 country-years)	▲ Sanctions increase by 88% and 95% disaster-related economic losses and population affected, respectively ($p<0.05$). Impacts are reduced after controlling for disaster frequency (66%, 88%) or pre-sanction outcome level (56%, 79%), but remain statistically significant ($p<0.05$).<br><br>Impacts are smaller and marginally significant (71%, 54%, $p<0.1$) for countries with a standardized log GDP per capita below 1.<br><br>The effect is driven by restrictions to target exports and by cases of flood, landslides and storms, relative to other types of sanctions and disasters.	▲ Under sanctions, the GDP share of government spending on disaster preparedness is 18% ($p<0.05$) lower, while no significant reduction occurs for military spending. Controlling for year fixed effects, the impact is reduced but remains statistically significant (-0.08, $p<0.05$).<br><br>For each additional year under sanctions, disaster preparedness spending is 3% ($p<0.05$) lower, military spending is 1% ($p<0.05$) higher.	Confounding: limited control for pre-sanction outcome level, no control for pre-sanction outcome trend; some controls potentially affected by exposure.<br><br>Measurement error in exposure: no sensitivity analysis to alternative exposure dataset<br><br>Other: major episodes excluded (Iran post-2006)<br><br>$\mathbf{Q}_i = \mathbf{5}$

D_i	1st Author / year	Sanction episode	Country	Health-related outcomes	Health system outcomes	Methods	Main findings	Modification/mediation	Limitations / Comments
▲	Mladenovich and Langeggen (2009)[48]	Sanctions by UN, EU and US (May 1991–Jan 2001, with gaps).<br><br>Correlated shock: Yugoslav wars and breakup (Mar 1991–Nov 2001)	Serbia-Montenegro (city of Belgrade)	Risk of retinopathy		Unadjusted odds ratios between birth cohorts from school-level survey (Sample: 165 visually impaired children).	▲ Students born in 1990 and after were 2.4 (95% CI 1.2–4.6) times more likely to be blind than those born before 1990; if blind, they were 1.63 (95% CI 1.08–2.46) more likely to be blind due to retinopathy of prematurity.	Increase in preterm delivery attributed to maternal stress due to armed conflict and sanctions.	Confounding: no control for armed conflict and political instability.<br><br>Selection: no control for changes in facility enrolment, mortality selection.<br><br>Modification/mediation: no analysis of interaction with armed conflict.<br><br>$\mathbf{Q}_i = \mathbf{4}$
▲	Mulder-Sibanda (1998)[49]	Embargo by OAS (Oct 1991–Aug 1993) and UN (Jun–Aug 1993, Oct 1993–Oct 1994).<br><br>Correlated shock: political instability (1987–1991).	Haiti	Prevalence of undernutrition (stunting, underweight, wasting), 1–4 mortality rate.		Descriptive statistics from household surveys (Sample: nutritional surveys, 5004 (1978), 1718 (1990), 2502 (1994) children aged 6–59 months; 1–4 mortality, not shown).	▲ After declining from 77 to 63 and 56 in 1975–9, 1980–4, and 1985–9 respectively, 1–4 mortality increased to 61 in 1994–5.<br><br>▲ Prevalence of stunting, underweight, and wasting declined 12.1%, 32.6%, and 47.5% respectively in 1978–1990. Between 1990 and 1994–5, stunting declined 4.1%, underweight and wasting increased 6% and 96.5%.		Confounding: no adjustment for political instability (if time-varying) and seasonality.<br><br>Missing data: sparse data (data gaps within exposure periods).<br><br>Measurement error in outcome: limited control for changes in definition of undernutrition across surveys.<br><br>Other: no adjustment of survey weights for population change due to political instability.<br><br>*Notes*: Data before 1994–5 already used in Berggren et al (1993).<br><br>$\mathbf{Q}_i = \mathbf{4}$
▲	Parker et al (2016)[50]	Dodd-Frank Wall Street Reform and Consumer Protection Act, Section 1502 'conflict mineral' clause and associated	Democratic Republic of Congo (5 provinces).	IMR		Linear probability model on cross-sectional survey (Sample: 7697 live births).	▲ Under sanctions, villages in targeted areas and close to at least one mining site experienced an increase in infant mortality between 86 and 182 per 1000, from a pre-sanction baseline of 60 per 1000, according to specification.	Baseline estimates suggest the role of proximity to 3T mining activity. Further evidence is provided that women in treatment villages were less likely to sleep under a bed net and to receive prenatal care, consistent with adverse effects of	Selection: limited control for selective migration.<br><br>Modification/mediation: no control for exposure-mediator interaction (armed conflict).<br><br>$\mathbf{Q}_i = \mathbf{5}$

D_i	1st Author / year	Sanction episode	Country	Health-related outcomes	Health system outcomes	Methods	Main findings	Modification/mediation	Limitations / Comments
		actions (Jul 2010–ongoing)					Results broadly robust to controls for mother fixed effects, spatial and temporal definition of exposure, alternative spatial clustering of standard errors.	legislation playing out through reduction in 3T mining, related labour income, and income-elastic demand for health inputs.	
▲	Reid et al (2007)[51]	Embargo by OAS (Oct 1991–Aug 1993) and UN (June–Aug 1993, Oct 1993–Oct 1994).<br><br>Correlated shock: political instability (1987–1991).	Haiti (Grand Anse department, Jeremie county)	Risk of death 12 months after enrolment (first visit), prevalence of undernutrition (underweight).		Adjusted relative risks (Cox regression) on facility panel data (Sample: 1593 children under 2 at first visit)	▲ Controlling for undernutrition at enrolment, the risk of death is 4 (β 1.39, SE 0.34) times higher under sanctions (1992–1994) and 2 (β 0.74, SE 0.56) times higher before sanctions (1989–1992) than afterwards (1995–1996)	Significantly higher prevalence of undernutrition under sanctions. In regressions, no significant interaction between undernutrition and sanctions and between child sex and sanctions.<br><br>Mortality increase attributed to rising prevalence of undernutrition and non-nutritional factors.	Confounding: some controls potentially affected by exposure.<br><br>Selection: limited control for changes in facility enrolment.<br><br>$\mathbf{Q}_i = \mathbf{5}$
▲	Sharma et al (2017)[52]	India-Nepal border blockade (Sep 2015–Feb 2016).<br><br>Correlated shock: Gorkha earthquake and aftershock (Apr–May 2015).	Nepal		Unit prices of 5 categories of health commodities	Forecast from nonlinear regression (Sample: 4 times series of 67 monthly data)	▲ Under blockade, monthly unit price of 'medicaments, therapeutic, prophylactic use, in dosage form' was significantly higher than the pre-blockade trend, entailing an estimated extra cost of 22.3 mln. USD. No significant deviation from pre-earthquake trend in the period between earthquake (April–May 2015) and blockade.	No significant deviation from trend for selected sub-categories.<br><br>Price increase attributed to supply constraint due to blockade.	Confounding: limited discussion of model specification, no sensitivity analysis to alternative specifications.<br><br>Modification/mediation: no analysis of interaction with natural disaster (if lagged effect).<br><br>$\mathbf{Q}_i = \mathbf{5}$
▼	Asadi-Pooya et al (2016)[53]	US, UN, EU sanctions in 2012 (unspecified)	Iran (city of Shiraz)	Incidence of seizure-free state.	Self-reported missed doses per month and causes of poor drug adherence.	Chi square, Fischer's Exact and Mann-Whitney U tests on facility longitudinal register. (Sample: 199 patients diagnosed with epilepsy)	▼ ▼ Under sanctions (March 2010–11 vs. September 2012–13) there were statistically insignificant increases in satisfactory adherence to treatment (139, 146, p>0.1), and seizure-free patients (106, 111, p>0.1).	Statistically significant decrease in poor adherence due to 'other reasons' (14, 4, p<0.05), marginally significant increase in poor adherence due to 'price and availability issues' (3, 8, p<0.1).<br><br>Increase of poor adherence due to 'price and availability issues' attributed to intensified	Measurement error in exposure: limited information on exposure.<br><br>Missing data: cases with insufficient follow-up visits excluded.<br><br>Measurement error in outcome: only self-reported outcomes.<br><br>$\mathbf{Q}_i = \mathbf{4}$

D_i	1st Author / year	Sanction episode	Country	Health-related outcomes	Health system outcomes	Methods	Main findings	Modification/mediation	Limitations / Comments
								sanctions, attenuated by government subsidies.	
▼	Joury et al (2016)[54]	UN sanctions (Aug 1990–May 2003).<br><br>Correlated shock: First Gulf war (Jan–Feb 1991).	Iraq	Prevalence of type-2 diabetes, overweight/obesity, dental caries.	Free-sugars consumption.	Descriptive statistics from published literature (Samples: not shown).	▼ Prevalence of type-2 diabetes among adults increased from 4.1% under sanctions (2000) to 6.5% afterwards (2006).<br><br>▼ In 2000 and 2006 respectively, overweight prevalence increased from 5.5% to 34.1% in adults aged 25 to 65, and from 6% to 13.6% in urban children aged 6 to 13. For this latter group, obesity increased from 1.3% to 10.5%.<br><br>▼ Dental caries prevalence in children aged 5–6 fell from 80.5% in 1985 to 61.2% in 1995 in urban areas, and from 36.5% to 7.1% and 18.8% in 2004–5 in rural areas; in children aged 11–12, prevalence went from 82.6% in 1985 to 66.1% in 1995 in urban areas and from 39.1% to 12.3% in rural areas; among children aged 14–15, prevalence went from 87.7% in 1985 to 69.9% in 1995 in urban areas, and from 41.2% to 15.4% in rural areas.	▼ Average annual sugar consumption per person went from 50 to 16.3 to 24.1 Kg before (1984–90), during (1991), and after sanctions (2005) respectively.	<u>Confounding:</u> no adjustment for armed conflict (if lagged effect).<br><br><u>Modification/mediation</u>: no analysis of interaction with armed conflict (if lagged effect).<br><br><u>Missing data</u>: sparse data (data gaps within exposure periods and no data for some exposure periods).<br><br><u>Measurement error in outcome</u>: no comparability of sources for some exposure periods.<br><br>$\mathbf{Q}_i = \mathbf{3}$
◀▶	Berggren et al (1993)[55]	Embargo by OAS (Oct 1991–Aug 1993) and UN (Jun–Aug 1993, Oct 1993–Oct 1994).<br><br>Correlated shock: political instability (1987–1991).	Haiti	IMR, U5MR, 1–4 mortality rate, deaths due to measles, prevalence of undernutrition.		Descriptive statistics from national datasets, household surveys, facility registers; field observations and interviews (Sample: facility register, 3168 live births).	▲▲ Under sanctions (1992), relative to the pre-sanction period (1991), a facility register shows infant mortality declining 48 to 39; 1–4 and under-5 mortality increasing 10 to 18 and 87 to 115 respectively; measles mortality increasing 1% to 14%.<br><br>Under alternative scenarios, extrapolating the absolute or percentage increase in	Blackouts and fuel shortages leading to disrupted cold chain, and the interruption of relations between the government and international organization involved in vaccination, are proposed as mediators of the contribution of sanctions to declines in measles vaccine coverage and rising measles mortality.	<u>Confounding</u>: no adjustment for political instability (if time-varying).<br><br><u>Selection</u>: no adjustment for changes in facility enrolment.<br><br><u>Measurement error in exposure</u>: unclear definition of exposure period.

D_i	1st Author / year	Sanction episode	Country	Health-related outcomes	Health system outcomes	Methods	Main findings	Modification/mediation	Limitations / Comments
							U5MR to the national level returns excess deaths estimates ranging between 1200 and 24000 for the 1991–1992 period.<br><br>◄► Annual data from 38 fixed surveillance stations from 1991 to 1993 fails to show consistent trends in prevalence of under-5 undernutrition across the country and in 4 large regions.		Missing data: sparse data (data gaps within exposure periods).<br><br>$\mathbf{Q}_i = \mathbf{3}$
◄►	Peksen (2011)[56]	96 sanction episodes, 1970–2000.	154 countries	U5MR		OLS regression on country panel (Sample: 4055/4209 country-years).	◄► Insignificant effect of any sanctions, positive or negative depending on specifications.<br><br>▼ Insignificant negative effect of multilateral sanctions.<br><br>▲ US sanctions are associated to a 22.7% to 28.7% increase in U5MR (p<0.01), according to specifications.<br><br>Results broadly robust to an alternative U5MR dataset.	A 1 percentage-point increase in sanction severity score (losses as a share of GPD) increases U5MR by 0.7 to 1% (p<0.05), implying a circa 4% increase for a one-standard-deviation change at the sample mean.<br><br>No significant effect of any sanctions across GDP levels.	Confounding: limited control for baseline outcome level, no control for pre-sanction outcome trend and natural disasters; some controls potentially affected by exposure.<br><br>Measurement error in exposure: no sensitivity analysis to alternative exposure dataset.<br><br>Missing data: limited control for missing outcome data (no reporting of imputation statistics, no sensitivity analysis to excluding imputed data).<br><br>$\mathbf{Q}_i = \mathbf{4}$
◄►	Petrescu (2016)[57]	45 sanction episodes, 1914–2006.	69 countries	Infant weight, under-3 height, under-3 mortality risk.		OLS regression on pooled cross-sections (Sample: 70114 infants, 187099 or 22827 under-3 children).	▲ One-month *in utero* exposure to sanctions associated to a 0.008 (p<0.05) standard deviation decrease in infant weight.<br><br>▲ One-month *in utero* exposure to sanctions associated to a statistically insignificant increase in mortality risk.<br><br>▼ One-month *in utero* exposure to sanctions	One-month *in utero* exposure to sanctions associated to a 0.001 (p<0.05) increase in mortality risk in the first year of sanctions; a 0.002 (p<0.05) increase in mortality risk and -0.01 (p<0.1) standard deviation in infant weight in the second year of sanctions. No significant effects in the third year and for height.	Confounding: no control for political instability, pre-sanction outcome level and trend; some controls potentially affected by exposure.<br><br>Selection: no control for changes in mortality in surveyed children.<br><br>Measurement error in exposure: no sensitivity

D_i	1st Author / year	Sanction episode	Country	Health-related outcomes	Health system outcomes	Methods	Main findings	Modification/mediation	Limitations / Comments
							associated to a statistically insignificant 0.007 increase in height.	Interaction between *in utero* exposure to sanctions and average mortality associated to a decrease in height $(-.17, p < .05)$, conditional on a positive effect of *in utero* exposure $(02, p < .01)$.	analysis to alternative exposure datasets.<br><br>Measurement error in outcome: no sensitivity analysis to alternative outcome datasets.<br><br>Other: major episodes excluded (Cuba, Iraq, Iran, Serbia-Montenegro)<br><br>$\mathbf{Q}_i = \mathbf{3}$
◄►	Zaidi (1997)[18]	UN sanctions (Aug 1990–May 2003).<br><br>Correlated shock: First Gulf war (Jan–Feb 1991).	Iraq (city of Baghdad)	IMR, U5MR		Unadjusted relative mortality rates from panel survey (Sample: not shown).	◄► In 1996, IMR and U5MR are estimated at 33 and 38 per 1000 respectively, much lower than levels for 1995 reported by Zaidi and Fawzi (1995).<br><br>In households followed up from Zaidi and Fawzi (1995), only 9 out of 83 deaths matched, while 65 and 9 were only recorded in the first and second survey respectively. In later interviews with some respondents who reported a death in the first survey only, 9 deaths were confirmed, 4 were found to be miscarriages or stillbirths, and 13 could not be confirmed.	Errors in the reporting or coding of deaths created a large difference in mortality rates with those reported in Zaidi and Fawzi (1995). The author conjectures that an accurate estimate lies between the two estimates.	*Notes*: Follow-up on a subset of 64 clusters sampled by Ascherio et al (1992), 20 of which were sampled also in Zaidi and Fawzi (1995).<br><br>$\mathbf{Q}_i = \mathbf{7}$
**N/A**	Ali and Shah (2000)[21]	UN sanctions (Aug 1990–May 2003).<br><br>Correlated shock: First Gulf war (Jan–Feb 1991).	Iraq	IMR, U5MR		Descriptive statistics and adjusted relative risks (logistic regression) from cross-sectional survey (Sample: 95028 and 60614 live births in South/Centre and North Iraq).	After the imposition of sanctions (1984–89 vs. 1994–19), IMR and U5MR increased from 47 to 108 and from 56 to 131 respectively in the South/Centre, and declined from 64 to 59 and from 80 to 72 respectively in the North.<br><br>Findings are robust to controls for place of residence, sub-region, maternal age and education,		Confounding: no control for armed conflict (if lagged effect).<br><br>Modification/mediation: no analysis of interaction with armed conflict (if lagged effect).<br><br>Reporting of results: findings based on UNICEF's ICMMS survey, the reliability of which has been questioned.

D_i	1st Author / year	Sanction episode	Country	Health-related outcomes	Health system outcomes	Methods	Main findings	Modification/mediation	Limitations / Comments
							child sex and parental blood affiliation.		*Notes*: annualized estimates presented in Ali et al (2003)<br><br>$\mathbf{Q}_i = \mathbf{N/A}$
**N/A**	Ali et al (2003)[58]	UN sanctions (Aug 1990–May 2003).<br><br>Correlated shock: First Gulf war (Jan–Feb 1991).	Iraq	IMR, U5MR		Descriptive statistics from cross-sectional survey (Sample: not shown).	In the South/Centre region, there was a statistically significant increase in IMR and U5MR from 46.8 and 59.4 in 1990 to 98.7 and 116 in 1991, respectively. Such levels were sustained until 1998, when they were 113 and 142.5. For the Northern region, there was a statistically significant increase from 72.4 and 91.9 in 1990 to 103.1 and 128.2 in 1991, reversed in 1992 (68 and 87.7) and 1993 (52.5 and 67.8), with no statistically significant change afterwards.<br><br>Excess deaths attributable to armed conflict and sanctions are computed based on two counterfactuals: constant mortality level at the 1986–1990 average (381947) and constant mortality trend extrapolated from available survey data for the pre-1990 period (481893).		Confounding: no control for armed conflict.<br><br>Modification/mediation: no analysis of interaction with armed conflict.<br><br>Reporting of findings: findings based on UNICEF's ICMMS survey, the reliability of which was later questioned.<br><br>*Notes*: five-year estimates presented in Ali and Shah (2000).<br><br>$\mathbf{Q}_i = \mathbf{N/A}$
**N/A**	Dyson (2006)[24]	UN sanctions (Aug 1990–May 2003).<br><br>Correlated shock: First Gulf war (Jan–Feb 1991).	Iraq	IMR, U5MR		Descriptive statistics from household surveys and population census (Sample: not shown).	Combining survey U5MR and UN population estimates, and assuming a counterfactual of constant U5MR from 1986–1990 to 2003, an estimate of 668000 excess deaths under-5 attributable to armed conflict and sanctions is computed.<br><br>Under a counterfactual of constant mortality decline, extending a linear extrapolation from data over 1960–1990 by Ali et al		Confounding: no control for armed conflict.<br><br>Reporting of findings: findings based on UNICEF's ICMMS survey, the reliability of which was later questioned.<br><br>$\mathbf{Q}_i = \mathbf{N/A}$

D_i	1st Author / year	Sanction episode	Country	Health-related outcomes	Health system outcomes	Methods	Main findings	Modification/mediation	Limitations / Comments
							(2003), the estimate is 878888.		
**N/A**	Zaidi and Fawzi (1995)[17]	UN sanctions (Aug 1990–May 2003).<br><br>Correlated shock: First Gulf war (Jan–Feb 1991).	Iraq (city of Baghdad)	IMR, U5MR, prevalence of under-5 undernutrition (underweight, stunting, wasting).		Unadjusted relative mortality rates and descriptive statistics from panel survey (Sample: 2120 live births, 594 children examined for undernutrition).	Compared to last 1 and 5 years before sanctions, IMR in August 1994 to August 1995 and U5MR in 5 years of sanctions were, respectively, 2 (95% CI 1.15–3.49) and 4.88 (95% CI 3.43–6.94) times higher.<br><br>The prevalence of undernutrition (Z≤-2) increased 12% to 28% (stunting), 7% to 29% (underweight), and 3% to 12% (wasting) from 1991 to 1995.	Effect attributed to sanctions.	Confounding: no control for armed conflict (if lagged effect)<br><br>Selection: no adjustment for changes in mortality of eligible respondents.<br><br>Modification/mediation: no analysis of interaction with armed conflict (if lagged effect).<br><br>Measurement error in outcome: only self-reported outcomes (no control for recall bias in birth histories).<br><br>Reporting of findings: this study has been partially retracted (Zaidi, 1997).<br><br>Others: limited adjustment of sampling frame for population change due to armed conflict and sanctions; no clustering of standard errors at survey cluster level.<br><br>*Notes*: Follow-up on a subset of 25 clusters sampled by Ascherio et al (1992).<br><br>$\mathbf{Q}_i = \mathrm{N/A}$

Pinna Pintor M, *et al. BMJ Global Health* 2023; 8:e010968. doi: 10.1136/bmjgh-2022-010968

Table A5.2: detailed table of findings, non-core studies.

	1st Author / year / Journal	Type of econ sanctions	Country focus	Health-related outcomes	Health system outcomes	Methods	Main Findings	Evidence (or suggestion) of modification/mediation	Comments
1.	Abbara et al (2018)[59]	(unspecified)	Syria		Availability of medical equipment	Commentary based on published sources, secondary survey data, official statistics and media reports	**No original research findings**.<br><br>Together with weak pre-war policies, armed conflict and counterproductive government policies, sanctions are mentioned as a factor restricting the availability of medical supplies necessary to monitor and mitigate opportunities for antimicrobial resistance to evolve. Mentioned items are: spare parts for autoclaves, reagents, agar plates, various disinfection and diagnostic equipment. Energy shortages related to sanctions are also deemed to have made it more difficult to use existing equipment.		
2.	Abbas et al (2008)[60]	UN sanctions (Aug 1990–May 2003).	Iraq	BMI, hemoglobin count, self-reports (age of menarche, average duration of menstrual flow, consanguinity with future husband, family history of inherited conditions)		Descriptive statistics from facility-based cross-sectional survey in Baghdad, 2001.	Prevalence of anemia (<12 g/dl) at 63.2%. Prevalence of overweight ($BMI \geq 25$) and underweight ($BMI \leq 20$) at 24.8% and 6.4%. Mean age of menarche at 13.9. Significant positive age trend for weight and BMI, but not height. Significant positive educational trend for weight, BMI, and hemoglobin level.	Findings interpreted as evidence of maternal malnutrition and ascribed to sanctions.	The absence of baseline prevents obtaining an impact estimate.<br><br>The BMI and anemia severity thresholds employed in presenting defining conditions are not in line with established WHO definitions.
3.	Abdoli (2020)[61]	US sanctions (Nov 1979–ongoing), esp. measures reintroduced after withdrawal from the Joint Comprehensive Plan of Action				Short communication	**No original research findings.**<br><br>Sanctions are regarded as exacerbating the extraordinary pressure put on the healthcare system by the ongoing COVID-19 pandemic, by limiting the		Discussion partially based on the ICMMS survey, the reliability of which has been questioned.

	1st Author / year / Journal	Type of econ sanctions	Country focus	Health-related outcomes	Health system outcomes	Methods	Main Findings	Evidence (or suggestion) of modification/mediation	Comments
		(Aug 2018–ongoing).					import of drugs, PCR and X-ray machines, and equipment for CCU/ICU units; by shrinking fiscal space to finance healthcare workforce expansion and medical research; by hampering international research collaborations with Iranian scholars.		
4.	Afshari and Bhopal (2016)[62]	(unspecified)	Iran			Short communication	**No original research findings**.<br><br>Starting from negligible levels, the number of PubMed-indexed articles with at least one author affiliated to an Iranian institution began to grow in the mid-1980s. The number of articles published in PubMed-indexed Iranian journals follows a similar trend, with a delay of 15 years.		The authors argue that adverse effects of sanctions on international collaborations might have been avoided by an international professional support and the founding of Iranian research journals.<br><br>Given that neither the presence of an Iran-affiliated author, nor the nationality of a research journal are sufficient to distinguish between international collaborations and domestically produced research, it is unclear how informative the figure given is on the issue.
5.	Ahmad (2001)[63]	UN sanctions (Oct 1999–Jan 2002).	Afghanistan (Taliban-occupied territories)	U5MR	Food availability.	Editorial quoting published sources and expert opinions.	**No original research findings.**<br><br>The article is an editorial reporting on the publication of a study by the NGO Médecins Sans Frontières (MSF). Its main finding, a mortality rate of 5.2 per 10000 per day among under-5 Afghani children in refugee camps in North-west		No proposed association with sanctions.

	1st Author / year / Journal	Type of econ sanctions	Country focus	Health-related outcomes	Health system outcomes	Methods	Main Findings	Evidence (or suggestion) of modification/mediation	Comments
							Pakistan, is mentioned. Facts such as distress sales of ploughing animals by households, unavailability of seeds and general inability to farm are also mentioned. The MSF spokesperson is referred to as indicating that food scarcity in Afghanistan forces many people to resort to plant roots. Other sources from UN agencies are quoted or referred expressing concern about deteriorating health conditions in the Afghani population.		
6.	Ahmadi and Meskarpour-Amiri (2015)[64]					Short communication	**No original research findings**.<br><br>Quoting secondary sources, the article expresses the opinion that sanctions, irrespective of exemption policy, can deteriorate population health.		
7.	Ahmed et al (2007)[65]	UN sanctions (Aug 1990–May 2003).	Baghdad, Iraq	Prevalence of dental caries (DMFT index), sugar consumption		Bivariate and multivariate ANOVA on cross-sectional survey of 392 12-years-old secondary school children.	Prevalence of dental caries of 62%.<br><br>In multivariate ANOVA, relative to 'low' maternal education, children of mothers with 'high' education had on average 0.7 more filled teeth ($p < .05$) and a lower score of sugar consumption ($8.5\ vs. 9.6, p < .05$). Sugar consumption score was also higher among boys ($9.5\ vs. 8.7, p < .001$) and among children coming from a low vis-à vis high-SES area ($10.5\ vs. 8.3, p < .001$).<br><br>The opposite sign of the maternal education gradient	Increase in availability of imported sugary products due to lifting of sanctions, leading to higher consumption and prevalence of dental caries.	Impact of sanctions conjectured as a contextual factor, outside the empirical analysis.

	1st Author / year / Journal	Type of econ sanctions	Country focus	Health-related outcomes	Health system outcomes	Methods	Main Findings	Evidence (or suggestion) of modification/mediation	Comments
							on dental caries (positive) and sugar consumption (negative) is interpreted as evidence of an inverse U-shaped income elasticity of demand for imported sugary foods consumption, with lagged health effects and learned moderation.		
8.	Akbarialiabad et al (2021)[66]	US sanctions (Nov 1979–ongoing)	Iran			Literature review	**No original research findings.**		
9.	Akbarpour Roshan and Abbasi (2014)[67]	US embargo (Jul 1963–ongoing), esp. Cuban Democracy Act (Oct 1992–ongoing).	Cuba	Infant, child, maternal, and general mortality; life expectancy	Aggregate food supply, domestic food production, food imports	Commentary based on published sources and statistics from the World Bank, UN agencies and the US Census Bureau	**No original research findings**.<br><br>After the Soviet trade slump and the Cuban Democracy Act, aggregate food supply fluctuated downwards for a few years. The apparent lack of impact on infant and child mortality is attributed to effective food rationing.<br><br>General mortality increased moderately in the two subsequent decades, and is attributed to shortages of specialized treatment for chronic illness.		Narrative discussion of pre-existing findings. The association with sanctions is often assumed uncritically.
10.	Akunjee and Ali (2002)[68]	UN sanctions (Aug 1990–May 2003).	Iraq			Commentary based on published sources and field visit	**No original research findings**.<br><br>Salient topics in the narrative include: deteriorating medical education due to shortage of foreign materials and limited scientific exchange; outmigration of trained specialists and nurses during the Gulf crisis; the role of family relatives as substitutes of nurses in hospitals; shortages of pharmaceuticals, laboratory and X-ray equipment; declining drug quality		No description of conditions under which field observations were made.<br><br>Discussion partly based on FAO/NRI survey findings (Zaidi and Fawzi, 1995), later partly retracted (Zaidi, 1997), and ICMMS survey, the reliability of which has been questioned.

	1st Author / year / Journal	Type of econ sanctions	Country focus	Health-related outcomes	Health system outcomes	Methods	Main Findings	Evidence (or suggestion) of modification/mediation	Comments
							standards due to lower regulation in non-sanctioning exporter countries.<br><br>It is reported that "over 66%" of chemotherapy medication required by the UKALL97 protocol are found to be unavailable in the country.		
11.	Al Faisal et al (2012a)[69]	(unspecified)	Syria			Descriptive statistics based on government sources, UN agencies, and online sources.	In one year after sanctions, the local currency devalued from 45 to 70 USD and a two-fold to four-fold increase occurred in the price of essential goods such as gas, heating oil, vegetable ghee, cheese, yogurt, sugar, milk, vegetable oil, rice, eggs, tea and tomatoes.<br><br>Other adverse consequences attributed to sanctions include: job losses, especially in tourism; energy shortages threatening vaccine refrigeration; heating oil shortages increasing the risk of respiratory tract infections in winter; blocked import of diabetes medication and treatment for cancer and heart disease; disruption to clean water supply		Before-and-after table of prices lacks precise time references, and no indication is given about how prices are derived or computed. Upon inspection, the quoted source of price data could not be retrieved.
12.	Al Faisal et al (2012b)[70]	(unspecified)	Syria			Opinion	**No original research findings.**<br><br>The authors express concerns over the health of Syrians trapped in the current civil war, underlining the role of the local healthcare system and its pre-war achievements. Sanctions are claimed to be a cause of currency		

	1st Author / year / Journal	Type of econ sanctions	Country focus	Health-related outcomes	Health system outcomes	Methods	Main Findings	Evidence (or suggestion) of modification/mediation	Comments
							devaluation, inflation eroding the purchasing power for food and drugs, and decreased capacity to maintain a supply of clean water.		
13.	Al Faisal, Sen (2013)[71]	(unspecified)	Syria			Correspondence	**No original research findings.**<br><br>Correspondence on Sen et al (2013). The authors reply to Maziak et al (2013a) maintaining that the article's focus on sanctions does not entail a partisan position in the conflict, and that the role of sanctions amplifies existing disruptions.		
14.	Al Samaraie (2007)[72]	UN sanctions (Aug 1990–May 2003).	Iraq			Commentary based on published sources.	**No original findings**.<br><br>Sanctions are mentioned as contributing to declines in living standards, health and education.		
15.	Albright (2000)[73]	Iraq: UN sanctions (Aug 1990–May 2003). Cuba: US embargo (Jul 1963–ongoing), esp. Cuban Democracy Act (Oct 1992–ongoing) and Helms-Burton Act (Mar 1996–ongoing).	Iraq, Cuba			Opinion	**No original research findings**.<br><br>Sanctions are an effective instrument for international enforcement of human rights, but measures such as exemptions of food and medicines must be taken to minimize civilian harm. The US government does comply with this qualification, and has allowed trade in such items in the case of Cuba and Iraq. Existing health problems are due to the planned economic model of Cuba, and to the Iraqi's regime boycott of international aid such as the Oil-for-food Programme.		The article has no list of references, and no source is quoted for various individual numbers reported.
16.	Al-Nouri and Al-Raqim (2003)[74]	UN sanctions (Aug 1990–May 2003).	Iraq			Correspondence	**No original research findings**.		Some findings quoted from Ascherio et al (1992), Garfield et al

	1st Author / year / Journal	Type of econ sanctions	Country focus	Health-related outcomes	Health system outcomes	Methods	Main Findings	Evidence (or suggestion) of modification/mediation	Comments
							Estimates of rising infant and child mortality, malnutrition and observations on morbidity and hospital conditions are quoted from academic literature and UN reports, and attributed to sanctions.		(1997), but also from other published sources which fail to refer to original findings (Dobson, 2000) or which refer to the partially retracted findings of Zaidi and Fawzi (1995).
17.	Aloosh and Aloosh (2015)[75]	(unspecified)	Iran			Short communication.	**No original research findings**.<br><br>The article reports an episode of unexpected permanent vision loss in at least 15 patients after eye surgery in Tehran, attributed to sub-standard equipment.		The authors conjecture that the reported case exemplifies a way in which sanctions can affect population health.
18.	Aloosh et al (2019)[76]	(unspecified)	Iran			Commentary based on published sources and official statistics.	**No original research findings.**<br><br>Official GPD figures are quoted showing a recession after the strengthening of sanctions in 2011 and 2012, with recovery after their relaxation due to the Joint Cooperative Plan of Action. Increases in death rates due to self-harm and interpersonal violence are mentioned. Published findings are quoted supporting the occurrence of shortages of essential pharmaceuticals		
19.	Ameri et al (2018)[77]	(unspecified)	Iran		Number, operational status, spatial distribution of facilities; number of staff; available equipment.	Descriptive statistics from cross-sectional survey of 94 radiotherapy facilities in 2015.	The number of operational cobalt-60 machines in the country declined from 25 in 2010 to 8 in 2015.		Impact of sanctions conjectured as a contextual factor, outside the empirical analysis.
20.	Andrews et al (1997)[78]	US embargo (Jul 1963–ongoing), esp. Cuban Democracy Act	Cuba	Self-inflicted injuries		Cases series report from one health facility	Between summer 1994 and late 1995, among detained asylum-seekers in Guantanamo Bay US	Motivation to attain refugee status in the US on medical grounds, given the existing restrictive US refugee policy	The authors mention US sanctions among the factors contributing to the

	1st Author / year / Journal	Type of econ sanctions	Country focus	Health-related outcomes	Health system outcomes	Methods	Main Findings	Evidence (or suggestion) of modification/mediation	Comments
		(Oct 1992–ongoing) and Helms-Burton Act (Mar 1996–ongoing).					military base (30.000 in September 1994), the following cases of self-injury were reported: injection of fuel diesel in the leg and scrotum; burns due to molten plastic; hematuria due to urethral trauma; hemorrhoids due to rectal trauma; laceration of Achille's tendon; asthma due to ingestion of irritants; intestinal obstruction due to ingestion of rocks, metal rings and other objects; uncontrolled hypertension and diabetes mellitus due to noncompliance of treatment abuse. Cases of malingering were also reported, including alleged cases of angina, brain tumor, chronic pain, and other conditions, which later investigation by competent personnel could not verify.<br><br>Self-mutilations ceased after US immigration policy changed, granting refugee status to all detainees. Average outpatient visits fell from 53 per 1000 at the peak of the epidemic (January 1995) to 25 from mid-May to September 1995.	(later reversed) and deteriorating economic conditions in Cuba.	wave of asylum-seekers constituting the population in which the epidemic of self-injuries occurred.
21.	Appleyard (1998)[79]					Correspondence	**No original research findings**.<br><br>The letter reports a resolution by the World Medical Association, urging national professional bodies in sanctioning countries to lobby for effective implementation of exemption systems.		
22.									

	1st Author / year / Journal	Type of econ sanctions	Country focus	Health-related outcomes	Health system outcomes	Methods	Main Findings	Evidence (or suggestion) of modification/mediation	Comments
23.	Arab-Zozani and Ghoddoosi-Nejad (2021)[80]	US sanctions (Nov 1979–ongoing), esp. measures reintroduced after withdrawal from the Joint Comprehensive Plan of Action (Aug 2018–ongoing).	Iran			Short communication	**No original research findings**.<br><br>Sanctions are mentioned as a factor contributing to shortages of personal protective equipment, ventilators, and computed tomography scanners in Intensive Care Units (ICU).		
24.	Aziz (2003)[81]	UN sanctions (Aug 1990–May 2003).	Iraq			Correspondence	**No original research findings**.<br><br>Interviewed Iraqi health professionals are quoted emphasizing the unprecedented severity of injuries presented by patients hit by cluster bombs, the need to ration medications, delays in the treatment of congenital malformations in children, the reliance on superseded pharmaceuticals, and the need to repair basic infrastructure and equipment. Reported shortages of health system inputs and financial resources are attributed to sanctions and government policy before the US invasion,		
25.	Baradaran-Seyed and Majdzadeh (2013)[82]	(unspecified)	Iran			Correspondence	**No original research findings**.<br><br>Comment on Mohammadi (2013), arguing that sanction-induced shortages affect not only pharmaceuticals but also "materials for health services, even sutures and vaccines"	The authors discount currency devaluation and overcompliance by foreign exporters, arguing that shortages are due to financial sanctions, preventing Iranian companies to perform international payments.	
26.	Baram (2000)[83]	UN sanctions (Aug 1990–May 2003).	Iraq			Commentary based on official statistics, survey	**No original research findings**.		Discussion partially based on the ICMMS survey, the reliability

	1st Author / year / Journal	Type of econ sanctions	Country focus	Health-related outcomes	Health system outcomes	Methods	Main Findings	Evidence (or suggestion) of modification/mediation	Comments
						data, UN documents and published literature.	Iraq census data and UN fertility estimates are show to be inconsistent with claims by Iraqi authorities of 1.5 mln. deaths attributable to sanctions, but consistent with a population loss due to war and sanctions comparable with the Iran-Iraq war (250 000 to 500 000 deaths). Claims by the Iraqi government and various international organizations on child mortality attributable to sanctions are contrasted with substantially lower available estimates.<br><br>Data on malnutrition suggests improvements under the Oil-for -food Programme, but frequent absence of nationally representative sampling or disaggregation by governorate level prevents systematic assessment of regional inequalities, which might in part be due to intentional neglect by the government. Available evidence suggests higher prevalence in Southern, mainly Shia districts. The selective focus of some field visits is noted.<br><br>Opportunities to increase imports of food and medicine with the Oil-for-food Programme were not fully exploited by the government until UN pressure ensued. Bias against primary care and breastfeeding in rationing policies and import orders are mentioned.		of which has been questioned.

	1st Author / year / Journal	Type of econ sanctions	Country focus	Health-related outcomes	Health system outcomes	Methods	Main Findings	Evidence (or suggestion) of modification/mediation	Comments
27.	Barnouti (1996)[84]	UN sanctions (Aug 1990–May 2003).	Iraq		Supply of surgical equipment	Correspondence	**No original research findings**.<br><br>The letter characterize surgical practice in Iraq as “far below minimally acceptable standards”, and mentions shortages of antimicrobials, anesthetics, antiseptics, protective equipment, spare parts for cauterizing devices, and suture material, with associated increased risks of mortality and postoperative morbidity.		
28.	Barry (2000a)[85]	US embargo (Jul 1963–ongoing), esp. Cuban Democracy Act (Oct 1992–ongoing) and Helms-Burton Act (Mar 1996–ongoing).	Cuba			Commentary based on field visits and published sources	**No original research findings**.<br><br>Previous findings are discussed, including documented epidemics of neuropathy due to B-group vitamins deficiency; child intoxication with liquid lye; cases of Guillain-Barré syndrome due to under-chlorinated water. The importance of exemption systems and monitoring of civilian welfare in sanctioned countries is emphasized.		
29.	Barry (2000b)[86]	Embargo by OAS (Oct 1991–Aug 1993) and UN (Jun–Aug 1993, Oct 1993–Oct 1994).	Haiti			Commented abstract	**No original research findings**.<br><br>The article is a brief summary of Gibbons and Garfield (1999), arguing that only an effective exemption enforcement system with continuous monitoring of processes and outcomes can prevent sanctions to damage population health.		
30.	Bastani et al (2021a)[87]	US sanctions (Nov 1979–ongoing), esp.	Iran		Policies to improve the pharmaceutical	Thematic analysis on records of semi-structured	Minimum interview length was 50 minutes. Nine		

	1st Author / year / Journal	Type of econ sanctions	Country focus	Health-related outcomes	Health system outcomes	Methods	Main Findings	Evidence (or suggestion) of modification/mediation	Comments
		measures reintroduced after withdrawal from the Joint Comprehensive Plan of Action (Aug 2018–ongoing).			supply chain under sanctions.	face-to-face interviews with President, Vice-Presidents, managers of Iran's Food and Drug Administration and other experts (Sample: 18 individuals after pilot interviews)	themes and 26 sub-themes were identified.<br><br>The themes were: health policy considerations, including stronger oversight and less interference vis-à-vis private companies, the introduction of clinical guidelines and reform of GP referral system; leveraging on local conditions via public-private partnerships, collaboration with the media and religious authority; cooperation with neighboring countries; higher procurement prices for domestic companies to stimulate import substitution; health insurance reform to target resources on expanding basic coverage; efficiency gains in healthcare by introducing electronic records and prescribing; implementing a tracking and monitoring system for distribution and sale; stronger quality control; better information on alternatives to imported pharmaceuticals for physicians and patients		
31.	Bastani et al (2021b)[88]	US sanctions (Nov 1979–ongoing), esp. measures reintroduced after withdrawal from the Joint Comprehensive Plan of Action (Aug 2018–ongoing).	Iran		Policies to achieve universal health coverage under sanctions.	Thematic analysis on results of 3-round Delphi method (via online survey) from 30 policy themes pre-selected via comparative analysis of 10 countries (Sample: 20 experts in	Comparative analysis generated 30 policy themes, organized in 3 groups. Consensus was reached for 18.<br><br>On "health technology assessment to select essential health services", consensus was achieved on: avoiding premature assessments, consideration of non-economic factors,		Unclear role of the topic of sanctions and associated impacts in the expert consensus process.

	1st Author / year / Journal	Type of econ sanctions	Country focus	Health-related outcomes	Health system outcomes	Methods	Main Findings	Evidence (or suggestion) of modification/mediation	Comments
						healthcare management, health economics and insurance)<br><br>(Sample: 20 individuals)	consideration of cost-effectiveness, potential limits to cost-effectiveness, use of cost-effectiveness in health insurance.<br><br>On "for who to buy interventions and who should receive the most benefits from financing or health services", consensus was achieved on: basic universal coverage, the elderly, pregnant women, the under-18, and low-income as priority groups, targeted demand-side subsidies, regressive effects of unrestricted access to free healthcare in low-income countries, importance of geographic/economic/cultural accessibility.<br><br>On "payment mechanisms" consensus was achieved on: direct payment to providers, tailoring payment to country health insurance system.		
32.	Batmanghelidj and Heydari (2014)[89]	US sanctions (unspecified)	Iran			Commentary based on published sources.	**No original research findings.**<br><br>The authors argue that US tobacco companies are granted a high number of licenses to export to Iran.<br><br>Quoting figures on the prevalence, tax revenue stream, and heath costs of smoking, it is argued that stronger enforcement of border controls against smuggling and higher taxes on tobacco products are needed to improve population health and increase state capacity.		

	1st Author / year / Journal	Type of econ sanctions	Country focus	Health-related outcomes	Health system outcomes	Methods	Main Findings	Evidence (or suggestion) of modification/mediation	Comments
33.	Benjamin et al (2003)[90]	UN sanctions (Aug 1990–May 2003).	Iraq		Operational status of sites, availability of healthcare inputs.	Operational status of sites, availability of healthcare inputs.	Of 12 hospitals surveyed, 11 reported shortages of basic equipment, and 10 drug shortages; only 2 could perform basic resuscitations, and only one could perform intensive care ventilator support, cardiac monitoring and pulse oximetry.<br><br>Child infections were found to be "under reasonable control", although outbreaks in the recent past are interpreted as signs of vulnerability.<br><br>All 8 water, sewage treatment and electricity plants were found to operate "by the flimsiest of means".		Fieldwork is described as assessing "the health effect of economic sanctions", but no attempt is made to documenting this association.
34.	Bessler et al (2004a)[91]					Literature Review	**No original research findings.**		
35.	Bessler et al (2004b)[92]					Literature Review	**No original research findings.**		
36.	Black (1993)[93]	Sanctions by UN, EU and US (May 1991– Jan 2001, with gaps).	Serbia-Montenegro	Cases of tuberculosis, tetanus, weight	Availability of food, pharmaceuticals and medical equipment; health expenditure per capita.	Report based on field observations and interviews.	Field observations include: queues to purchase milk, adequate supply of fresh farm produce in urban markets; a group of refugee mothers and children housed in a hospital's basement; a "clear deterioration" in hospital conditions relative to the previous year.<br><br>Local staff of UN agencies is quoted reporting a large decline in healthcare expenditure; shortage of pharmaceuticals and medical equipment due to war, sanctions and economic decline.<br><br>In different facilities, doctors are quoted reporting suspension of most elective	Currency devaluation; shipment delays due to imperfect exemption system; no exemption on intermediate inputs for pharmaceuticals.	Sanctions are regarded as contributing to declines in population health, together with state breakup, war and counterproductive government policies.

	1st Author / year / Journal	Type of econ sanctions	Country focus	Health-related outcomes	Health system outcomes	Methods	Main Findings	Evidence (or suggestion) of modification/mediation	Comments
							surgery; increases in diagnosed cases of tetanus; shortage and black market circulation of antimicrobials leading to an increase in multi-resistant infections.<br><br>Observations in a psychiatric hospital include a 10-fold decline in recorded drug supplies vis-à-vis before the war; a 90% budget cut in the previous 2 years; an increase in diagnosed TB cases from 2/3 in 1991 to 25 in 1993; lack of soap and tampons; an average 1.9 Kg weight loss apparent from the records for 27 underweight patients; good patients-staff relations.<br><br>The author concludes that "sanctions are clearly damaging the health of the population, affecting the poorest and most vulnerable groups most of all".		
37.	Blacker et al (2007)[23]	UN sanctions (Aug 1990–May 2003).	Iraq	Under-5 mortality rates, birth interval		Descriptive statistics from cross-sectional survey and administrative data.	Estimates of under-5 mortality rates from the Iraq Child and Maternal Mortality Survey of 1999, once computed with the indirect method used by the Iraq population Census of 1997, are comparable to the latter's unadjusted estimates. Non-response is likely to stem from obsolete question format, and should not be adjusted for by assuming it stems from absence of child death in respondent's birth history. If the relevant section of the survey is backdated by a decade, it is comparable with the Census of 1987.		Reply to objections raised against estimates based on ICMMS survey (Ali et al 2003).<br><br>Findings based on UNICEF's ICMMS survey, the reliability of which was later questioned.

	1st Author / year / Journal	Type of econ sanctions	Country focus	Health-related outcomes	Health system outcomes	Methods	Main Findings	Evidence (or suggestion) of modification/mediation	Comments
							The abnormally low ratio of 1-4 to infant mortality in the ICMMS data in the sanction period reflects disproportional increase in infant-specific risks such as low birthweight and decreased exclusive breastfeeding.<br><br>Discrepancy between direct estimates from ICMMS and Census data, and changes in the age composition of under-5 mortality under sanctions, are thus explained without questioning the integrity of the ICMMS.		
38.	Centres for Disease Control (1994)[94]	US embargo (Jul 1963–ongoing), esp. Cuban Democracy Act (Oct 1992–ongoing).	Cuba	Cases of epidemic neuropathy		Report based on Cuban official statistics and case-control studies	**No original research findings**.<br><br>Reported characteristics of the epidemic include: a total number of cases around 50.000; the presence of an optic and a peripheral form; higher incidence among men, in the 45-65 age class, in the tobacco-growing region of Pinar del Rio; eventual remission of symptoms and decrease of incidence after mass administration of B-complex vitamin supplements. Quoted risk factors from case-control studies conducted by Cuban authorities include: low BMI, tobacco smoking, low intake of animal proteins, fat, and sources of B-vitamins.	The discussion section of the report suggests that a causal factor in the condition is B-vitamins deficiency, itself associated with "economic difficulties in Cuba since 1989", for example due to increases in physically demanding commuting induced by fuel scarcity depleting vitamin reserves in the population.	No explicit mention is made of the US embargo.
39.	Chelala (1994)[95]	Embargo by OAS (Oct 1991–Aug 1993) and UN (Jun–Aug	Haiti			Correspondence	**No original research findings**.<br><br>The letter quotes population health indicators for the pre-		

	1st Author / year / Journal	Type of econ sanctions	Country focus	Health-related outcomes	Health system outcomes	Methods	Main Findings	Evidence (or suggestion) of modification/mediation	Comments
		1993, Oct 1993–Oct 1994).					sanction period; argues that HIV/AIDS prevalence has increased under sanctions due to urban violence and unemployment causing internal displacement and high-risk behaviors; and argues that the oil embargo prevents the use of fuel to distribute exempted humanitarian goods.		
40.	Chelala (1996)[96]	US embargo (Jul 1963–ongoing), esp. Cuban Democracy Act (Oct 1992–ongoing).	Cuba			Editorial	**No original research findings**.<br><br>The commentary proposes the relaxing of the US embargo and the organization of diplomatic talks, led by medical practitioners and focused on health issues, as alternatives to the existing US policy on Cuba. Quoting Cuban official statistics, the author expresses the opinion that the US embargo contributes to adverse health outcomes among Cuban citizens.		
41.	Chelala (1998)[97]	US embargo (Jul 1963–ongoing), esp. Cuban Democracy Act (Oct 1992–ongoing) and Helms-Burton Act (Mar 1996–ongoing).	Cuba	Infant and maternal mortality		Short communication	**No original research findings.**<br><br>Figures for infant (7.2 per 1000) and maternal (22 per 100 000) mortality in 1997 are quoted, and emphasis is given to the fact that the values are historical lows for the second consecutive year – despite the recent economic crisis which affected the country.		
42.	Cheraghali (2013)[98]	UN, US, EU sanctions (unspecified)	Iran			Commentary based on published sources.	**No original research findings.**<br><br>Sanctions are deemed responsible of shortages of finished and intermediate pharmaceutical products,		

	1st Author / year / Journal	Type of econ sanctions	Country focus	Health-related outcomes	Health system outcomes	Methods	Main Findings	Evidence (or suggestion) of modification/mediation	Comments
							due to higher transaction costs and restrictions on banking and insurance.		
43.	Choonara (2013)[99]	Iraq: UN sanctions (Aug 1990–May 2003). Cuba: US embargo (Jul 1963–ongoing), esp. Cuban Democracy Act (Oct 1992–ongoing). Palestine: sanctions by UN, US, EU and Russia (unspecified) and blockade by Israel and Egypt (unspecified)	Iraq, Cuba, Palestine			Commentary based on published sources	**No original research findings**.<br><br>For Iraq, published estimates of infant and under-5 mortality estimates under sanctions, and reports of rising prevalence of low-birthweight and malnutrition, are quoted.<br><br>For Cuba, reports of delayed and blocked shipments of medical equipment are mentioned, and good population health in the country is associated to its primary care model.<br><br>For Palestine, reports of shortages of essential medicines and equipment, electricity, and declines in access to potable water, are quoted, with associated evidence of rising anemia among school-age children, prevalence of diarrhea in under-3 children and underweight in infants between 9 to 12 months.<br><br>The author advocates the extension of the Geneva Convention to protect civilians under sanctions.		Discussion partly based on FAO/NRI survey findings (Zaidi and Fawzi, 1995), later partly retracted (Zaidi, 1997), and ICMMS survey, the reliability of which has been questioned.
44.	Cohen (2018)[100]	UN and US sanctions (unspecified)	Korea DPR		Delivery of health inputs as humanitarian aid	Commentary based on UN documents and other published sources	**No original research findings**.<br><br>The author argues that UN and US sanctions contributed to hampering effective humanitarian aid in the country, including the sourcing of medical	Overcompliance with financial sanctions by banks, preventing transactions for exempted items; time costs of licensing monitoring and licensing for exemptions; deterrence of shipping companies due to fear of reputational costs; mobility	

	1st Author / year / Journal	Type of econ sanctions	Country focus	Health-related outcomes	Health system outcomes	Methods	Main Findings	Evidence (or suggestion) of modification/mediation	Comments
							equipment. However, the author concludes that obstructive policies by the North Korean's government play a larger role in preventing basic population health conditions to be achieved in the country.	restrictions on aid workers due to travel ban.	
45.	Coovadia (1999)[101]	UN arms and oil embargo (Nov 1962–May 1994), US sanctions (Oct 1986–Nov 1993)	South Africa			Commentary based on recollection of personal experience and published sources	**No original research findings**.<br><br>While "great controversy" on trade sanctions is said to have occurred, the African National Congress (ANC) position is characterized as supportive, as economic distress was seen as a "necessary condition for liberation". This position was based on the belief that economic growth would not have resulted in the abolition of apartheid, while sanction's adverse impact on black people's incomes was deemed negligible relative to that of continuing segregation.<br><br>The possibility that sanctions worsened the incomes and thus health of the poor, largely black South Africans, is acknowledged, but is seen as exacerbating existing problems, against which little progress was made under previous diplomatic stances; and as a cost to be weighted against the contribution to abolishing apartheid.		
46.	Cotton (1993)[102]	US embargo (Jul 1963–ongoing), esp. Cuban Democracy Act	Cuba	Cases of epidemic neuropathy		Editorial	**No original research findings**.<br><br>The article reports on epidemiological studies on		

	1st Author / year / Journal	Type of econ sanctions	Country focus	Health-related outcomes	Health system outcomes	Methods	Main Findings	Evidence (or suggestion) of modification/mediation	Comments
		(Oct 1992–ongoing).					the epidemic of neuropathy in Cuba – which was by then ongoing. The opinions of various US field investigators and experts are quoted over possible causal factors, including the probable role of nutrition and the potential role of US sanctions in triggering nutritional deprivation in Cuba.		
47.	Cuellar (2015)[103]	US embargo (Jul 1963–ongoing), esp. Cuban Democracy Act (Oct 1992–ongoing) and Helms-Burton Act (Mar 1996–ongoing).	Cuba			Short communication	**No original research findings**.<br><br>The article quotes selected facts about the record of population health, the performance of the healthcare system in Cuba, and argues that sanctions severely restricts access to drugs for chronic conditions.		
48.	Danaei et al (2019)[104]	US sanctions (Nov 1979–ongoing), esp. measures reintroduced after withdrawal from the Joint Comprehensive Plan of Action (Aug 2018–ongoing).	Iran			Correspondence	**No original research findings**.<br><br>Exclusion from international payment circuits is claimed to have slowed down the receipt of international aid during the floods of Spring 2019, requiring the establishment of ad hoc accounts by UNICEF to facilitate inflow of resources from Europe.		
49.	De Vos et al (2012)[105]	US embargo (Jul 1963–ongoing), esp. Cuban Democracy Act (Oct 1992–ongoing).	Cuba	IMR, Incidence of low birthweight and TB, life expectancy.	Public health expenditure, number of various types of health facilities, hospital beds, physician density.	Commentary based on published sources, descriptive statistics and pairwise correlations between them.	**No original research findings.**<br><br>The authors show that, while GDP fell during the *período especial* crisis, public health expenditure, the number of healthcare facilities and professionals all increased. Hospitalizations and hospital beds were constant, before eventually declining in the	The authors claim that "given the importance of GDP decrease, one might have expected a dramatic deterioration of the Cuba's health". This decoupling is deemed due to the fact that "social and health services policies limited social consequences of the crisis" (p. 474).	No discussion of the specific role of sanctions vis-à-vis the Soviet trade slump, and of the relative importance of government policy responses – e.g. economic liberalization vs. public health interventions.

	1st Author / year / Journal	Type of econ sanctions	Country focus	Health-related outcomes	Health system outcomes	Methods	Main Findings	Evidence (or suggestion) of modification/mediation	Comments
							post-crisis years. Previously published figures documenting the limited and temporary extent of health deteriorations (incidence of low birthweight and TB) are cited.		
50.	Dehghani et al (2021)[106]	US sanctions (Nov 1979–ongoing), esp. measures reintroduced after withdrawal from the Joint Comprehensive Plan of Action (Aug 2018–ongoing).	Iran (city of Tabriz, Tabriz University of Medical Science)		Elicited opinions on three guideline questions: "*How have sanctions affected the health research system in Iran?*"; "*Do you have any personal experience about the impact of sanctions on your research projects?*"; "*Which research sections have been most affected by sanctions?*"	Thematic analysis on records of semi-structured interviews with managers, faculty members, postdoctoral and doctoral students (Sample: 24 individuals)	Length of interviews ranged between 31 and 47 minutes. Five themes concerning channels of impact on health research were identified.<br><br>Financial issues, including lower funding due to inflation and changes in budgetary priorities, delays in grant payments and allocation of foreign currency for international payments.<br><br>Difficulties in sourcing laboratory materials and other equipment, due to reluctance of foreign firms leading to higher prices and transaction costs with associated delays, and cash-in-advance terms when contracting Iranian companies; counterfeit or low-quality items; unavailability of maintenance services and spare parts, shortening the lifespan of equipment.<br><br>Stress due to increased workload, including correction of errors due to malfunctioning equipment, and risk of failure of projects.<br><br>Disruption of international collaborations due to increased visa issuing		Sample limited in size and homogeneous institutional affiliation of subjects.

	1st Author / year / Journal	Type of econ sanctions	Country focus	Health-related outcomes	Health system outcomes	Methods	Main Findings	Evidence (or suggestion) of modification/mediation	Comments
							problems, currency devaluation, difficulties in obtaining international scholarships, biased rejection of articles by foreign journals, lack of access to online research resources and software,		
51.	Delamothe (1997)[107]					Editorial	**No original research findings**.<br><br>The article is an editorial quoting published studies on Iraq and Cuba expressing the opinion that doctors should oppose sanctions "whenever these are likely to endanger health"		
52.	Destafkan et al (2020)[108]	(unspecified)	Iran			Commentary based on published literature and legal documents	**No original research findings**.<br><br>Sources of duty in international law that institute accountability for human rights violations due to sanctions include fundamental principles of the UN Charter for the UN Security Council, and the right to health as stated in the WHO constitution, the Covenant on Economic, Social and Cultural Rights and the Convention on the Rights of the Child.<br><br>The cases of Iraq and Cuba illustrate possible health impacts of sanctions, and possible mitigating policies by target stages. In the case of Iran, ambiguous terminology and the treatment of dual-use items in the exemption system, the macroeconomic effects of barriers to oil exports, and the blocking of banking		Discussion partially based on the ICMMS survey, the reliability of which has been questioned.

	1st Author / year / Journal	Type of econ sanctions	Country focus	Health-related outcomes	Health system outcomes	Methods	Main Findings	Evidence (or suggestion) of modification/mediation	Comments
							services associated with financial sanctions, all potentially impact civilians and their human rights.		
53.	Dobson (2000)[109]	UN sanctions (Aug 1990–May 2003).	Iraq			Editorial	**No original research findings**.<br><br>Estimate of under-5 mortality increasing from 56 in 1984-89 to 131 in 1994-99 from unreferenced source.		Source of reported finding not quoted, but likely based on the ICMMS survey, the reliability of which has been questioned.
54.	Drain (2015)[110]	US embargo (Jul 1963–ongoing), esp. Cuban Democracy Act (Oct 1992–ongoing) and Helms-Burton Act (Mar 1996–ongoing).	Cuba			Commentary based on published sources	**No original research findings**.<br><br>The article expresses the opinion that a potential for medical research collaborations exists between Cuba and the US, in fields such as vaccine development, medical education and global health; and that this potential can be realized by lifting the embargo.		
55.	Drain and Barry (2010)[111]	US embargo (Jul 1963–ongoing), esp. Cuban Democracy Act (Oct 1992–ongoing) and Helms-Burton Act (Mar 1996–ongoing).	Cuba			Commentary based on published sources	**No original research findings**.<br><br>The article summarizes information about the impact of the embargo. It states that "during the embargo's first 30 years, Cuban's average life expectancy increased 12.2 years, comparable to Caribbean and South American regions"; that the Cuban Democracy Act "altered the medication supply and likely had focal, serious consequences on Cuban's health"; and that those adverse consequences were temporary, and Cuba's population health continued to improve afterwards.		

	1st Author / year / Journal	Type of econ sanctions	Country focus	Health-related outcomes	Health system outcomes	Methods	Main Findings	Evidence (or suggestion) of modification/mediation	Comments
56.	Dréze and Gazdar (1992)[16]	UN sanctions (Aug 1990–May 2003).	Iraq	Food intake (assessed indirectly through food prices and availability)		Descriptive statistics from household survey; narrative from field observations and interviews.	Between August 1990 and August 1991, food prices increased by a factor of 15 to 20, while nominal earnings stagnated. Food import prices "can be expected to have risen about 25 times" (p. 928). In August 1991, the unskilled monthly real wage was 7% of the previous year level. The cost of the average monthly food basked for a family of six with one infant rose from 66 to 1000 Iraqi Dinars in the same period (800 if food rations are included). The average monthly wage over the same period fell within the range of 120-250 Dinars.<br><br>Food rationing collapsed during wartime. Speaking of their 'hardest' period, households reported giving up meat for staples, eating a single daily meal, consuming 'famine foods', selling household assets, and fights within the family for the allocation of food. Poorer households reported persistent hardship after the war.	The large increase in food prices is deemed due to speculation during wartime; quantity constraints due to *de facto* food embargo until April 1991; the end of subsidized food import prices and the associated currency devaluation due to general sanctions.<br><br>The rationing system is deemed to have been key in preventing starvation in the immediate post-war.	Field observations consistent with other studies.
57.	Dyson (2009)[25]	UN sanctions (Aug 1990–May 2003).	Iraq	Under-5 mortality rates		Descriptive statistics from household surveys and population census	Estimates of under-5 mortality from the Multiple Indicator Cluster Survey of 2006 (MICS3) agree with the Iraq Living Condition Survey of 2004 (ILCS) and fail to show the sharp and prolonged increase depicted by the Iraq Child and Maternal Mortality Survey of 1999 (ICMMS).<br><br>Inspection of first differences of the time series		

	1st Author / year / Journal	Type of econ sanctions	Country focus	Health-related outcomes	Health system outcomes	Methods	Main Findings	Evidence (or suggestion) of modification/mediation	Comments
							reveal agreement in patterns of annual variability across the three surveys, except for the sharp increase after the imposition of sanctions in the ICMMS data, which must be regarded as suspicious.		
58.	Dyson and Cetorelli (2017)[26]	UN sanctions (Aug 1990–May 2003).	Iraq			Commentary based on published literature and survey data	Three surveys (ILCS, MICS3, MICS4) implemented after the 2003 US invasion depict a pattern of under-5 mortality showing no sharp increase after the imposition of sanctions, in contrast with data from the Iraq Child and Maternal Mortality Survey of 1999 (ICMMS). This fact supports the view that the ICMMS was manipulated to inflate mortality estimates for the Centre/South region of the country.<br><br>A temporary increase in mortality in 1991 is likely to have occurred, but of a smaller scale than depicted by the IST survey of Ascherio et al (1992) and the ICMMS.		
59.	Eastman-Abaya (2000)[112]	UN sanctions (Aug 1990–May 2003).	Iraq			Correspondence	**No original research findings**.<br><br>The letter mentions findings from Ali and Shah (2000), and disputes the view that increased mortality is attributable to the Iraqi government rather than to UN sanctions.		Findings quoted are based on UNICEF ICMMS survey, the reliability of which has been questioned
60.	Eisenberg (1997)[113]	US embargo (Jul 1963–ongoing), esp. Helms-Burton Act (Mar 1996–ongoing).	Cuba	Self-inflicted injuries		Editorial quoting published sources.	**No original research findings**.<br><br>The article comments on Andrews et al (1997), linking findings to	Worsening economic conditions leading to outmigration and concomitant US immigration policy change towards Cuba	

	1st Author / year / Journal	Type of econ sanctions	Country focus	Health-related outcomes	Health system outcomes	Methods	Main Findings	Evidence (or suggestion) of modification/mediation	Comments
							worsening economic conditions in Cuba and citing both the collapse of the USSR and the US embargo as contributing factors.		
61.	Fakheran (2019)[114]	US sanctions (Nov 1979–ongoing), esp. measures reintroduced after withdrawal from the Joint Comprehensive Plan of Action (Aug 2018–ongoing).	Iran			Short communication	**No original research findings**.<br><br>The article argues that, in light of evidence on the health effects of sanction and economic crises, the newly re-imposed US sanctions are likely to decrease oral health care use in Iran. As a way to mitigate this impact, the author suggests wider adoption of cost-effective preventive interventions.		
62.	Farmer et al (2003)[115]	US blocking Inter-American Development Bank loan by US veto (2002).	Haiti	Cases of anthrax, polio, drug-resistant TB, and road traffic injuries.	Annual outpatient visits in ambulatory facility	Commentary based on personal experience as field workers and published sources.	The commentary reports on the US vetoing of 164 mln. USD in humanitarian aid at the Inter-American Development Bank, in response to alleged irregularities in the May 2000 parliamentary elections.<br><br>Based on their observation as practicing clinicians, the authors report a steady increase in annual outpatient visits at their ambulatory, from around 10.000 in the first three trimesters of 2001 to 200.000 in the second trimester of 2002 – significantly above the stated nominal staff capacity of 35.000. Unquantified increases in road traffic injuries, the reappearance of polio, anthrax, and drug-resistant TB cases are also noted.	The authors stress the dependence of the country on external humanitarian aid. However, they acknowledge that "the noxious effects of a leaky embargo and the consequences of military rule cannot be disentangled", further noticing the lack of government commitment in the area of health.	

	1st Author / year / Journal	Type of econ sanctions	Country focus	Health-related outcomes	Health system outcomes	Methods	Main Findings	Evidence (or suggestion) of modification/mediation	Comments
63.	Farsad et al (2019)[116]	US sanctions (Nov 1979–ongoing), esp. measures reintroduced after withdrawal from the Joint Comprehensive Plan of Action (Aug 2018–ongoing).	Iran			Statement from professional body	**No original research findings**.<br><br>The article is a statement from the Network of Iranian Nuclear Medicine Scientists. It claims that, despite exemptions for essential drugs, sanctions hamper the provision of intermediate inputs for domestic producers of radiopharmaceuticals. As a consequence, "the activities of Nuclear Medicine Departments are greatly reduced and most probably, in the near future, will be completely stopped".		
64.	Field and Russell (1992)[117]	UN sanctions (Aug 1990–May 2003).	Iraq	Prevalence of malnutrition (underweight, stunting, wasting), anemia, vitamin A deficiency	Food prices, interviews with doctors and mothers	Descriptive statistics from administrative records, interviews and cross-sectional survey	Prevalence of moderate malnutrition at 22% for height-for-age, 20% for weight-for-age, 4% for weight-for-height $(-3 < Z < -2)$; at 18% for mid-upper arm circumference $(135\ mm < X < 125\ mm)$. Prevalence of severe malnutrition at 17% for height-for-age, 6% for weight-for-age, nihil for weight-for-height $(Z < -3)$; at 8% for mid-upper arm circumference $(X < 125\ mm)$.<br><br>Z scores declined with age: from $-.74$ (0-6 months) to -2.04 (above 3 years) for height-for-age; from $-.57$ (0-6 months) to -1.48 (2-3 years) and -1.47 (above 3 years) for weight-for-age. No significant differences across gender and rural/urban lines.	Relying on field interviews of mothers and observations about food prices, the authors write: "the cumulative effect of embargo, war, civil unrest, and sanctions had left poorer people in greater Basrah in a food scarcity and price squeeze, reflecting the country's prior reliance on food imports …. In sum, the child malnutrition documented … reflects a crisis of consumption as well as a crisis of infectious disease" (p. 44)	The absence of a pre-sanction baseline prevents obtaining an impact estimate.<br><br>No adjustment for mortality selection. No random sampling.

	1st Author / year / Journal	Type of econ sanctions	Country focus	Health-related outcomes	Health system outcomes	Methods	Main Findings	Evidence (or suggestion) of modification/mediation	Comments
65.	Frankish (2003)[118]	UN sanctions (Aug 1990–May 2003).	Iraq			Commentary based on published academic ad UN sources	**No original research findings**.<br><br>Declining living standards in Iraq during the 1990s are discussed and linked to war and sanctions, with the latter characterized as impediment to recovery, while the Oil-for-food Programme is taken as a cause of later improvement. Figures on child malnutrition, infant and child mortality, cases of selected diseases, are mentioned and related to reported shortages of energy and spare parts preventing adequate water treatment, sewage and garbage collection.		Extensive mention of sources, but no list of references.
66.	Garfield (1999a)[119]					Literature review	**No original research findings.**		
67.	Garfield (1999b)[120]	Iraq: UN sanctions (Aug 1990–May 2003). Cuba: US embargo (Jul 1963–ongoing), esp. Cuban Democracy Act (Oct 1992–ongoing). Haiti: embargo by OAS (Oct 1991–Aug 1993) and UN (Jun–Aug 1993, Oct 1993–Oct 1994).	Iraq, Cuba, Haiti	Prevalence of low birthweight, child (under-5) mortality rate	Available calories per capita	Commentary based on published sources.	**No original research findings**.<br><br>Sanctions increased in frequency during the 1990s as partial substitute to armed conflict. Technical flaws and politicized implementation led to health and welfare impacts in line with observed trends in civilian exposure to warfare.<br><br>Impacts on health operate through supply constraints on health inputs and macroeconomic effects. Impacts are often greater on children, pregnant women, the elderly and chronically ill. Mitigation policies are available, for example to protect infants, based on prevention and faster		Tabulation of descriptive outcomes as before-after comparisons is provided, but lack of any information on data sources and methodology prevents their consideration as impact estimate.

	1st Author / year / Journal	Type of econ sanctions	Country focus	Health-related outcomes	Health system outcomes	Methods	Main Findings	Evidence (or suggestion) of modification/mediation	Comments
							adoption of low-cost technologies.<br><br>Introducing standards to implement exemption systems and to monitor civilian population in target countries, jointly with greater reliance on targeted sanctions, can enhance the humanitarian potential of sanctions.		
68.	Garfield (2000a)[32]	Iraq: UN sanctions (Aug 1990–May 2003). Cuba: US embargo (Jul 1963–ongoing), esp. Cuban Democracy Act (Oct 1992–ongoing).	Iraq, Cuba			Commentary based on published sources.	**No original research findings.**<br><br>Government policies in Iraq and Cuba are discussed as a contributory factor behind differences in the severity of adverse health consequences of sanctions. In Iraq, which experienced larger, more sustained increases in under-5 mortality, baseline 6-months exclusive breastfeeding was low, and the government introduced infant formula in the rationing system against UNICEF advice.<br><br>Limited decline in malnutrition under the Oil-for-Food Programme is related to Iraq's curative healthcare system model, with few staff trained in primary care and community health. The 'pro-curative bias' was reflected in the use of Programme funds.<br><br>In Cuba, secular decline in infant and under-5 mortality was sustained under sanctions, despite economic decline and declines in child and maternal nutrition.	Different policy responses are associated to differences in health system models, despite other similarities in terms of form of government and trade structure.<br><br>It is recognized, however, that differences in outcomes also reflect the greater damage inflicted by armed conflict and sanctions in Iraq.	Discussion partly based on findings from ICMMS survey, the reliability of which has been questioned.

	1st Author / year / Journal	Type of econ sanctions	Country focus	Health-related outcomes	Health system outcomes	Methods	Main Findings	Evidence (or suggestion) of modification/mediation	Comments
							Government responses included promotion of breast-feeding, targeted food supplementation for mothers and children, health promotion activities.		
69.	Garfield (2000b)[27]	UN sanctions (Aug 1990–May 2003).	Iraq	Prevalence of malnutrition (underweight, stunting, wasting)		Descriptive statistics based on survey data.	According to 5 nationally representative or nearly-representative surveys the prevalence of malnutrition went from 9.2 (underweight), 3 (stunting) and 18.7 (wasting) in 1991 to 23.4, 11 and 31.2 in 1996; 24.7, 8.9, 27.5 in 1997; 21.8, 8.3, 26.5 in 1998; 21, 8.5, 20.1 in 1999. The comparison suggests rapid increase in malnutrition under sanctions, and little decrease after the implementation of exemptions under the Oil-for-Food Programme.	Increase in food prices due to decline in food imports. Low level of exclusive breastfeeding.	Comparison from repeated cross-sections with potential lack of comparability.<br><br>No baseline to estimate the impact of armed conflict and initial imposition of sanctions, and no clear baseline for the implementation of the Oil-for-Food Programme
70.	Garfield (2001b)[121]	Sanctions by UN, EU and US (May 1991– Jan 2001, with gaps).	Serbia-Montenegro			Brief communication	**No original research findings**.<br><br>The article summarizes main findings of Garfield (2001a).		
71.	Garfield (2002)[122]	Iraq: UN sanctions (Aug 1990–May 2003). Cuba: US embargo (Jul 1963–ongoing), esp. Cuban Democracy Act (Oct 1992–ongoing). Haiti: embargo by OAS (Oct 1991–Aug 1993) and UN (Jun–Aug 1993, Oct 1993–Oct 1994). Serbia-Montenegro: sanctions by UN, EU and US (May	Iraq, Cuba, Haiti, Serbia-Montenegro			Commentary based on published sources.	**No original research findings**.<br><br>Reliance upon sanctions increased after the Cold War due to greater potential effectiveness and lower perceived costs vis-à-vis open war. Evidence was gathered that in key episodes, sanctions adversely affect the health of populations in sanctioned countries, casting doubt on their legitimacy in a UN framework and with respect to international law customs. The presence of exemption clauses does not guarantee		

	1st Author / year / Journal	Type of econ sanctions	Country focus	Health-related outcomes	Health system outcomes	Methods	Main Findings	Evidence (or suggestion) of modification/mediation	Comments
		1991– Jan 2001, with gaps).					that civilians will not be affected.<br><br>The literature suggests rising mortality and morbidity, with greater effect on heavily import-dependent countries and for more comprehensive and multilateral sanctions – with Iraq as a leading case. Responses to sanctions by governments and households in sanctioned countries can mediate the effect, Cuba being the major example of successful mitigation.<br><br>Prevention of these adverse effects on health requires baseline data collection and follow-up, preparedness to timely identify vulnerabilities based on previous experiences, development of codified procedures for effective exemption systems.		
72.	Garfield et al (1995)[123]					Literature review	**No original research findings.**		
73.	Garfield et al (1997)[124]	UN sanctions (Aug 1990–May 2003).	Iraq	Case fatality of burn injuries, postoperative infection rate.	Operational status of sites, availability of healthcare inputs.	Field observations in selected hospitals (20% of Iraq's civilian hospital beds).	Approximately one third of hospital beds visited were closed. Average length of stay "more than halved" relative to pre-war period. Observations in hospitals included: patients bringing their own blankets, kerosene and electric stoves; leaking sewage pipes; in a hospital's radiology "expiry date of the barium they were using was 1990". Reported scarcity of disinfectants and antiseptics, spare parts for diagnostic and therapeutic equipment, anesthetics and surgical materials, wound dressings,		Authors report that most information reported could not be verified.

	1st Author / year / Journal	Type of econ sanctions	Country focus	Health-related outcomes	Health system outcomes	Methods	Main Findings	Evidence (or suggestion) of modification/mediation	Comments
							burn ointment, antibiotics, inhalers for asthma patients, materials for X-rays.<br><br>Increase in case fatality for burns covering between 40% and 70% of skin surface (one hospital). Postoperative infection rate in patients with clean wounds rose from 5% to 25-30% (some hospitals).<br><br>The authors conclude that their experience "suggest a remarkable decline in what was until recently a medically advanced country".		
74.	Ghalibafian et al (2018)[125]	US sanctions (Nov 1979–ongoing), esp. measures reintroduced after withdrawal from the Joint Comprehensive Plan of Action (Aug 2018–ongoing).	Iran			Short communication.	**No original research findings**.<br><br>The article reports drug scarcities from a pediatric hospital in Teheran. Drugs to treat leukemia are reported to be out of a month stock, while other cancer drugs and antimicrobials are reported to be on a 3-month stock.<br><br>The authors report that "during the previous embargo, radiation treatments in our hospital were interrupted for 2 months, until spare parts could be imported".		
75.	Gharebaghi and Heidary (2020)[126]	US and EU sanctions (unspecified)	Iran			Opinion piece	**No original research findings**.<br><br>The article comments on salient early phases of the COVID-19 outbreak in Iran, expressing the opinion that "the important issue is lifting sanctions against Iran, at least in the area of lifesaving		

	1st Author / year / Journal	Type of econ sanctions	Country focus	Health-related outcomes	Health system outcomes	Methods	Main Findings	Evidence (or suggestion) of modification/mediation	Comments
							medical supplies, which could decrease the extent of the tragedy and help prevent a second wave of the disease".		
76.	Gibbons and Garfield (1999)[127]	Embargo by OAS (Oct 1991–Aug 1993) and UN (Jun–Aug 1993, Oct 1993–Oct 1994).	Haiti	Prevalence of moderate-to-severe malnutrition (unspecified, wasting); IMR, 1-4 mortality rate.		Descriptive statistics from health facilities, household surveys and published literature (Sample: epidemiological surveillance, 42 facilities; nutritional surveys: unspecified).<br><br>Observations and interviews with affected women, governmental representatives, diplomats, relief workers.	Under-5 prevalence of moderate-to-severe malnutrition in surveillance stations rose from 18% in 1993 to 24% in September 1994, while according to nationally representative surveys it rose from 3.4% in 1990 to 7.8% in 1994-5.<br><br>Between 1987 and 1994 IMR declined from 101 to 74 per 1000, while 1-4 mortality rose from 56 to 61.<br><br>The following coping strategies were observed among large section of the population: urban-to-rural migration to seek cheaper food, reliance upon remittances, dissaving and sale or household assets, co-habitation, informal sector employment, black-market, prostitution, changed dietary habits, increased informal unions among couples, decreased school attendance, and indentured servitude among children.	Change in assembly industry employment declined from -7.5% 1986-1991 to -80% in 1991-1994.<br><br>GDP per capita decreased by 30% in the 3 years following the coup.<br><br>The price of rice and corn rose 137% and 184% respectively between September 1991 and September 1994. The price of infant formula increased 283% from November 1991 to September 1994.<br><br>Fuel shortages, closure of health facilities and severing of ties between international agencies and public sector staff caused a decline in measles immunization coverage, and a measles epidemic from June 1991 to November 1993.<br><br>In the capital Port-au-Prince, access to potable water declined from 53% in 1990 to 35% in 1994.	Survey-based estimates for the prevalence of malnutrition (wasting) and 1-to-4 mortality already employed in Mulder-Sibanda (1998).<br><br>The comparison between surveillance-based and survey prevalence estimates for undernutrition suggest that the former sample is selective on child nutritional status.
77.	Gorji (2013)[128]	Sanctions by the US, UN and EU before the Joint Comprehensive Plan of Action (unspecified).	Iran			Correspondence	**No original research findings**.<br><br>The article is a letter arguing that, despite formal exemptions, sanctions against Iran hampered the delivery and manufacture of pharmaceuticals for chronic conditions that affect about 6 million people, by blocking		Discussion partly based on findings from ICMMS survey, the reliability of which has been questioned.

	1st Author / year / Journal	Type of econ sanctions	Country focus	Health-related outcomes	Health system outcomes	Methods	Main Findings	Evidence (or suggestion) of modification/mediation	Comments
							trading services such as licensing and shipping, and by general deterrence of foreign companies.		
78.	Gorji (2014)[129]	Sanctions by the US, UN and EU before the Joint Comprehensive Plan of Action (unspecified).	Iran			Correspondence	**No original research findings**.<br><br>(*same as previous entry*)		Discussion partly based on findings from ICMMS survey, the reliability of which has been questioned.<br><br>Wrong excess death estimate (300.000 instead of 46.900) attributed to Ascherio et al (1992)
79.	Habibzadeh (2016)[130]	US sanctions (unspecified)	Iran		Access to medical education	Correspondence	**No original research findings**.<br><br>The letter reports the case of an Iran-based medical student who was denied enrolment to an online medical education course by a US university, reportedly due to the institution's need to comply with US sanctions.		
80.	Harvard Study Team (1991)[15]	UN sanctions (Aug 1990–May 2003).	Iraq	Prevalence and incidence of child infections, malnutrition	Energy supply, operational status of visited sites, availability of selected healthcare inputs	Field visits to healthcare facilities, power plants, water and sewage treatment plants, on-site interviews, site-level administrative data.	In April-May 1991, prevalence of severe gastroenteritis among hospitalized children was 91% (Irbil), 78% (Kirkuk), 84% (Sulaymaniyah), and 38% (Baghdad). For the latter site, incidence increased from 17% to 35% relative to previous year. Prevalence of child malnutrition was found to be 32% (Baghdad), 57% (Irbil), 52% (Kirkuk) and 48% (Sulaymaniyah). Reported two- to three-fold increase in hospital mortality.<br><br>In health centers, consultations for	Destruction of energy generation capacity by bombings (23% of pre-war level in May 1991.) and shutdown or destruction of water and sewage treatment systems, leading to polluted water supply.<br><br>Both armed conflict (including post-war civilian uprising) and sanctions are mentioned as upstream causes.	

	1st Author / year / Journal	Type of econ sanctions	Country focus	Health-related outcomes	Health system outcomes	Methods	Main Findings	Evidence (or suggestion) of modification/mediation	Comments
							gastroenteritis rose from 4 to 42 per day relative to previous year (Kirkuk). The share of patients with gastroenteritis rose from 18% to 55% in Kirkuk, from 3.6% to 17.7% in Baghdad.<br><br>Visited hospitals (16) reported complete or partial lack of running water (8), major sanitation problems (11), electrical failures (5). Local authorities reported closure of 14 of 19 health centers in Basra, 37 of 42 in Irbil, 14 of 20 in Sulaymaniyah.<br><br>All hospitals reported lack of laboratory reagents, materials for radiology, damage to associated equipment. All facilities reported drug shortages, allegedly started in the second half of 1990, including antimicrobials, anti-parasitic, anesthetics, vaccines, intravenous fluids, insulin.		
81.	Heidari et al (2017)[131]	UN sanctions (Jul 2006–Jan 2016), esp. UNSC Resolution 2231 (Jan 2016).	Iran			Short communication.	**No original research findings**.<br><br>The article refers changes in per capita use of the blood-clotting protein factor VIII, a treatment for hemophilia: mean per capita use was 1.6 international units (UI) before 2006; it decreased to 0.5 UI after sanctions were imposed, to then increase again at 2.7 when sanctions were lifted.		The numbers given are not clearly presented and related to the timing of sanctions.
82.	Hosseini (2013)[132]	Sanctions by the US, UN and EU before the Joint	Iran			Report based on official data, government	Imported products constitute less than 4% of pharmaceutical sector	Shortages caused by higher transaction costs and administrative delays in	No date on the figure for import shares of volume and value of

	1st Author / year / Journal	Type of econ sanctions	Country focus	Health-related outcomes	Health system outcomes	Methods	Main Findings	Evidence (or suggestion) of modification/mediation	Comments
		Comprehensive Plan of Action (unspecified).				reports and published literature	output, but ~40% of its value. These include anesthetics, plasma derived anti-coagulants, treatments for cancers, emergency hypertension, heart failure.<br><br>The number of pharmaceuticals reported as being in short supply increased from 88-78 in July-September 2012 to 147-160 in October-November, not falling below 120 for the whole period (ending September 2013).<br><br>An SMS-based system providing public information on local drug availability receives approximately 20 000 monthly queries.	approving international payments; overcompliance by international firms, fearing US prosecution despite licensing.	pharmaceutical output.
83.	Huertas et al (1996)[133]	US embargo (Jul 1963–ongoing), esp. Cuban Democracy Act (Oct 1992–ongoing)	Cuba			Correspondence	**No original research findings**.<br><br>Enrique Huertas argues that, to achieve desirable political improvements in Cuba, the US embargo is needed and should not be relaxed.<br><br>G. E. Martin argues that adverse health outcomes in Cuba are due to Cuba's collectivist agricultural policies and excessive military spending, not to the US embargo, which does not prevent Cuba from importing medical supplies from non-US companies such as those in Europe and Japan.<br><br>Anthony F. Kirkpatrick argues that the US embargo restricts access to pharmaceuticals for Cuba,		Comments on Chelala (1996)

	1st Author / year / Journal	Type of econ sanctions	Country focus	Health-related outcomes	Health system outcomes	Methods	Main Findings	Evidence (or suggestion) of modification/mediation	Comments
							pointing out that half of newly patented pharmaceuticals that attained world-class status after 1975 are US-made; quoting the case of 1 year waiting for an export licence to Cuba by a Belgian subsidiary of a US company; the denial of a licence for x-ray replacement parts; and mentions WHO sources reporting higher prices for Cuba by non-US vis-à-vis US companies, suggesting higher costs, or reluctance to give prices due to fear of reprisals by the US government.<br><br>Chelala replies stressing the multiplicity of factors involved in Cuba's health problems, of which the US embargo is a significant one.		
84.	Kandela (1997a)[134]	UN sanctions (Aug 1990–May 2003).	Iraq			Editorial	**No original research findings**.<br><br>Salient observations in the narrative include shortages of pharmaceutical and medical equipment, supplementary employment among medical professionals, deteriorating quality of medical education due to lack of access to international literature, rise in infections due to disrupted garbage collection.		
85.	Kandela (1997b)[135]	UN sanctions (Aug 1990–May 2003).	Iraq			Editorial	**No original research findings**.<br><br>The article mentions the release of a report by Iraqi government claiming 800 000 deaths attributable to sanctions since their		The mentioned government source is not referenced.

	1st Author / year / Journal	Type of econ sanctions	Country focus	Health-related outcomes	Health system outcomes	Methods	Main Findings	Evidence (or suggestion) of modification/mediation	Comments
							imposition. The opinion of Iraqi health authorities is referred that, besides the lifting of sanctions, allowance of medical tourism by air travel would improve the situation.		
86.	Karimi and Turkamani (2021)[136]	US sanctions (Nov 1979–ongoing), esp. measures reintroduced after withdrawal from the Joint Comprehensive Plan of Action (Aug 2018–ongoing).	Iran			Short communication	**No original research findings.**<br><br>In the context of the current COVID-19 pandemic, sanctions are regarded as limiting imports and causing shortages or ventilators, protection and other exempted medical equipment, due to lack of access to financing facilities.		
87.	Keck and Reed (2012)[137]	US embargo (Jul 1963–ongoing), esp. Cuban Democracy Act (Oct 1992–ongoing) and Helms-Burton Act (Mar 1996–ongoing).	Cuba			Commentary based on published sources.	**No original research findings**.<br><br>Healthcare policies in post-revolutionary Cuba are described, emphasizing the role of community-level primary health care.<br><br>Together with the collapse of the USSR, the Cuban Democracy Act is mentioned as a factor behind a temporary setback in population health. The authors stress continued policy commitment to population health under sanctions.		
88.	Khanal et al (2016)[138]	India-Nepal border blockade (Sep 2015–Feb 2016)	Nepal			Correspondence	**No original research findings**.<br><br>The letter expresses concerns over the cumulative effects of two earthquakes and a blockade at the Indian Border for population health in Nepal, which is imposing delays on		

	1st Author / year / Journal	Type of econ sanctions	Country focus	Health-related outcomes	Health system outcomes	Methods	Main Findings	Evidence (or suggestion) of modification/mediation	Comments
							imports of fuel and pharmaceuticals. A reply by Richard Garfield stresses the known potential for mobilizing internal resources to ensure primary healthcare under sanctions, even at low income levels.		
89.	Kheirandish et al (2015a)[139]	US, EU and UN sanctions targeting Iran's banking system (unspecified)	Iran		Media-reported access to pharmaceutical	Descriptive statistics from time series of news media items.	Out of 371 included news media items, those classified as reporting a shortage were 145 (39%); those classified as raising concerns about availability without reporting a shortage were 129 (35%); and those classified as reporting no shortages or improved availability were 97 (26%).<br><br>The number of relevant media reports increased throughout the observation period (2011-13).	Despite the acknowledged possibility of reporting bias, the Increase in reported shortages and concerns is deemed to be accurate and reflect shortages caused by sanctions.	No clear exposure period or variable is related to the outcome data presented.<br><br>No search words and screening statistics are presented.
90.	Kheirandish et al (2015b)[140]					Literature review	**No original research findings**.		
91.	Kirkpatrick (1996)[141]	US embargo (Jul 1963–ongoing), esp. Cuban Democracy Act (Oct 1992–ongoing)	Cuba		Non-legal determinants of the availability of medicinal products	Report based on oral and written interviews with civil servants and staff of involved companies in 1993-1996	On-site inspections prescribed by the exemption system are reported to raise transaction costs for US companies willing to export on Cuba. Companies also report administrative difficulties in obtaining licenses, and very serious legal penalties for terms violations. The risk of retaliation due to US extra-territorial clauses is reported to have raised export prices of non-US companies.		
92.	Kirkpatrick (1997)[142]	US embargo (Jul 1963–ongoing), esp. Cuban Democracy Act (Oct 1992–ongoing)	Cuba			Commentary based on published sources	**No original research findings**.<br><br>The article counters various claims made by the US State Department about the health effects of the US embargo.		

	1st Author / year / Journal	Type of econ sanctions	Country focus	Health-related outcomes	Health system outcomes	Methods	Main Findings	Evidence (or suggestion) of modification/mediation	Comments
							In particular, it is claimed that, due to implementation factors, exemptions for essentials fail to prevent significant additional costs – current and expected, monetary and non-monetary – for US companies and subsidiaries willing to trade in food and drugs with Cuba.		
93.	Kokabisaghi (2018)[143]		Iran			Literature review	**No original research findings.**		
94.	Kokabisaghi et al (2019)[144]	UN sanctions (Jul 2006–Jan 2016).	Iran		Share of research publications with foreign collaborator, annual number of publications	Pairwise correlation and Mann-Kendal trend test on time series of bibliometric indices.	Between 2010 and 2017, the share of manuscript with international collaborators increased, while it was declining since 1996. An insignificant correlation with GDP is interpreted as rejecting the hypothesis that the increase is only caused by efforts to access foreign funds due to declining domestic resources under sanctions. A statistically significant positive trend was also detected in annual number of publications in 1996-2018, but was reported to be absent for annual visibility of publications.		No definition of exposure period. Analysis of the determinants of international collaborations limited to univariate association. GDP likely a poor proxy for domestic research funds.
95.	Kumar (1998a)[145]	US sanctions against India (May–Nov 1998)	India			Short communications	**No original research findings**.<br><br>Published in short sequence, the articles report on announced increases in public expenditure for health and welfare, but also for military purposes, after the imposition of sanctions; and on the deferred decision on three World Bank loans, eventually approved “thanks to a liberal interpretation of these loans as humanitarian aid” (Kumar, 1998b).		The figures quoted for public spending are all percentages, are thus insufficient to evaluate the real nature of these changes. It is also unclear whether they can be plausibly interpreted as a response to the sanction themselves.<br><br>If accurate, the reported event highlights the role of

	1st Author / year / Journal	Type of econ sanctions	Country focus	Health-related outcomes	Health system outcomes	Methods	Main Findings	Evidence (or suggestion) of modification/mediation	Comments
									definitions of concepts such as "humanitarian" and "essential" in creating room for bargaining over exemptions, and thus variation in the severity of sanctions.
96.	Kumar (1998b)[146]						*See previous entry*		
97.	Kuntz (1994)[147]	US embargo (Jul 1963–ongoing), esp. Cuban Democracy Act (Oct 1992–ongoing)	Cuba		Availability and price of food, basic commodities, pharmaceuticals and medical equipment.	Report based on field observations, interviews with government and UN officials, health workers, ordinary citizens.	The synergistic impact of the Soviet trade slump and the US embargo led to rising import prices and shortages of hard currency, food, fuel, intermediate inputs for farming and pharmaceuticals, and medical equipment.<br><br>Extensive adjustments at all levels of society and government, including food and healthcare rationing and promotion of breastfeeding, ensured adequate diet and healthcare provision. However, evidence of rising incidence of common infections, anemia and low-birthweight children suggests a vulnerable situation and limited room for further coping.	Higher import prices due to higher transport costs; higher mark-ups; greater quality risks and inventory costs associated to larger shipments; licensing costs and retaliation risks faced by foreign companies.	No description of ethnographic context; only narrative reporting of observations.<br><br>Concerning the relative contribution of sanctions and the Soviet trade slump on health outcomes, the authors write that "the embargo is not the only, or perhaps even the major, factor".
98.	Lafta and Al-Nuaimi (2019)[148]	UN sanctions (Aug 1990–May 2003).	Iraq			Literature review	**No original research findings.**		Discussion partly based on FAO/NRI survey findings (Zaidi and Fawzi, 1995), later partly retracted (Zaidi, 1997), and ICMMS survey, the reliability of which has been questioned.
99.	Larijani (2016)[149]	Sanctions by the US, UN and EU before the Joint Comprehensive	Iran			Commentary based on published sources and official stratistics.	**No original research findings**.<br><br>Unlike other non-communicable diseases,		

	1st Author / year / Journal	Type of econ sanctions	Country focus	Health-related outcomes	Health system outcomes	Methods	Main Findings	Evidence (or suggestion) of modification/mediation	Comments
		Plan of Action (unspecified).					diabetes care was not substantially impacted by sanctions.<br><br>Higher transaction costs and currency devaluation increased import prices, consistent with observed increases in pharmaceutical expenditure in 2009-2013. However, unlike chemotherapy and immunosuppressants, diabetes medications were domestically produced, and local firms bypassed financial sanctions, ensuring continuing supply of intermediate inputs.<br><br>Lifting of sanctions might facilitate transfer of medical technology, but also increase population exposure to dietary risks.		
100.	Lee and Haines (1991)[150]	UN sanctions (Aug 1990–May 2003).	Iraq			Commentary based on published sources	**No original research findings**.<br><br>Having reviewed available evidence on post-war trends in mortality and risk factors, the authors conjecture that the share of civilian deaths will increase relative to the figure based only on direct warfare mortality.		
101.	Legetic et al (1996)[151]	Sanctions by UN, EU and US (May 1991– Jan 2001, with gaps).	Serbia-Montenegro		Hospitalization rate, annual primary healthcare visits	Descriptive statistics and uncontrolled comparisons from administrative data and cross-sectional household surveys	Between 1990 and 1992, in Serbia hospitalizations per 1000 population declined by 15.68, annual primary healthcare visits per capita declined 3.52 to 2.07 for adults, 1.36 to 0.88 for women and 6.5 to 4.25 for children.<br><br>Under sanctions (1994), in the two surveyed regions,		Lack of pre-sanction baseline prevents obtaining an impact estimate.

	1st Author / year / Journal	Type of econ sanctions	Country focus	Health-related outcomes	Health system outcomes	Methods	Main Findings	Evidence (or suggestion) of modification/mediation	Comments
							87.3% and 98.5% of households lived within 4km to the closest health facility, but 80% and 81.4% of households reported difficulties in obtaining some type of care, including treatment, diagnostics, emergency services, and surgery. Main reported causes of difficulties were: lack of materials, lack of financial resources, lack of proximate facility.		
102.	Lyme (2012)[152]	Sanctions by US (Dec 2003 – ongoing), esp. Executive Order 13527 (Apr 2011) and followings; EU (May 2011– ongoing); League of Arab States (Nov 2011); Turkey (unspecified)	Syria		Prices and availability of essential goods, including food and pharmaceuticals	Qualitative analysis based on official documents, published sources, and interviews with diplomats, international and national development aid workers, and academics at distance or during field visits in Brussels, Damascus, Beirut and Ankara	Quoting primary sources, sanctions are deemed a contributing factor in documented shortages of medical equipment and pharmaceuticals, and in raising the price of basic staples like wheat.<br><br>Moreover, sanctions are thought to have contributed to a contraction of economic activity and higher inflation, leading to lower incomes in the general population.	Overcompliance with financial sanctions raising import costs of (otherwise exempted) food and medical items; trade sanctions leading to lower macroeconomic activity and higher inflation due to higher energy prices and lower export demand.	Both the direct and macroeconomic effect of sanctions are confounded by the effects of armed conflict and modified by governmental decisions. In particular, “the indirect effects of the sanctions are highly intertwined with the conflict’s direct repercussions and thus highly difficult to isolate” (59)
103.	Madani-Lavassani (2020)[153]	US sanctions (Nov 1979– ongoing), esp. measures reintroduced after withdrawal from the Joint Comprehensive Plan of Action (Aug 2018– ongoing).	Iran			Commentary based on published sources.	**No original research findings**.<br><br>Past episodes like Iraq show that sanctions can have adverse impacts on population health, and motivate assessment of current episodes, including Iran.<br><br>Ambiguity of key terms in exemption systems create uncertainty that deters legitimate trade of exempted items. These effects, in addition to the ban from		Discussion partially based on the ICMMS survey, the reliability of which has been questioned.

	1st Author / year / Journal	Type of econ sanctions	Country focus	Health-related outcomes	Health system outcomes	Methods	Main Findings	Evidence (or suggestion) of modification/mediation	Comments
							international banking infrastructure, are deemed responsible of documented shortages of pharmaceuticals, medical equipment, and obstacles to international collaborations in medical research. Some reports focus on the impact of shortages of medication for hemophilia, anesthetics, and asthma on children.<br><br>However, rigorous evidence on the causes of shortages is limited, and possible alternative factors must be considered, including misplaced government policies and biased reporting.<br><br>International effort to improve the exemption system is advocated		
104.	Marks (1999)[154]					Commentary	**No original research findings**.<br><br>Legal aspects of sanctions are discussed in light of the evidence of adverse health impacts and low success rate. This body of evidence disputes both the effectiveness and 'proportionality principle' of sanctions – whereby goals have to be measured expected undesirable side-effects.<br><br>Neither the UN nor states have direct legal responsibility to protect foreigners who reside in their own countries from threats to their human rights stemming from sanctions.		

	1st Author / year / Journal	Type of econ sanctions	Country focus	Health-related outcomes	Health system outcomes	Methods	Main Findings	Evidence (or suggestion) of modification/mediation	Comments
							Responsibility also implies wilful intent, which is not easy to establish. Nevertheless, The evidence does point to sanctions being conductive to human rights violations, and steps are being made by some international actors to reform the instrument – consistent with some assumption of responsibility.		
105.	Massoumi and Koduri (2015)[155]	Sanctions by the US, UN and EU before the Joint Comprehensive Plan of Action (unspecified).	Iran			Commentary based on published sources and interviews to medical professionals	**No original research findings**.<br><br>Sanctions are held responsible of shortages of pharmaceuticals and medical equipment, with reallocation to alternative import markets associated with declines in product quality and limited by the exclusive holding of licenses of US and European patented product by US and European manufacturers.<br><br>Quoted opinions of interviewed doctors include: the role of restricted international payments in causing drug shortages; lower quality of suturing material imported from non-sanctioning countries and manufactured domestically; reliance upon cheaper but older and riskier products; shortages of chemotherapy, anaesthetics, and PET-scanners.	Restrictions on international payments and overcompliance by foreign companies.	
106.	Maziak et al (2013a)[156]	(unspecified)	Syria			Correspondence	**No original research findings.**<br><br>Comment on Sen et al (2013). The authors criticize the study for presenting “a skewed analysis of threats to		

	1st Author / year / Journal	Type of econ sanctions	Country focus	Health-related outcomes	Health system outcomes	Methods	Main Findings	Evidence (or suggestion) of modification/mediation	Comments
							the health and wellbeing of Syrians", as the designation of sanctions as the cause of adverse outcomes mentioned in the article (currency devaluation, scarcity of electricity and essential drugs) is confounded by "a situation of all-out civil war". The targeting of healthcare facilities and workers by government military forces is mentioned as an overlooked factor.		
107.	Maziak et al (2013b)[157]	(unspecified)	Syria			Correspondence	**No original research findings.**<br><br>Correspondence on Al Faisal et al (2012a). The authors criticize the article claiming that no evidence and plausible mechanism is provided to ascribe a causal role to sanctions in the deterioration of population health in Syria. The omission of the role of Syrian government military forces is criticized.		
108.	McCarthy (2000)[158]					Short communication	**No original research findings**.<br><br>The article is a brief summary of Morin and Miles (2000).		
109.	Mehtarpour et al (2020)[159]	US sanctions (Nov 1979–ongoing), esp. measures reintroduced after withdrawal from the Joint Comprehensive Plan of Action (Aug 2018–ongoing).	Iran		Elicited opinion on 5 guideline questions: "*What do you think are the important international and external determinants that have influenced antimicrobial resistance in our country?*"; "*How did these factors*	Thematic analysis on records of semi-structured face-to-face interviews with policymakers, managers and academics from 7 institutions working on antimicrobial resistance	Average length of interviews was 55 minutes. Interviewees were 13 men and 11 women.<br><br>Seven sub-themes were identified on the two themes of international factors facilitating or hampering the control of antimicrobial resistance.		

	1st Author / year / Journal	Type of econ sanctions	Country focus	Health-related outcomes	Health system outcomes	Methods	Main Findings	Evidence (or suggestion) of modification/mediation	Comments
					affect (decrease or increase) AMR?"; "Have there been any that influenced the policies developed to combat AMR?"; "*Has it been positive or helpful or has it had a detrimental effect on the policies that have been formed? How?*"; "*Which international actors and stakeholders have had a positive or negative impact on the AMR in our country? How?*"	(Sample: 24 individuals)	Barriers to antimicrobial resistance attributed to sanctions include: a decline in international purchasing power to import laboratory supplies, and higher transaction costs on international orders; increased production, smuggling, and black-market supply of low-quality and counterfeit equipment, raw materials, and pharmaceuticals; higher production costs in meat production, incentivizing antimicrobials overuse in combination with government price controls; macroeconomic decline exerting pressure on safety standards; lower funding for control activities and research, in part due to de-prioritized prevention, disruption of international collaborations.		
110.	Mohammadi (2013)[160]	(unspecified)	Iran			Short communication	**No original research findings**.<br><br>The article reports opinions by Iranian professionals, including a former Health Minister, on the increase in prices and decrease in quantity of drugs for many chronic conditions. It is claimed that, while Iran produces 90% of its drugs domestically, sanctions impede provision of intermediate inputs.		
111.	Moret (2014)[161]	(unspecified)	Iran, Syria			Report based on interviews with EU, UN, and EU member state officials, analysis of EU documents,	UN officials involved in aid delivery to Syria quoted stating that EU sanctions made it "more complicated, often more expensive … to procure hardware and		Association between sanctions and adverse health outcomes mostly based on analogy from past

	1st Author / year / Journal	Type of econ sanctions	Country focus	Health-related outcomes	Health system outcomes	Methods	Main Findings	Evidence (or suggestion) of modification/mediation	Comments
						and published sources	supplies for essential services such as medical equipment and drugs".		episodes and media sources.
112.	Morin and Miles (2000)[162]					Editorial	**No original research findings.**<br><br>Statement by the American College of Physicians – American Society of Internal Medicine. The statement recognizes difficulties in attributing adverse health impacts to sanctions; interprets existing studies as plausible evidence of morbidity and mortality that is preventable by modifying or lifting sanctions; suggests that harmful sanctions are contrary to the spirit of the International Covenant on Economic, Social and Cultural Rights; that law of warfare should apply to sanctions, including prohibition to target medical facilities and to obstruct supply of food and medicines.<br><br>The statement supports the establishment of exemption systems, their management by competent third parties, the active provision of health-related goods if required, and continuous monitoring and assessment of sanction regimes.		Discussion partly based on FAO/NRI survey findings (Zaidi and Fawzi, 1995), later partly retracted (Zaidi, 1997).
113.	Namazi (2013)[163]	(unspecified)	Iran		Availability of pharmaceuticals and medical equipment	Interviews with import-export operators, manufacturers and distributors of pharmaceuticals and medical equipment.	Sanctions lead to import shortages in these health inputs due to limited availability of trade services and of international currency.<br><br>Diversion to China and India in addition to existing	Sanctions led to the exclusion of major Iranian trade banks from the Interbank Financial Telecommunication Society. The lack of legal certainty over exempted items and high penalties associated to violations deter many	Only narrative reporting of qualitative findings.

	1st Author / year / Journal	Type of econ sanctions	Country focus	Health-related outcomes	Health system outcomes	Methods	Main Findings	Evidence (or suggestion) of modification/mediation	Comments
							volumes is unfeasible for the patented products produced only by Western companies, or prohibited by restrictions on input quality for domestic producers operating under licencing agreements with Western companies. When diversion is feasible, anecdotal evidence suggests reduced product quality (lower effectiveness, greater side-consequences).<br><br>Illegal smuggling and black market sales of drugs has emerged, increasing the circulation of counterfeited, damaged or expired medicinal products.<br>The authors conclude that the "pronounced role of sanctions in creating shortages of life-saving medical supplies and drugs in Iran may have been unintentional, but it is also irrefutable" (7).	Western providers of banking, insurance and shipping services from supporting Western companies who applied for a license to export exempted items into Iran. As a result, trade is constrained by the limited capacity to handle orders by existing arrangements.<br><br>Sanctions exacerbate international currency shortages in Iran. Penalizing oil trade terms prevent Iran to generate international currency from oil revenues. Western exporters respond to bottlenecks in trade services by curtailing credit lines to Iranian importers, so that current orders are mostly processed on cash-advance terms.<br><br>The resulting increase in operating capital requirements of Iranian importers of finished products and intermediate inputs cannot be passed on consumers by raising prices, due to price caps policies for drugs. As a consequence, many companies have failed, while others have survived by cutting product quality. Surviving companies are either more productive, or better connected to gain preferential access to capital from the government.	
114.	Nasheit (2003)[164]	UN sanctions (Aug 1990–May 2003).	Iraq	Neonatal, infant, and maternal mortality rates.	Average monthly number of laboratory examination and	Commentary based on published sources.	**No original research findings.**	Trends ascribed to sanctions.	Missing references for quoted data. Two other sources are mentioned, without

	1st Author / year / Journal	Type of econ sanctions	Country focus	Health-related outcomes	Health system outcomes	Methods	Main Findings	Evidence (or suggestion) of modification/mediation	Comments
				Prevalence of low birthweight.	major surgical operations.		In the 1990s, neonatal, infant, maternal mortality and the prevalence of low birthweight all increased, while the average monthly number of laboratory examinations and major surgical operations declined.		its data being discussed: the surveys of Ascherio et al (1992) and the UNICEF ICMMS survey (the reliability of the latter has been questioned).
115.	Ogbonna (2017)[165]	US sanctions (Dec 2001–ongoing) and EU restrictive measures (Feb 2002–ongoing)	Zimbabwe		Development aid, public expenditure for healthcare and education	Long commentary based on published sources.	**No original research findings**.<br><br>The article argues that sanctions led to decreased health and educational expenditure by obstructing multilateral and bilateral aid, and by worsening macroeconomic performance. Rejections of loan applications by the IMF and the African Development Bank, and discontinuation of aid programs by selected EU countries, are mentioned.	Decrease in export earnings and international reserves contributing to worse macroeconomic performance and associated fiscal consolidation through social spending cuts. Reduced access to development finance and aid.	Virtually no evidence is provided to support the empirical claims about the impact of sanctions on macroeconomic activity. In particular, the hypothesis that any macroeconomic impact of sanctions “forced” the government to cut social expenditure is not assessed against the alternative hypothesis that the government did in fact enjoy sufficient room to pursue alternative fiscal consolidation policies not involving those cuts, but chose not to.
116.	Parnham-Cope (1997)[166]	US embargo (Jul 1963–ongoing), esp. Helms-Burton Act (Mar 1996–ongoing)	Cuba	Self-inflicted injuries		Editorial quoting published sources	**No original research findings**.<br><br>The article comments on the epidemic of self-inflicted injuries among Cuba detainees in Guantanamo Bay (Andrews et al, 1997), linking the strengthening of US sanctions brought about by the Helms-Burton Act to worsened economic conditions in Cuba	Worsening economic conditions leading to outmigration and concomitant US immigration policy change towards Cuba	Concurs with Eisenberg (1997) in conjecturing a contribution of US sanctions to the episode.
117.	Peyravi and Ahmadi Marzaleh (2019)[167]	US sanctions (Nov 1979–ongoing), esp. measures	Iran			Correspondence	**No original research findings.**		

	1st Author / year / Journal	Type of econ sanctions	Country focus	Health-related outcomes	Health system outcomes	Methods	Main Findings	Evidence (or suggestion) of modification/mediation	Comments
		reintroduced after withdrawal from the Joint Comprehensive Plan of Action (Aug 2018–ongoing).					The letter identifies sanctions as a factor limiting the transfer of financial resources during the flood that occurred in March 2019, affecting about 2 million people. Constraints mentioned include purchases by the Red Crescent Society, UN cash transfers to its field personnel, and in-kind contributions of dual-use items such as rescue vehicles.		
118.	Popal (2000)[168]	UN sanctions (Aug 1990–May 2003).	Iraq	Average daily nutritional intake; infant and under-5 mortality rates.		Descriptive statistics based on administrative and survey data.	Relative to average intake before sanctions (1988/1990), the daily ration has 65% less calories (from 3120 to 1093 kcal), 67.4% less proteins (from 82.5 to 26.9 g.), 10.8% less fats (from 75.3 to 22 g.), 83.1% less calcium (from 467 to 79 g.), and 68.5% less iron (from 26 to 8.2 g.).<br><br>A secular increase of infant and under-5 mortality under sanctions is reported.		No pre-sanction baseline for point estimates quoted. Some data is presented only in graphical forms and cannot be extracted. Data from the Ministry of Health is facility-based, subject to changes in facility use patterns and thus unlikely to be nationally representative.<br><br>No source quoted for baseline nutrients intake and no time reference for nutrients intake from daily ration under sanctions.<br><br>The quoted infant and under-5 mortality figures come from the UNICEF ICMMS survey, the reliability of which has been questioned.
119.	Rezaee-Zavareh et al (2016)[169]	(unspecified)	Iran			Short communication	**No original research findings**.		According to the figures given in the article, Iran ranks

	1st Author / year / Journal	Type of econ sanctions	Country focus	Health-related outcomes	Health system outcomes	Methods	Main Findings	Evidence (or suggestion) of modification/mediation	Comments
							Among Middle Eastern countries over the 1996-2014 period, Iran ranks third for number of research papers and total citations in medicine, and fifth for H-index (data from a SCImago search).<br><br>In light of these statistics, Iran's performance in terms of international collaborations in medical research is deemed poor, and ascribed to a multiplicity of factors – including sanctions. It is concluded that the lifting of sanctions could improve the situation.		fourth in research collaborations in the sample, a result that is hardly different, and is in fact fully in line, with performance in terms of research output and impact.<br><br>It is therefore unclear in what way the figures support the claim about the potential effects of sanctions.
120.	Román (1995a)[170]	US embargo (Jul 1963–ongoing), esp. Cuban Democracy Act (Oct 1992–ongoing)	Cuba	Cases of neuropathy		Commentary based on published sources.	**No original research findings**.<br><br>The figures quoted point to an epidemic of neuropathy in Cuba in 1992-1993, with a total of 50.862 certified cases in January 1994. It is reported that the study was able to rule out exposure to neurotoxic and infectious agents as potential confounders, and that the most likely cause was identified in a deficiency of Vitamins B, mainly thiamine. Vitamin B supplementation was found to be highly effective and, once scaled up to population, brought the epidemic to an end.	Dietary changes, mainly consisting in increased cane sugar consumption, are thought to be the likely mediator between, on the one hand, the economic crisis and embargo and, on the other hand, vitamin B deficiency leading to epidemic neuropathy.<br><br>Suggested channels for the effect of sanctions are: higher import prices for feed and grains, laboratory equipment and reagents, materials for vitamin laboratory synthesis; severing of medical research collaborations and access to US medical information.	The author writes: "Although the US economic embargo may not have been the primary cause of the epidemic in Cuba, it has contributed to its development, complicated its investigation and treatment, and continues to hamper its prevention".
121.	Román (1995b)[171]	US embargo (Jul 1963–ongoing), esp. Cuban Democracy Act (Oct 1992–ongoing)	Cuba	Cases of neuropathy		Commentary based on published sources.	**No original research findings.**<br><br>The article summarizes evidence from published studies on the epidemiology,	Both analyses and successful treatment point to nutritional causes. These are in turn associated by the author to the double shock of Cuba's "special period" crisis. The role of the US embargo is	

	1st Author / year / Journal	Type of econ sanctions	Country focus	Health-related outcomes	Health system outcomes	Methods	Main Findings	Evidence (or suggestion) of modification/mediation	Comments
							symptomatology and likely etiology of the epidemic.<br><br>From the discovery of early cases in late 1991 to the eventual epidemic outbreak and remission after B-group vitamins supplementation in 1993 and 1994, total cases summed up to more than 50000. Patients displayed hearing and vision loss, “burning feet”, fatigue, weight loss, difficulties in locomotion, sleeping and concentration. Similar symptoms were observed among prisoner of war populations in the past.	evaluated as in Román (1995a): a contribution adding to existing adverse conditions.	
122.	Román (1998)[172]	US embargo (Jul 1963–ongoing), esp. Cuban Democracy Act (Oct 1992–ongoing)	Cuba	Cases of Neuropathy		Commentary based on published sources.	**No original research findings**.<br><br>The article reports findings from published research about an epidemic of peripheral neuropathy in Cuba during 1992 and 1993, linking it with the economic crisis of the period and implicating the tightening of the US embargo as a contributing factor.		The source closely tracks Román (1995a, 1995b).
123.	Sahraian et al (2021)[173]	US sanctions (Nov 1979–ongoing), esp. measures reintroduced after withdrawal from the Joint Comprehensive Plan of Action (Aug 2018–ongoing).	Iran		Self-reports on concerns on availability of treatment, treatment costs, and clinical status.	Unadjusted odd ratios (logistic regression) on cross-sectional survey from 2 facilities (Sample: 1039 Multiple Sclerosis patients)	Among the 998 respondents for which an income figure is given, more than 73% had a monthly income below 250 USD, contrasted with 55 USD of monthly treatment.<br><br>The following percentages of respondents reported: concerns for past and future treatment availability (85.8, 93.6), the former significantly larger among women (OR 1.55, 95%CI: 1.04-2.33); concerns for past and future		The absence of a pre-sanction baseline prevents obtaining an impact estimate.<br><br>No information on sampling criteria.

	1st Author / year / Journal	Type of econ sanctions	Country focus	Health-related outcomes	Health system outcomes	Methods	Main Findings	Evidence (or suggestion) of modification/mediation	Comments
							supply and purchase of foreign medicine (86, 89.2), for the effectiveness of domestic vs. foreign medicines (82%), for future replacement of international with domestic medicines (72.8), and more expensive with cheaper medicine (70.3) – the latter significantly higher among those with monthly income below 250 USD (OR 2.04, 95%CI: 1.50-2.77); unwillingness to continue treatment due to economic or psychological burden (41.4%, 42.9%), both significantly lower among women (OR 0.54, 95%CI: 0.40-0.73; OR 0.73, 95%CI: 0.53-0.93) and the former significantly higher among those with monthly income below 250 USD (OR 2.02, 95%CI: 1.49-2.73); cost increases in laboratory services (81.3%), hospitalization (64%), facility physiotherapy (51.89%), home rehabilitation (38.52%), transport to facility (67.63%), psychological/psychiatric counselling (52.22), living costs (93.53%), and mobility aid (38.65) – the latter significantly less among women (OR 0.70, 95%CI: 0.51-0.96); stopping or diminishing own social (60.11%) or physical activities (66.82%); loss of job insurance (47.95%), significantly less among women (OR 0.45,		

	1st Author / year / Journal	Type of econ sanctions	Country focus	Health-related outcomes	Health system outcomes	Methods	Main Findings	Evidence (or suggestion) of modification/mediation	Comments
							95%CI: 0.39-0.73); reduced nutrition quality (68.82%), significantly less among women (OR 0.69, 95%CI: 0.49-0.98) and among those with monthly income below 250 USD (OR 2.68, 95%CI: 1.99-3.60).		
124.	Sansom (2004)[174]	UN sanctions (Aug 1990–May 2003).	Iraq			Correspondence	**No original research findings**.<br><br>Interviewed Iraqi health professionals are quoted mentioning shortcomings of healthcare, including the existence of only 2 cobalt machines for radiotherapy and no linear accelerator in the country, leading to 2-3 months-long queues; a government policy of potentially arbitrary hoarding and testing of imported pharmaceuticals under sanctions; an increase in mortality from treatable cancers as a result of shortages in chemotherapy among patients who could not afford medical tourism in neighboring countries; a decrease in the age of diagnosis for certain types of cancer.<br><br>The report attributes the state of healthcare to sanctions, government policy under sanctions, but also mentions a weak nursing culture reflected in insufficient resources and training. Improvements in drug availability after the 2003 US invasion are attributed to initiatives by international NGOs.		

	1st Author / year / Journal	Type of econ sanctions	Country focus	Health-related outcomes	Health system outcomes	Methods	Main Findings	Evidence (or suggestion) of modification/mediation	Comments
125.	Sato et al (1991)[175]	UN sanctions (Aug 1990–May 2003).	Iraq	Prevalence of underweight, stunting, wasting.		Descriptive statistics from cross-sectional survey.	11 months after the imposition of sanctions and 5 months after the end of armed conflict, prevalence of moderate-to-severe ($Z \leq -2$) and severe ($Z \leq -3$) malnutrition was 20.7% and 7.8% for stunting, 22.4% and 6.8% for underweight, 6.9% and 1.5% for wasting.		Absence of baseline data prevents obtaining an impact estimate. Unclear treatment of outliers; no adjustment for mortality selection.
126.	Sen (2014)[176]	(unspecified)	Syria			Correspondence	**No original research findings**.<br><br>The letter argues that credible reports of child deaths due to malnutrition in conflict-affected areas, together with a sceptical literature on the effectiveness of sanctions, call into question the current foreign policy stance of Western countries.		
127.	Sen et al (2013)[177]	(unspecified)	Syria			Descriptive statistics based on government sources, UN agencies, and online sources.	Similar content to Al Faisal et al (2012a). See entry.		
128.	Setayesh and Mackey (2016)[178]	Sanctions by the US, UN and EU before the Joint Comprehensive Plan of Action (unspecified).	Iran		Reported shortages of pharmaceutical	Descriptive statistics from published sources.	Reports of shortages were found for 73 pharmaceuticals: 32 were included in the WHO essential list in 2015; 65 were primarily indicated for non-communicable diseases, the main cause of death in the country; 3 were not covered by exemptions to US sanctions.	Shortages attributed to imperfect exemption systems, including restrictions on shipping and trade insurance.	No clear time period attached to the figures. No comparison of reported shortages across time. No comparison of characteristics with pharmaceuticals not in shortage.
129.	Shahabi (2015)[179]	(unspecified)	Iran			Short communication	**No original research findings**.<br><br>The article is a letter expressing concerns about the adverse impact of sanctions on Iran's National Cancer Control Program. It		

	1st Author / year / Journal	Type of econ sanctions	Country focus	Health-related outcomes	Health system outcomes	Methods	Main Findings	Evidence (or suggestion) of modification/mediation	Comments
							points to "a serious shortage of cancer drugs in the nation" and argues that "there can be no improvement in the status of the NCCP" until sanctions are lifted.		
130.	Shahabi et al (2015)[180]	US and EU sanctions against Central Bank of Iran (unspecified).	Iran			Commentary	**No original research findings**.<br><br>Sanctions, especially those targeting banking and oil exports, imposed by the US, EU and other countries, led to accelerating inflation and subsequent macroeconomic contraction. Large currency devaluation worsened terms of trade in pharmaceutical imports and led to higher pharmaceutical prices, even as CPI declined during recession.<br><br>Given the large share of health expenditure met out-of-pocket, rising prices of pharmaceuticals, especially for chronic diseases, directly affect the ability of households to use medications. Sanctions against the oil trade might also have increased reliance on low-quality fuel for cooking and vehicles, leading to greater exposure to carcinogenic pollutants.	Declines in purchasing power for pharmaceuticals due to macroeconomic contraction and supply constraints on the pharmaceutical market due to currency devaluation.	
131.	Shahabi et al (2020)[181]	US sanctions (Nov 1979–ongoing), esp. measures reintroduced after withdrawal from the Joint Comprehensive Plan of Action	Iran		Expert opinion on the status of physical rehabilitation services	Thematic analysis on records of semi-structured face-to-face or telephone interviews with health policy-makers, researchers and registered	Four themes and 12 sub-themes were identified.<br><br>Socio-economic challenges, including: shrinking government revenues and expenditures; higher inflation, pushing up costs and leading insurance companies to remove		

	1st Author / year / Journal	Type of econ sanctions	Country focus	Health-related outcomes	Health system outcomes	Methods	Main Findings	Evidence (or suggestion) of modification/mediation	Comments
		(Aug 2018–ongoing).				practitioners with prolonged experience on physical rehabilitation (Sample: 37 individuals)	services; higher unemployment, reducing insurance premiums, increasing reimbursement delays, and increasing the risk of catastrophic expenditures; precarious employment situation of practitioners.<br><br>Educational challenges, including: barriers to international collaborations, events and publications; shortages of funding for training materials.<br><br>Service delivery challenges, including: shortage or quality decline of raw materials and spare parts for prostheses and medical equipment; lack of maintenance of infrastructure; limited access to foreign financial assistance; declining third sector activity.<br><br>Mentioned mitigating responses included innovations by indigenous knowledge-based companies.		
132.	Sidel (1999)[182]					Commentary based on published sources and UN documents.	**No original research findings**.<br><br>The author argues that, to the extent that fundamental human rights constitute obligations towards the international community, measures to enforce them against violations are only legitimate if, in addition to UN authorization, they do not themselves violate those rights. This principle		

	1st Author / year / Journal	Type of econ sanctions	Country focus	Health-related outcomes	Health system outcomes	Methods	Main Findings	Evidence (or suggestion) of modification/mediation	Comments
							requires that economic sanctions be designed to safeguard trade in essential goods, target political elites, and operate continuous monitoring of living standards in target countries.		
133.	Smith and Zaidi (1993)[183]	UN sanctions (Aug 1990–May 2003).	Iraq.	Prevalence of malnutrition (underweight, stunting, wasting).		Descriptive statistics from cross-sectional survey	Prevalence of moderate and severe malnutrition ($Z < -2$) at 21.8% for stunting, 11.9% for underweight, 3.4% for wasting. Prevalence of severe malnutrition ($Z < -3$) at 7.3% for stunting, 2.3% for underweight, 0.4% for wasting.<br><br>Inverse U-shaped age distribution of moderate and severe malnutrition: stunting peaks among children 18-23 months (38.5%), underweight and wasting among children 12-17 months (18.4% and 6.1%).<br><br>Malnutrition inversely correlated with maternal education, highest in Northern region. No significant differences across gender and urban/rural lines.<br><br>Reported presence of diarrhoea associated with stunting (RR: 1.8, 95% CI: 1.5,2.2), underweight (RR: 2.1, 95% CI: 1.6,2.7) and wasting (RR: 1.5, 95% CI: 0.9,2.4).	The authors note that prevalence of stunting peaks among children who were 6-11 months when sanctions were imposed, and suggest the peak might reflect acute malnutrition during weaning for the cohort, due to sanctions.	Data from subsample of Ascherio et al (1992).<br><br>Absence of baseline data prevents obtaining an impact estimate.<br><br>Uncertain adjustment of sampling frame for population displacement. No clustering of standard errors. No adjustment for mortality selection on both children and mothers.
134.	Spagat (2010)[20]	UN sanctions (Aug 1990–May 2003).	Iraq	Under-5 mortality		Commentary based on published sources and secondary survey data.	**No original research findings**.<br><br>Partial retraction after on-site verification, and stark contrast with surveys fielded		

	1st Author / year / Journal	Type of econ sanctions	Country focus	Health-related outcomes	Health system outcomes	Methods	Main Findings	Evidence (or suggestion) of modification/mediation	Comments
							after the fall of the Iraqi regime are suggestive of survey fraud for the FAO/NRI and ICMMS surveys respectively. Often quoted claims of half-million excess child deaths due to sanctions, based on those surveys, are thus unsupported.		
135.	Sponeck (2002)[184]	UN sanctions (Aug 1990–May 2003).	Iraq			Commentary based on published sources.	**No original findings**.<br><br>The author traces the failure to prevent adverse health outcomes of sanctions to lack of clarity on UN legal accountability, vagueness in sanctions resolutions, and the absence of a sanctions monitoring system. Measures to remedy those gaps are proposed.		
136.	Stix (1995)[185]	US embargo (Jul 1963–ongoing), esp. Cuban Democracy Act (Oct 1992–ongoing)	Cuba			Editorial	**No original research findings**.<br><br>The article discusses the outbreak of neuropathy in Cuba during 1992-1993 and the following campaign to advocate the lifting of sanctions on food and medicines by various health workers and organizations.		
137.	Takian et al (2020)[186]	US and EU sanctions (unspecified)	Iran			Correspondence	**No original findings**.<br><br>The letter argues that, in the current COVID-19 epidemic, "the detrimental effects of sanctions have reduced access to life-saving medicines and equipment"		
138.	The Cuba Neuropathy Field Investigation Team (1995)[187]	US embargo (Jul 1963–ongoing), esp. Cuban Democracy Act (Oct 1992–ongoing)	Cuba	Cases of neuropathy (optic form)		Adjusted odds ratios (logistic regression) on case-control pairs matched by sex, age and municipality,	Univariate risk factors include any tobacco use (6.6; 95%CI: 3.2-13.9), daily consumption of 1 to 3 (8.7; 95%CI: 2.5-30.7) and above 3 (22.8; 95%CI: 4-131) cigars	Damages to the optic nerve due to impaired cyanide detoxification, caused by B-complex vitamin deficiency. This deficiency is in turn deemed associated to adverse economic	Sampling limited to severe cases.<br><br>The study strongly suggests a nutritional origin of the epidemic, consistent

	1st Author / year / Journal	Type of econ sanctions	Country focus	Health-related outcomes	Health system outcomes	Methods	Main Findings	Evidence (or suggestion) of modification/mediation	Comments
						randomly selected in 5 municipalities of the Pinar del Rio province.	(relative to no consumption); share of energy intake from Cassava (3; 95%CI: 1.3-6.6) (highest to lowest quartile). Protective factors include higher (highest-to-lowest quartile) share of energy intake from animal proteins (0.3; 95%CI: 0.1-0.6), rearing poultry (0.4; 95%CI: 0.2-0.7); having relatives abroad (0.4; 95%CI: 0.2-0.6), animal fat (0.2; 95%CI: 0.1-0.5), methionine (0.3; 95%CI: 0.1-0.6), B12 Vitamin (0.2; 95%CI: 0.1-0.4), riboflavin (0.3; 95%CI: 0.2-0.7); higher serum density of Lycopene (0.05; 95%CI: 0.02-0.2), β-carotene (0.2; 95%CI: 0.1-0.4), Selenium (0.3; 95%CI: 0.1-0.6).<br><br>In multivariable models (not shown), cigar smoking, cassava consumption, serum Lycopene, total energy intake, methionine share in total energy, and poultry rearing reportedly remained significant, although other factors could be substituted with little loss of explanatory power.	conditions, including "the loss of Cuba's major trading partners and a severe storm".	with a contributory role of barriers to food imports, including the collapse of the Soviet Union, the pre-existing US embargo and its subsequent tightening. However, its design does not allow separate quantification of these factors.
139.	The Lancet (1995)[188]	UN sanctions (Aug 1990–May 2003).	Iraq			Editorial	**No original research findings**.<br><br>Findings in Zaidi and Fawzi (1995) are consistent with UN sources and field reports, pointing to shortages of medicines, rising		Discussion partly based on FAO/NRI survey findings (Zaidi and Fawzi, 1995), later partly retracted (Zaidi, 1997).

	1st Author / year / Journal	Type of econ sanctions	Country focus	Health-related outcomes	Health system outcomes	Methods	Main Findings	Evidence (or suggestion) of modification/mediation	Comments
							morbidity and mortality, and decline in vaccine coverage. Attribution to war versus sanctions is difficult, and Iraqi civil servants may be deterred from revealing accurate information.		
140.	The Lancet (1996)[189]	US embargo (Jul 1963–ongoing), esp. Cuban Democracy Act (Oct 1992–ongoing)	Cuba			Editorial	**No original research findings.**<br><br>Brief note linked to Kirkpatrick (1996)		
141.	Velayati et al (2015)[190]	US sanctions (unspecified)	Iran			Correspondence	**No original research findings**.<br><br>The letter announces the establishment of a non-profit organization to seek opportunities for procurements of chemotherapy drugs that are in short supply in Iran.		
142.	Wakai (2000)[191]	UN sanctions (Aug 1990–May 2003).	Iraq			Correspondence	**No original research findings**.<br><br>The letter mentions findings from Ali and Shah (2000), and advocates the lifting or relaxation of sanctions.		Findings quoted are based on UNICEF ICMMS survey, the reliability of which has been questioned.
143.	Wareham (2000)[192]	UN sanctions (Aug 1990–May 2003).	Iraq			Editorial	**No original research findings**.<br><br>Personal recollection of field visit in Iraq. Salient observations include: shortage of drugs and surgical supplies in visited hospitals; an increase in cases of major infections reported by doctors at Saddam Pediatric Hospital in Baghdad; blocked and broken sewerage pipes in visited hospitals; lack of access to foreign handbooks reported by doctors at Baghdad's University		Discussion partly based on UNICEF's ICMMS survey, the reliability of which has been questioned.

	1st Author / year / Journal	Type of econ sanctions	Country focus	Health-related outcomes	Health system outcomes	Methods	Main Findings	Evidence (or suggestion) of modification/mediation	Comments
							College of Medicine; an increase in the incidence of child leukaemia and congenital abnormalities, especially in Southern Iraq, reported by various doctors.		
144.	Weeramanthri et al (2001)[193]	US embargo (Jul 1963–ongoing), esp. Cuban Democracy Act (Oct 1992–ongoing)	Cuba			Short communication	**No original research findings**.<br><br>The article is a letter that relies on secondary sources to argue that the US embargo adversely impacts population health in Cuba, although the resilience allowed by the local healthcare system is also mentioned.		
145.	WHO (2002)[194]		Various countries	Child mortality, prevalence of chronic child malnutrition.	Availability of healthcare inputs, restriction of travel for healthcare workers and patients.	Commentary based on published sources, national statistics and reports by UN agencies.	**No original research findings.**<br><br>In Iraq, the Gulf war and the parallel UN sanctions led to a deterioration in many health indicators, including child mortality and the prevalence of child malnutrition as measured by multiple anthropometric indicators.<br><br>In Afghanistan, the freezing of government assets and the ban on flights of the national company implemented by UN sanctions in 1999 had repercussions on the wider society. Humanitarian efforts were restricted due to rioting after the imposition of sanctions. The Indira Ghandi Pediatric Hospital in Kabul had its air-based drug supply channel cut due to the flight ban. Sanctions contributed to currency devaluation. Isolating the contribution of sanctions is made difficult	It is suggested that, in Iraq, the impact of sanctions worked both through supply constraints to essential health inputs such as drugs and vaccines, and through general-purposes goods and services with important applications in health systems such as fuel, energy, mechanical spare parts and chemicals of various type.	

	1st Author / year / Journal	Type of econ sanctions	Country focus	Health-related outcomes	Health system outcomes	Methods	Main Findings	Evidence (or suggestion) of modification/mediation	Comments
							by the coexistence of long-standing armed conflict and instability, a picture further compounded by a devastating drought in 2000.<br><br>UN sanctions imposed against Libya in 1992 froze government assets and banned travel. A UN mission later reported Shortages of essential drugs and vaccines, spare parts, denial of transportation abroad for the critically ill, delays in sourcing blood products, serums, laboratory reagents, and termination of contracts or entry denials for health workers. No quantification of the impact has been made.		
146.	Yamada et al (2006)[195]	UN sanctions (Aug 1990–May 2003).	Iraq			Commentary based on published academic and media sources	**No original research findings**.<br><br>Discussing the role of the media in communicating the impact of war and sanctions on civilians, the findings of Harvard Study Team (1991), Ascherio et al (1992), and Ali and Shah (2000) are quoted.		Discussion partly based on UNICEF's ICMMS survey, the reliability of which was later questioned.
147.	Yazdi-Feyzabadi et al (2020)[196]	US sanctions (Nov 1979–ongoing), esp. measures reintroduced after withdrawal from the Joint Comprehensive Plan of Action (Aug 2018–ongoing).	Iran			Commentary based on published sources.	**No original research findings**.<br><br>Channels of the health impact of sanctions are mentioned, including macroeconomic effects on currency exchange, inflation and unemployment, lowering the availability of, and public and private expenditure on, healthcare services, pharmaceuticals, and medical imports.		

	1st Author / year / Journal	Type of econ sanctions	Country focus	Health-related outcomes	Health system outcomes	Methods	Main Findings	Evidence (or suggestion) of modification/mediation	Comments
							These effects are related to the Social Determinants of Health framework, and deemed to impact all its three main components: socio-economic-political context, structural factors and intermediary factors. A health diplomacy approach is proposed as a mechanism of dispute resolution alternative to sanctions.		
148.	Yoon et al (2019)[197]	Sanctions by UN and US (unspecified)	Korea DPR		Characteristics of medical research articles	Chi-square and Mann-Whitney U test on time series of bibliographic data (Sample: 775 articles from 28 issues of one medical journal, selected at 5-years interval from 1985 to 2005).	The proportion of articles classified as 'conventional therapies: medication' decreased from 25.3% in the two years 2000 and 2005 to 15.6% (p<.05); articles classified as 'diagnosis' decreased from 41.3% to 13.9% (p<.001); articles classified as 'nonconventional therapies: alternative medicine' increased from 6.7% to 57.2% (p<.001). No significant change for categories 'conventional therapies: surgery', 'nonconventional therapies: traditional medicine'.	Shortages of pharmaceuticals are proposed as a potential cause of changes in research on medication and alternative medicine, while the decrease in focus on traditional medicine is taken to reflect long-run policies to integrate traditional medicine in the healthcare system.	Both descriptive statistics and significance tests are reported only for some of the categories, and no justification is provided for this choice.<br><br>An additional comparison is reported for international aid volume, but the study contain no clear discussion of aid, nor hypotheses about aid. Hence, the result is not considered.
149.	Younis and Aswad (2018)[198]	UN sanctions (Aug 1990–May 2003).	Iraq	Prevalence of PTSD, anxiety and depressive disorders.	Attendance in outpatient clinics, psychiatrist density	Descriptive statistics from published literature in the 1990-2003 period.	Retrieved findings include: shortage of psychiatric drugs, closure of a facility, and cases of deaths due to neglect and lack of staff in psychiatric hospitals; an increase in attendees of outpatient clinics from 197 000 to 220 000 to 507 000 in 1990, 1994 and 1998 respectively; a prevalence of PTSD of 10% in the general population (2005 and 2006 sources), between 24% and 57% in a 2003 sample of Baghdad		Incomplete reporting of key characteristics for many studies, and ambiguous relationship with sanctions for all findings reported.

	1st Author / year / Journal	Type of econ sanctions	Country focus	Health-related outcomes	Health system outcomes	Methods	Main Findings	Evidence (or suggestion) of modification/mediation	Comments
							schoolchildren exposed to bombing in 1991; 87% and 20% in two samples of Kurdish children exposed to military operations; in a facility in Baghdad, a prevalence of anxiety and depressive disorders of 18.9% and 16% (10 101 patients) in 1993, rising to 33.4% and 22.2% (1315 patients) in 2003; an estimated decrease in psychiatrists per 100 000 population from 0.5 "before sanctions" to 0.1 in 1998.		
150.	Yousefi et al (2019)[199]	US, UN, EU sanctions before the Joint Comprehensive Plan of Action, especially National Defense Authorization Act (Jan 2013) and Iran Threat Reduction and Syria Human Rights Act (Aug 2012).	Iran		Inflation in pharmaceuticals and health commodities. Reported pharmaceuticals in shortage	Focus group on policies to improve access to pharmaceuticals (sample: 5 operators at Iran Federal Drug Administration) and descriptive statistics from administrative data.	In 2014 and 2015, average annual inflation in pharmaceutical commodities was respectively 19% and 13% lower than in overall health commodities. Price reductions in pharmaceuticals in the two years is reported to have led to 350 mln. USD. Out-of-pocket expenditure in pharmaceuticals targeted by insurance subsidies decreased 45% during 2013-2015. Reported number of pharmaceuticals in shortage declined from 270 (2012) to 170 (2013) and 30 (2014, 2015, 2016).	Declines in prices and shortages attributed to mitigation policies, including simplified custom clearance for medicines; priority to IFDA in central bank foreign currency allocations; health insurance subsidies for selected conditions and patients at risk of catastrophic expenditure; tariffs and quotas on imported items with domestic counterpart; reformed inventory management; price controls based on parallel imports and tendering.	No baseline give for the reported price reduction. No policy implementation time.
151.	Zadeh-Cummings and Harris (2020)[200]	Sanctions by the UN (Oct 2006–ongoing) and the US ()	Korea DPR		Expert opinion on operational outcomes in humanitarian action	Report based on UN documents and reports by NGOs, local and international media sources, and semi-structured interviews with operators from 8 relief agencies	Four themes are identified from the interviews.<br><br>Problems with exemption systems. For US sanctions, these included large legal costs, and long/unreliable processing times in licensing, and ambiguity of rules such as prohibition of collaboration with government personnel. For		

	1st Author / year / Journal	Type of econ sanctions	Country focus	Health-related outcomes	Health system outcomes	Methods	Main Findings	Evidence (or suggestion) of modification/mediation	Comments
						(Sample: 8 individuals)	UN sanctions, uncertainty over the extent of disclosure of non-sanctioned items in license applications.<br><br>Problems with third parties under US secondary sanctions, including blocked international bank transfers, refusal of banking services due to overcompliance, higher transaction costs charged by suppliers.<br><br>Problems with sanctioning governments, including sequestering of exempted items at customs, evidence of political influence on licensing.<br><br>Distorted incentives and deterrence on humanitarian action, as some agencies adapt programs to minimize the mentioned barriers, while others are discouraged into expanding operations in the country.		
152.	Zaidi (1994)[201]	UN sanctions (Aug 1990–May 2003).	Iraq (Baghdad, Saddam City district)	Prevalence of under-5 malnutrition (underweight, stunting, wasting).		Descriptive statistics from panel survey.	Between baseline (August 1991) and follow-up (November 1993), moderate-to-severe malnutrition increased 16% to 30% (stunting), 9% to 35% (underweight), and 2.2% to 16% (wasting).	Effect attributed to sanctions, independent of previous armed conflict.	Follow-up of a subset of 8 clusters of Ascherio et al (1992).<br><br>Uncertain adjustment of sampling frame for population displacement. No clustering of standard errors. Unexplained limitation of comparison to 6 of the 8 clusters sampled.<br><br>No pre-sanction baseline, or measure of cumulative exposure. Attribution

	1st Author / year / Journal	Type of econ sanctions	Country focus	Health-related outcomes	Health system outcomes	Methods	Main Findings	Evidence (or suggestion) of modification/mediation	Comments
									of impact to sanctions assumes cumulative exposure and no lagged confounding or effect modification due to armed conflict.
153.	Zakavi (2019)[202]	US sanctions (Nov 1979–ongoing), esp. measures reintroduced after withdrawal from the Joint Comprehensive Plan of Action (Aug 2018–ongoing).	Iran			Short communication	**No original research findings**.<br><br>The article argues that newly imposed US sanctions are likely to affect adversely the provision of nuclear medicine diagnostics and treatment, through an increase in the risks and costs associated with the import of intermediate inputs in the production of radiopharmaceuticals.		
154.	Zartab et al (2020)[203]	(unspecified)	Iran		Health expenditure, market shares of pharmaceutical companies, pharmaceutical sales and import volumes.	Descriptive statistics from government sources	Health expenditure as a share of GDP remained constant over the 2001-2016 period, despite large GDP fluctuations. The out-of-pocket component increased after 2012, mirroring a falling government share.<br><br>Among the top 6 holding groups in the pharmaceutical sector, 3 were private and 3 state-owned, with market shares going from 17.5% to 5.3%. Among the top 10 domestic pharmaceutical producers in 2016, 4 were private and 6 state-owned, and none had a marked share above 5%, adding up to 34.98%. Among the top 10 importers, all private, market share ranges from 18.83 to 2.31, adding up to 70.76%.<br><br>Pharmaceutical sales grew from 2008 to 2012, declined in 2013, and recovered in		No definition of exposure period for the outcome presented.

	1st Author / year / Journal	Type of econ sanctions	Country focus	Health-related outcomes	Health system outcomes	Methods	Main Findings	Evidence (or suggestion) of modification/mediation	Comments
							2016, with recovery driven by consumption of domestic products.<br><br>The annual and compound growth of the market were 48.67% and 21.93% before and 20.49% and 9.77% after sanctions, respectively. The analogous rates for imports were 45.79% and 20.75% before and 9.54% and 4.66% after sanctions.		
155.	Zolotarev et al (2020)[204]				Annual medical publications; clusters in networks of authors, research institutions, and countries.	Descriptive statistics and network maps on bibliometric data (Sample: not shown)	Publications retrieved for Dimensions searches “Sanctions of Iranian Scientists” and “Economic Sanctions of Iranian Medicine and Health Science” show a positive time trend, and an anomalous peak in 2014. Mental maps of selected sub-samples of publications from the latter search show a high degree of fragmentation and isolation of scholars, little overlap in quoted sources, 5 country clusters of research collaborations, and 6 organization clusters.<br><br>Publications retrieved for a PubMed search of Iran-affiliated authors show a growing trend until 2011, followed by large decline and subsequent stagnation. Publications retrieved for a PubMed search “Economic Sanctions” show a first wave of sources in the 1990s and a second wave of sources from the mid-2000s onwards. A mental map of the last search included 54 terms organized in 5 clusters.		The 2014 peak in items retrieved in two searches is attributed to relaxed US sanctions, but no reference to a specific measure is given..

A6: Research reporting guideline checklists.

Table A6.1: PRISMA 2020 checklist. Source: Page et al.[2]

Section and Topic	Item #	Checklist item	Location where item is reported
**TITLE**			
Title	1	Identify the report as a systematic review.	p 1
**ABSTRACT**			
Abstract	2	See the PRISMA 2020 for Abstracts checklist.	Suppl p 111
**INTRODUCTION**			
Rationale	3	Describe the rationale for the review in the context of existing knowledge.	pp 1-2
Objectives	4	Provide an explicit statement of the objective(s) or question(s) the review addresses.	p 2
**METHODS**			
Eligibility criteria	5	Specify the inclusion and exclusion criteria for the review and how studies were grouped for the syntheses.	p 2
Information sources	6	Specify all databases, registers, websites, organisations, reference lists and other sources searched or consulted to identify studies. Specify the date when each source was last searched or consulted.	p 2, Suppl p 1
Search strategy	7	Present the full search strategies for all databases, registers and websites, including any filters and limits used.	Suppl p 1
Selection process	8	Specify the methods used to decide whether a study met the inclusion criteria of the review, including how many reviewers screened each record and each report retrieved, whether they worked independently, and if applicable, details of automation tools used in the process.	p 2
Data collection process	9	Specify the methods used to collect data from reports, including how many reviewers collected data from each report, whether they worked independently, any processes for obtaining or confirming data from study investigators, and if applicable, details of automation tools used in the process.	p 2, Suppl p 1
Data items	10a	List and define all outcomes for which data were sought. Specify whether all results that were compatible with each outcome domain in each study were sought (e.g. for all measures, time points, analyses), and if not, the methods used to decide which results to collect.	p 2, Suppl p 6
	10b	List and define all other variables for which data were sought (e.g. participant and intervention characteristics, funding sources). Describe any assumptions made about any missing or unclear information.	p 2, Suppl p 6
Study risk of bias assessment	11	Specify the methods used to assess risk of bias in the included studies, including details of the tool(s) used, how many reviewers assessed each study and whether they worked independently, and if applicable, details of automation tools used in the process.	pp 2, 4, Suppl pp 7-10
Effect measures	12	Specify for each outcome the effect measure(s) (e.g. risk ratio, mean difference) used in the synthesis or presentation of results.	Suppl p 8
Synthesis methods	13a	Describe the processes used to decide which studies were eligible for each synthesis (e.g. tabulating the study intervention characteristics and comparing against the planned groups for each synthesis (item #5)).	pp 2, 4, Suppl p 6
	13b	Describe any methods required to prepare the data for presentation or synthesis, such as handling of missing summary statistics, or data conversions.	Suppl p 8
	13c	Describe any methods used to tabulate or visually display results of individual studies and syntheses.	Suppl p 8

Section and Topic	Item #	Checklist item	Location where item is reported
	13d	Describe any methods used to synthesize results and provide a rationale for the choice(s). If meta-analysis was performed, describe the model(s), method(s) to identify the presence and extent of statistical heterogeneity, and software package(s) used.	pp 2, 4, Suppl pp 7-10
	13e	Describe any methods used to explore possible causes of heterogeneity among study results (e.g. subgroup analysis, meta-regression).	p 4
	13f	Describe any sensitivity analyses conducted to assess robustness of the synthesized results.	p 6, Suppl p 8.
Reporting bias assessment	14	Describe any methods used to assess risk of bias due to missing results in a synthesis (arising from reporting biases).	p 6, Suppl p 8.
Certainty assessment	15	Describe any methods used to assess certainty (or confidence) in the body of evidence for an outcome.	N/A
**RESULTS**			
Study selection	16a	Describe the results of the search and selection process, from the number of records identified in the search to the number of studies included in the review, ideally using a flow diagram.	Suppl p 5
	16b	Cite studies that might appear to meet the inclusion criteria, but which were excluded, and explain why they were excluded.	Suppl p 1
Study characteristics	17	Cite each included study and present its characteristics.	Suppl pp 25-107.
Risk of bias in studies	18	Present assessments of risk of bias for each included study.	Suppl pp 25-38.
Results of individual studies	19	For all outcomes, present, for each study: (a) summary statistics for each group (where appropriate) and (b) an effect estimate and its precision (e.g. confidence/credible interval), ideally using structured tables or plots.	Suppl pp 25-38.
Results of syntheses	20a	For each synthesis, briefly summarise the characteristics and risk of bias among contributing studies.	pp 4-6
	20b	Present results of all statistical syntheses conducted. If meta-analysis was done, present for each the summary estimate and its precision (e.g. confidence/credible interval) and measures of statistical heterogeneity. If comparing groups, describe the direction of the effect.	p 6, Suppl p 9
	20c	Present results of all investigations of possible causes of heterogeneity among study results.	pp 7-12
	20d	Present results of all sensitivity analyses conducted to assess the robustness of the synthesized results.	p 6, Suppl p 9
Reporting biases	21	Present assessments of risk of bias due to missing results (arising from reporting biases) for each synthesis assessed.	p 6, Suppl p 9
Certainty of evidence	22	Present assessments of certainty (or confidence) in the body of evidence for each outcome assessed.	N/A
**DISCUSSION**			
Discussion	23a	Provide a general interpretation of the results in the context of other evidence.	p 12-14
	23b	Discuss any limitations of the evidence included in the review.	p 4, 6, 12-13

Section and Topic	Item #	Checklist item	Location where item is reported
	23c	Discuss any limitations of the review processes used.	p 12-13
	23d	Discuss implications of the results for practice, policy, and future research.	p 13-14
**OTHER INFORMATION**			
Registration and protocol	24a	Provide registration information for the review, including register name and registration number, or state that the review was not registered.	13
	24b	Indicate where the review protocol can be accessed, or state that a protocol was not prepared.	13
	24c	Describe and explain any amendments to information provided at registration or in the protocol.	N/A
Support	25	Describe sources of financial or non-financial support for the review, and the role of the funders or sponsors in the review.	p 14
Competing interests	26	Declare any competing interests of review authors.	p 14
Availability of data, code and other materials	27	Report which of the following are publicly available and where they can be found: template data collection forms; data extracted from included studies; data used for all analyses; analytic code; any other materials used in the review.	p 14

Table A6.2: PRISMA 2020 for Abstracts checklist. Source: Page et al.[2]

Section and Topic	Item #	Checklist item	Reported (Yes/No)
**TITLE**			
Title	1	Identify the report as a systematic review.	YES
**BACKGROUND**			
Objectives	2	Provide an explicit statement of the main objective(s) or question(s) the review addresses.	YES
**METHODS**			
Eligibility criteria	3	Specify the inclusion and exclusion criteria for the review.	YES
Information sources	4	Specify the information sources (e.g. databases, registers) used to identify studies and the date when each was last searched.	YES
Risk of bias	5	Specify the methods used to assess risk of bias in the included studies.	YES
Synthesis of results	6	Specify the methods used to present and synthesise results.	YES
**RESULTS**			
Included studies	7	Give the total number of included studies and participants and summarise relevant characteristics of studies.	YES
Synthesis of results	8	Present results for main outcomes, preferably indicating the number of included studies and participants for each. If meta-analysis was done, report the summary estimate and confidence/credible interval. If comparing groups, indicate the direction of the effect (i.e. which group is favoured).	YES
**DISCUSSION**			
Limitations of evidence	9	Provide a brief summary of the limitations of the evidence included in the review (e.g. study risk of bias, inconsistency and imprecision).	YES
Interpretation	10	Provide a general interpretation of the results and important implications.	YES
**OTHER**			
Funding	11	Specify the primary source of funding for the review.	YES
Registration	12	Provide the register name and registration number.	NO

A7: Author reflexivity statement.

Table A7.1: authors' reflexivity statement: Source: Morton et al.[205]

1	**How does this study address local research and policy priorities?**	In this study, the author located in a LMIC-based institution (CH) has not taken part in core research activities. CH has worked extensively as an advisor in both high-income and LMIC settings. In line with the nature of his appointment as Chef de Cabinet in the Eastern Mediterranean Regional Office of the World Health Organization (EMRO-WHO), CH's main role in the project has been to commission the study and provide guidance and advice during its implementation. As many member states in the Eastern Mediterranean region have experienced – or are currently experiencing – economic sanctions, the study addresses an important need to develop an evidence base on how these countries can adjust their health sectors and policies under these conditions.
2	**How were local researchers involved in study design?**	See point 1. In addition to conceiving the study and mobilizing resources, CH has evaluated preliminary drafts and participated to the writing of the final draft of this article.
3	**How has funding been used to support the local research team?**	Due to the role of the LMIC-based author, no local research financing needs were identified.
4	**How are research staff who conducted data collection acknowledged?**	As the study is a review, the only data collection performed consisted in the extraction of data from included studies. This was performed by two authors, as acknowledged in the contributor statements.
5	**Do all members of the research partnership have access to study data?**	All authors have a copy of all data elaborations (tables, graphs). The author who curated the storing of extracted study attributes in appropriate formats (MPP) has the source files.
6	**How was data used to develop analytical skills within the partnership?**	In the context of this review, analytical skills related to the extraction, processing and use of the relevant data were deemed sufficient at the outset. Hence, no specific activity to develop them was performed. However, opportunities for a more general transfer of knowledge and skills from the more experienced researcher (MS) to the early career researcher (MPP) were seized at various stages of the project.
7	**How have research partners collaborated in interpreting study data?**	Key research steps required interpretive work, such as the elaboration of a logic graph to map the thematic narrative component of the review, the proposal of a plausible causal model, and the implementation of the thematic narrative. In line with the disclosed and above-mentioned repartition of contributions, this work was carried out by MS and MPP. Inputs from CH were incorporated at a later stage, in the context of a general evaluation of preliminary drafts. Most of the collaboration in this sense took place via periodic online meetings and mail exchanges.
8	**How were research partners supported to develop writing skills?**	As all authors are fluent in written academic English, no need to further develop writing skills was identified.
9	**How will research products be shared to address local needs?**	We aim to publish this review as open access. We are planning dissemination activities in cooperation with EMRO-WHO, and the possible release of further material such as policy reports on the topic. The communication office at the research institute at which MPP and MS are affiliated will also be mobilized for this purpose. The aim is to

		seize the opportunity presented by the current presence of sanctions as a subject in the press of many high-income countries to expand the associated narrative, which currently largely ignores the potential impacts of sanctions on population health – especially in low- and middle-income countries. In this way, we aim to align to EMRO policy.
10	**How is the leadership, contribution and ownership of this work by LMIC researchers recognised within the authorship?**	The inputs of CH, the LMIC-based author, in developing the manuscript have been recognized by including him as an author.
11	**How have early career researchers across the partnership been included within the authorship team?**	The early career researcher (MPP) in the project is located in a high-income country, and has been included as first author and correspondent. His crucial role at all stages of the project is recognized and detailed in the contribution statement.
12	**How has gender balance been addressed within the authorship?**	All three authors are male. Given the small size of the authorship team, it was felt that its composition does not raise gender balance concerns. We note that all institutions to which the authors are affiliated consistently pursue gender balance in all aspects of their functioning. See eg LISER's current Gender Equity plan.
13	**How has the project contributed to training of LMIC researchers?**	The LMIC-based author is a senior policy advisor, and no training need was identified.
14	**How has the project contributed to improvements in local infrastructure?**	No local infrastructure was used for this research, and no fruitful way to contribute to improvements in any such infrastructure in the context of the research could be found.
15	**What safeguarding procedures were used to protect local study participants and researchers?**	As no primary data collection was involved in the project, no safeguarding needs could be identified.

References

1 Guesenbauer M, Haddaway NR. Which academic search systems are suitable for systematic reviews or neta-analyses? Evaluating retrieval qualities of Google Scholar, PubMed, and 26 other resources. *Res Synth Methods* 2020;11:181–217.

2 Page MJ, McKenzie J, Bossuyt PM, et al. The PRISMA 2020 statement: an upgraded guideline for reporting systematic reviews. *BMJ* 2021;372:71.

3 Bärnighausen T, Tugwell P, Røttingen J, et al. Quasi-experimental study designs series – Paper 4: uses and value. *J Clin Epidemiol* 2017;89:21–9.

4 Geldsetzer P, Fawzi W. Quasi-experimental study designs series – Paper 2: complementary approaches to advancing global health knowledge. *J Clin Epidemiol* 2017;89:12–6.

5 Rockers PC, Røttingen J, Shemilt I, et al. Inclusion of quasi-experimental studies in systematic reviews of health systems research. *Health Policy* 2015;119:511–21.

6 Sterne JAC, Hernán MA, Reeves BC, et al. ROBINS-I: a tool for assessing risk of bias in non-randomised studies of interventions. *BMJ* 2016;355:i4919.

7 Sterne JAC, Hernán MA, Reeves BC, et al. ROBINS-I: a tool for assessing risk of bias in non-randomised studies of interventions. Appendix: the seven domains of bias addressed in the ROBINS-I assessment tool. *BMJ* 2016;335:i4919.

8 McKenzie J, Brennan SE. Synthesizing and presenting findings using other methods. In: Higgins JPT, Thomas J, Chandler J, Cumpston M, Li T, Page MJ, Welch VA eds. Cochrane handbook for systematic reviews of interventions. Chichester, John Wiley & Sons 2019:321–48.

9 Hilton Boon M, Thomson H. The effect direction plot revisited: application of the 2019 Cochrane handbook guidance on alternative synthesis methods. *Res Synth Methods* 2021;1:29–33.

10 Ogilvie D, Fayter D, Petticrew M, et al. The harvest plot: a method for synthesising evidence about the differential effects of interventions. *BMC Medical Res Methodol* 2008;8:8.

11 StataCorp. Stata Statistical Software. College Station, Texas, USA, 2021.

12 Nikolakopoulos S. Misuse of the sign test in narrative synthesis of evidence. *Res Synth Methods* 2020;11:714–19.

13 Daponte BO, Garfield R. The effect of economic sanctions on the mortality of Iraqi children prior to the 1991 Persian Gulf War. *Am J Public Health* 2000;90:546–52.

14 Ascherio A, Chase R, Coté T, et al. Effect of the Gulf War on infant and child mortality in Iraq. *N Engl J Med* 1992;327:931–36.

15 Harvard Study Team. The effect of the gulf crisis on the children of Iraq. *N Engl J Med* 1991;325:977–80.

16 Drèze J, Gazdar H. Hunger and poverty in Iraq, 1991. *World Development* 1992;20:921–45.

17 Zaidi S, Fawzi MCS. Health of Baghdad's children. *Lancet* 1995;346:1485.

18 Zaidi S. Child mortality in Iraq, *Lancet* 1997;350:1105.

19 Garfield R, Leu C. A multivariate method for estimating mortality rates among children under 5 years from health and social indicators in Iraq. *Int J Epidemiol* 2000;29:510–15.

20 Spagat M. Truth and death in Iraq under sanctions. *Significance (Oxford, England)* 2010;7:116–20.

21 Ali MM, Shah IH. Sanctions and chidhood mortality in Iraq. *Lancet* 2000;355:1851–57.

22 Mason JB, Brun T, Chen J, et al. The impact of the Oil-for-food programme on the Iraqi people: report of an independent working group established by the independent inquiry committee appointed to investigate the United Nations Oil-for-food programme, 2005. https://reliefweb.int/report/iraq/impact-oil-food-programme-iraqi-people (accessed Oct 2022).

23 Blacker J, Ali MM, Jones G. A response to criticism of our estimates of under-5 mortality in Iraq. *Popul Stud (Camb)* 2007;61:7–13.

24 Dyson T. Child mortality in Iraq since 1990. *Econmic and Political Weekly* 2006;41:4487–96.

25 Dyson T. New evidence on child mortality in Iraq. *Economic and Political Weekly* 2009;44:56–9.

26 Dyson T, Cetorelli V. Changing views on child mortality and economic sanctions in Iraq: a history of lies, damned lies and statistics, *BMJ Glob Health* 2017;2:e000311.

27 Garfield R. Studies on young child malnutrition in Iraq. *Nutr Rev* 2000;58:269–77.

28 Iraq Ministry of Planning and Development Cooperation. Iraq Living Conditions Survey 2004. Volume II: analytical report. Baghdad 2005. https://www.ecoi.net/en/document/2024352.html (accessed Oct 2022).

29 Allen CK, Fleuret J, Ahmed J. Data quality in Demographic and Health Surveys that used long and short questionnaires. Rockville MD: ICF 2020.

30 Daponte BO. Wartime estimates of Iraqi civilian casualties. *International Review of the Red Cross* 2007;89:943–57.

31 Garfield R. Morbidity and mortality among Iraqi children from 1990 to 1998: assessing the impact of economic sanctions. Notre Dame IN: Joan B. Kroc Institute for International Peace Studies and Fourth Freedom Forum, 1999. https://reliefweb.int/report/iraq/morbidity-and-mortality-among-iraqi-children-1990-through-1998-assessing-impact-gulf-war (accessed Oct 2022).

32 Garfield R. The public health impact of sanctions: contrasting responses of Iraq and Cuba. *Middle East Report* 2000;215:16–9.

33 Preston S, Guillot M, Heuveline P. Demography: Measuring and Modeling Population Processes. Oxford: Blackwell 2001.

34 United Nations Department of Economic and Social Development. Child mortality since the 1960s: a database for developing countries. New York, NY: United Nations 1992.

35 Al-Ani ZR, Al-Hiali SJ, Al-Farraji HH. Secular trend of infant mortality rate during wars and sanctions in western Iraq. *Saudi Med J* 2011;32:1267–73.

36 Ali HYM. Hepatitis B infection among Iraqi children: the impact of sanctions. *East Mediterr Health J* 2004;10:6–11.

37 Asadi-Pooya AA, Azizimalamiri R, Badv RS, et al. Impacts of the international economic sanctions on Iranian patients with epilepsy. *Epilepsy Behav* 2019;95:166–68.

38 Bundervoet T, Verwimp P. Civil war and economic sanctions: an analysis of anthropometric outcomes in Burundi. Brighton: University of Sussex 2005.

39 Garfield R. Economic sanctions, health, and welfare in the Federal Republic of Yugoslavia. Belgrade: OCHA and UNICEF, 2001. https://reliefweb.int/report/serbia/economic-sanctions-health-and-welfare-federal-republic-yugoslavia-1990-2000 (accessed Oct 2022).

40 Garfield R, Santana S. The impact of the economic crisis and the US embargo on health in Cuba. *Am J Public Health* 1997;87:15–20.

41 Ghiasi G, Rashidian A, Kebriaeezadeh A, et al. The impact of the sanctions made against Iran on availability to asthma medicines in Tehran. *Iran J Pharm Res* 2016;15:567–71.

42 Gutmann J, Neuenkirch M, Neumeier F. Sanctioned to death? The impact of economic sanctions on life expectancy and its gender gap. *Journal of Development Studies* 2021;57:139–62.

43 Karimi M, Haghpanah S. The effects of economic sanctions on disease specific clinical outcomes of patients with thalassemia and hemophilia in Iran. *Health Policy* 2015;119:239–43.

44 Kheirandish M, Varahrami V, Kebriaeezade A, et al. Impact of economic sanctions on access to noncommunicable diseases medicines in the Islamic Republic of Iran. *East Mediterr Health J* 2018;24:42–51.

45 Kim Y. Economic sanctions and HIV/AIDS in women. *J Public Health Policy* 2019;40:351–66.

46 Kim Y. Economic sanctions and child HIV. *Int J Health Plann Manage* 2019;34:693–700.

47 McLean E, Whang T. Economic sanctions and government spending adjustments: the case of disaster preparedness. *British Journal of Political Science* 2019;51:394–411.

48 Mladenovic D, Langeggen I. The impact of war and economic sanction on the incidence of retinopathy of prematurity in Serbia. *Journal of Visual Impairment & Blindness* 2009;103:162–72.

49 Mulder-Sibanda M. Nutritional status of Haitian children, 1978-1995: deleterious consequences of political instability and international sanctions. *Revista Panamericana de Salud Pública* 1998;4:346–49.

50 Parker DP, Foltz JD, Elsea D. Unintended consequences of aanctions for human rights: conflict minerals and infant mortality. *Journal of Law and Economics* 2016;59:731–74.

51 Reid BC, Psoter WJ, Gebrian B, et al. The effect of an international embargo on malnutrition and childhood mortality in rural Haiti. *Int J Health Serv* 2007;37:501–13.

52 Sharma A, Mishra SR, Kaplan WA. Trade in medicines and the public's health: a time series analysis of import disruptions during the 2015 India-Nepal border blockade. *Glob Health* 2017;13:61.

53 Asadi-Pooya AA, Tavana B, Tavana B, et al. Drug adherence of patients with epilepsy in Iran: the effects of the international economic sanctions. *Acta Neurol Belg* 2016;116:151–55.

54 Joury E, Al-Kaabi R, Tappuni AR. Constructing public health policies in post crisis countries: lessons to learn from the associations between free-sugars consumption and diabetes, obesity and dental caries before, during and after sanctions in Iraq. *Journal of Public Health (Zeitschrift für Gesundheitswissenschaften)* 2016;24:563–69.

55 Berggren G, Castle S, Chen L, et al. Sanctions in Haiti: crisis in humanitarian action. Cambridge, MA: Havard School of Public Health 1993.

56 Peksen D. Economic sanctions and human security: the public health effect of economic sanctions. *Foreign Policy Analysis* 2011;7:237–51.

57 Petrescu IM. The humanitarian impact of economic sanctions. *Europolity* 2016;10:205–46.

58 Ali MM, Blacker J, Jones G. Annual mortality rates and excess deaths of children under five in Iraq, 1991-98. *Popul Stud (Camb)* 2003;75:217–26.

59 Abbara A, Rawson TM, Karah N, et al. Antimicrobial resistance in the context of the syrian conflict: drivers before and after the onset of conflict and key recommendations. *Int J Infect Dis* 2018;73:1–6.

60 Abbas WA, Azar NG, Haddad LG, et al. Preconception health status of iraqi women after trade embargo. *Public Health Nurs* 2008;25:295–303.

61 Abdoli A. Iran, sanctions, and the COVID-19 crisis. *J Med Econ* 2020;23:1461–65.

62 Afshari R, Bhopal RS. Iran, sanctions, and collaborations. *Lancet* 2016;387:1055–56.

63 Ahmad K. UN Sanctions imposed against afghanistan while thousands flee. *Lancet* 2001;357:207.

64 Ahmadi AM, Meskarpour-Amiri M. The public health effects of economic sanctions as a global concern in 21th century: why the economic sanctions is a cruel strategy. *Journal of Health Policy and Sustainable Health* 2015;2:145–46.

65 Ahmed NAM, Åstrøm AN, Skaug N, et al. Dental caries prevalence and risk factors among 12-year old schoolchildren from Baghdad, Iraq: A post-war survey, *Int Dent J* 2007;57:36–44.

66 Akbarialiabad H, Rastegar A, Bastani B. How sanctions have impacted iranian healthcare sector: a brief review. *Arch Iran Med* 2021;24:58–63.

67 Akbarpour Roshan N, Abbasi M. The impact of the us economic sanctions on health in Cuba, *Open Access Journal of Resistive Econmics* 2014;2:20–38.

68 Akunjee M, Ali A. Healthcare under sanctions In Iraq: an elective experience. *Med Confl Surv* 2002;18:249–57.

69 Al Faisal W, Sen K, Al Saleh Y. Syria: public health achivements and the effect of sanctions. *Indian J Med Ethics* 2012;9:151–53.

70 Al Faisal W, Al Saleh Y, Sen K. Syria: public health achievements and sanctions, *Lancet* 2012;379:2241.

71 Al Faisal W, Sen K. Syria: effects of conflict and sanctions on public health: response to Coutts' correspondence. *J Public Health (Oxf)* 2013;35:481.

72 Al Samaraie NA. Humanitarian implications of the wars in Iraq. *International Review of the Red Cross* 2007;89:929–42.

73 Albright MK. Economic sanctions and public health: a view from the department of state, *Ann Inter Med* 2000;132:155–57.

74 Al-Nouri L, Al-Rahim Q. The effect of sanctions on children of Iraq. *Arch Dis Child* 2003;88:92.

75 Aloosh M, Aloosh A. Lift sanctions now to save public health. *Nature* 2015;520:623.

76 Aloosh M, Salvati A, Aloosh A. Economic sanctions threaten population health: the case of Iran. *Public Health* 2019;169:10–13.

77 Ameri A, Barzegartahamtan M, Ghavamnasiri M, et al. Current and future challenges of radiation oncology in Iran: a report from the Iranian Society of Clinical Oncology. *Clinical Oncology (R Coll Radiol)* 2018;30:262–68.

78 Andrews TC, Cull DL, Pelton JJ, et al. Self-mutilation and malingering among Cuban migrants detained at Guantanamo Bay, *N Engl J Med* 1997;336:1251–53.

79 Appleyard WJ. WMA wants medicines and foods to be excluded from economic sanctions, *BMJ* 1998;316:76.

80 Arab-Zozani M, Ghoddoosi-Nejad D. COVID-19 in Iran: the good, the bad, and the ugly strategies for preparedness, *Disaster Med Public Health Prep* 2021;15:e43–e45.

81 Aziz C. Struggling to rebuild Iraq's health-care system. *Lancet* 2003;362:1288–89.

82 Baradaran-Seyed Z, Majdzadeh R. Economic sanctions strangle Iranian's health, not just drug supply. *Lancet* 2013;381:1626.

83 Baram A. The effect of Iraqi sanctions: statistical pitfalls and responsibility. *Middle East Journal* 2000;54:194–23.

84 Barnouti HN. Effect of sanctions on surgical practice. *BMJ* 1996;313:1474–75.

85 Barry M. Effect of the US embargo and economic decline on health in Cuba. *Ann Inter Med* 2000;132:151–54.

86 Barry M. The economic embargo against Haiti increased morbidity and mortality of Haitians. *Evidence-based Healthcare* 2000;4:53.

87 Bastani P, Dehghan Z, Kashfi SM, et al. Strategies to improve pharmaceutical supply chain resilience under politico-economic sanctions. The case of Iran. *Journal of Pharmaceutical Policy and Practice* 2021;14:56.

88 Bastani P, Hakimzadeh SM, Teymourzadeh E, et al. Universal health coverage under the Joint Comprehensive Plan of Action's sanctions. *Health Promot Int* 2021;36:693–702.

89 Batmanghelidj E, Heydari G. Sanctions, smuggling, and the cigarette: the granting of Iran Office of Foreign Asset Control's licences to big tobacco. *International Journal of Preventive Medicine* 2014;5:138–44.

90 Benjamin ER, Clements C, McCally M, et al. The humanitarian cost of a war in Iraq. *Lancet* 2003;361:874.

91 Bessler M, Garfield R, Mc Hugh G. Sanction assessment handbook. Assessing the humanitarian implications of sanctions. New York, NY: United Nations–Office for the Coordination of Humanitarian Affairs 2004.

92 Bessler M, Garfield R, Mc Hugh G. Field guidelines for assessing the humanitarian implications of sanctions. New York, NY: United Nations–Office for the Coordination of Humanitarian Affairs 2004.

93 Black M. Collapsing health care in Serbia and Montenegro. *BMJ* 1993;307:1135–37.

94 Centers for Disease Control. Epidemic neuropathy, Cuba 1991–1994. *JAMA* 1994;271:1154–56.

95 Chelala C. Fighting for survival. *BMJ* 1994;309:525–26.

96 Chelala C. Relations between the United States and Cuba. A proposal for action. *JAMA* 1996;275:559–60.

97 Chelala C. Cuba shows health gains despite embargo. *BMJ* 1998;316:497.

98 Cheraghali AM. Impacts of international sanctions on Iranian pharmaceutical market, *Daru* 2013;21:64.

99 Choonara I. Economic sanctions and child health. *Med Confl Surv* 2013;29:93–98.

100 Cohen R. Sanctions hurt but are not the main impediment to humanitarian operations in North Korea. *Asia Policy* 2018;13:35–41.

101 Coovadia HM. Sanctions and the struggle for health in South Africa. *Am J Public Health* 1999;89:1505–08.

102 Cotton P. Cause of Cuba outbreak neuropathologic puzzle. *JAMA* 1993;270:421–23.

103 Cuellar NG. Cuban embargo restrictions lifted. Impact on health care?. *J Transcult Nurs* 2015;26:217–18.

104 Danaei G, Harirchi I, Sajadi HS, et al. The harsh effects of sanctions on Iranian health. *Lancet* 2019;394:468–69.

105 De Vos P, García Fariña A, Álvarez-Pérez A, et al. Public health services, an essential determinants of health during crisis. Lessons from Cuba, 1989–2000, *Trop Med Int Health* 2012;17:469–79.

106 Dehghani M, Mesgarpour B, Akhondzadeh S, et al. How the US sanctions are affecting the health research system in Iran? *Arch Iran Med* 2021;24:101–06.

107 Delamothe T. Embargoes that endager health: doctors should oppose them. *BMJ* 1997;315:1393–94.

108 Destafkan R, Salehi H, Hooshmand MM. Provision of peace and right to health through sanctions: threats and opportunities, *Arch Iran Med* 2020;23:S43–S48.

109 Dobson R. Sanctions against Iraq 'double' child mortality. *BMJ* 2000;321:1490.

110 Drain PK. Implications of repealing the Cuban embargo for US medicine and public health. *Am J Public Health* 2015;105:2210–11.

111 Drain PK, Barry M. Fifty years of US embargo. Cuba's health outcomes and lessons. *Science* 2010;328:572–73.

112 Eastman-Abaya R. Life after Sanctions: the Fate of Iraq. *Lancet* 2000;356:685.

113 Eisenberg L. The sleep of reason produces monsters – human costs of economic sanctions. *N Engl J Med* 1997;336:1248–50.

114 Fakheran O. Economic sanctions and dental public health in Iran. *Journal of Oral Health and Oral Epidemiology* 2019;8:52–54.

115 Farmer P, Fawzi MCS, Nevil P. Unjust embargo of aid for Haiti. *Lancet* 2003;361:420–23.

116 Farsad M, Rahmim A, Dadpavar S, et al. Economic sanctions are against basic human rights on health, *Eur J Nucl Med Mol Imaging* 2019;46:1046–47.

117 Field JO, Russell RM. Nutrition mission to Iraq for UNICEF. *Nutr Rev* 1992;50:41–46.

118 Frankish H. Health of the Iraqi people hangs in the balance. *Lancet* 2003;361:623–25.

119 Garfield R. The impact of economic sanctions on health and well-being. London: Overseas Development Institute, 1999.

120 Garfield R. Suffer the innocents, *The Sciences* 1999;39:19–23.

121 Garfield R. Economic sanctions on Yugoslavia. *Lancet* 2001;358:580.

122 Garfield R. Economic sanctions, humanitarianism, and conflict after the Cold War. *Social Justice* 2002;29:94–107.

123 Garfield R, Devin J, Fausey J. The health impact of economic sanctions. *Bulletin of the New York Academy of Medicine* 1995;72:454–69.

124 Garfield R, Zaidi S, Lennock J. Medical care in Iraq after six years of Sanctions, *BMJ* 1997;315:1474–75.

125 Ghalibafian M, Hemmati S, Bouffet E. The silent victims of the US embargo against Iran. *Lancet Oncol* 2018;19:e580.

126 Gharebaghi R, Heidary F. COVID-19 and Iran: Swimming with hands tied! *Swiss Med Wkly* 2020;150:w20242.

127 Gibbons E, Garfield R. The impact of economic sanctions on health and human rights in Haiti, 1991–1994. *Am J Public Health* 1999;89:1499–04.

128 Gorji A. Medical supplies in Iran hit by sanctions. *Nature* 2013;495:314.

129 Gorji A. Sanctions against Iran: the impact on health services. *Iranian Journal of Public Health* 2014;43:381–82.

130 Habibzadeh P. Sanctions on health education. *Arch Iran Med* 2016;19:610.

131 Heidari R, Akbariqomi M, Tavoosidana G. Medical legacy of sanctions in Iran. *Nature* 2017;552:175.

132 Hosseini SA. Impact of sanctions on procurement of medicine and medical devices in Iran: a technical response. *Arch Iran Med* 2013;16:736–38.

133 Huertas E, Martin GE, Kirkpatrick AF, et al. Correspondence on 'Medicine and the US embargo against Cuba'. *JAMA* 1996;275:1633–34.

134 Kandela P. Effects of sanctions on Iraq's health professionals. *Lancet* 1997;349:1153.

135 Kandela P. Iraq measures the health effects of sanctions. *Lancet* 1997;349:1896.

136 Karimi A, Turkamani HS. US-imposed economic sanctions on Iran in the COVID-19 crisis from the human rights perspective. *Int J Health Ser* 2021;51:570–72.

137 Keck CW, Reed GA. The curious case of Cuba. *Am J Public Health* 2012;102:e13–e22.

138 Khanal V, Mishra SR, DeYoung SE. Nepal's crisies threaten gains in public health. *Am J Public Health* 2016;106:e29–e30.

139 Kheirandish M, Rashidian A, Bigdeli M. A news media analysis of economic sanction effects on access to medicine in Iran. *Journal of Research in Pharmacy Practice* 2015;4:199–205.

140 Kheirandish M, Rashidian A, Kebraeezade A, et al. A review of pharmaceutical policies in response to economic crises and sanctions. *Journal of Research in Pharmacy Practice* 2015;4:115–22.

141 Kirkpatrick AF. Role of the USA in shortage of food and medicine in Cuba. *Lancet* 1996;348:1489–91.

142 Kirkpatrick AF. The US attack on Cuba's health. *CMAJ* 1997;157:281–84.

143 Kokabisaghi F. Assessment of the effects of economic sanctions on Iranians' right to health by using human rights impact assessment tool: a systematic review. *Int J Health Policy Manag* 2018;7:374–93.

144 Kokabisaghi F, Miller AC, Bashar F, et al. Impact of United States political sanctions on international collaborations and research in Iran. *BMJ Glob Health* 2019;4:e001692.

145 Kumar S. India to raise health spending to counteract foreign aid sanctions. *Lancet* 1998;351:1794.

146 Kumar S. India's health saved from sanctions. *Lancet* 1998;352:125.

147 Kuntz D. The politics of suffering: the impact of the US embargo on the health of the Cuban people. *Int J Health Serv* 1994;24:161–79.

148 Lafta RK, Al-Nuaimi MA. War or health: a four-decade armed conflict in Iraq. *Med Confl Surv,* 35:209–26.

149 Larijani B. Burden of diabetes in Iran: how will it be affected by lifting the economic sanctions? *Lancet Diabetes Endocrinol* 2016;10:810–11.

150 Lee I, Haines A. Health costs of the Gulf war. *BMJ* 1991;303:303–06.

151 Legetic B, Jakovljevic D, Marinkovic J, et al. Health care delivery and the status of the population's health in the current crises in former Yugoslavia using EPI-design methodology, *Int J Epidemiol* 1996;25:341–48.

152 Lyme RF. Sanctioning Assad's Syria: mapping the economic, socioeconomic and political repercussions of the international sanctions imposed on Syria since March 2011. Copenaghen: Danish Institute for International Studies 2012.

153 Madani-Lavassani Y. Sanctions on Iran and their impact on child health. *Med Confl Surv* 2020;36:359–67.

154 Marks SP. Economic sanctions as human rights violations: reconciling political and public health imperatives. *Am J Public Health* 1999;89:1509–13.

155 Massoumi RL, Koduri S. Adverse effects of political sanctions on the health care system in Iran. *J Glob Health* 2015;5:020302.

156 Maziak W, Coutts AP, Fouad MF. Beyond sanctions: a response to Sen, et al. *J Public Health (Oxf)* 2013;35:343–44.

157 Maziak W, Coutts AP, Fouad MF. Looking away does not make things vanish. *Indian J Med Ethics* 2013;10:139.

158 McCarthy M. US doctors' group highlights public-health effects of sanctions. *Lancet* 2000;355:296.

159 Mehtarpour M, Takian A, Eshrati B, et al. Control of antimicrobial resistance in Iran: the role of international factors, *BMC Public Health* 2020;20:873.

160 Mohammadi D. US-led economic sanctions strangle Iran's drug supply. *Lancet* 2013;381:279.

161 Moret E. Humanitarian impacts of economic sanctions on Iran and Syria. *European Security,* 2014.

162 Morin K, Miles SH. The health effects of economic sanctions and embargoes: the role of health professionals. *Ann Inter Med* 2000;132:158–61.

163 Namazi S. Sanctions and medical supply shortages in Iran. Washington, DC: Woodrow Wilson International Center for Scholars 2013.

164 Nasheit NA. Perinatal and neonatal mortality and morbidity in Iraq. *J Matern Fetal and Neonatal Med* 2003;13:64–67.

165 Ogbonna CC. Targeted or restrictive: impact of US and EU sanctions on education and healthcare of Zimbabweans. *African Research Review* 2017;11:31–41.

166 Parnham-Cope D. Doctors have moral imperative to call for end of embargo on Cuba. *BMJ* 1997;315:1463.

167 Peyravi M, Ahmadi Marzaleh M. The effect of the US sanctions on humanitarian aids during the great flood of Iran in 2019. *Prehosp Disaster Med* 2020;35:233–34.

168 Popal GR. Impact of sanctions on the population of Iraq. *East Mediterr Health J* 2000;6:791–95.

169 Rezaee-Zavareh MS, Karimi-Sari H, Alavian SM. Iran, sanctions, and research collaborations. *Lancet* 2016;387:28–29.

170 Román GC. Epidemic neuropathy in Cuba: a plea to end the United States economic embargo on a humanitarian basis. *J Public Health Policy* 1995;16:5–12.

171 Román GC. On politics and health: an epidemic of neurologic disease in Cuba. *Ann Inter Med* 1995;122:530–33.

172 Román GC. Epidemic neuropathy in Cuba: a public health problem related to the Cuban Democracy Act of the United States. *Neuroepidemiology* 1998;17:111–15.

173 Sahraian MA, Moghadasi AN, Eskandarieh S. Economic sanctions against Iran as an important factor in threatening the health of patients with multiple sclerosis. *Current Journal of Neurology* 2021;20:15–22.

174 Sansom C. The ghost of Saddam and UN sanctions. *Lancet Oncol* 2004;5:143–45.

175 Sato N, Obeid O, Brun T. Malnutrition in Southern Iraq. *Lancet* 1991;338:1202.

176 Sen K, Starvation of children in Syria. *Indian J Med Ethics* 2014;11:63.

177 Sen K, Al-Faisal W. AlSaleh Y. Syria: effects of conflict and sanctions on public health. *J Public Health (Oxf)* 2013;35:195–99.

178 Setayesh S, Mackey TK. Addressing the impact of economic sanctions on Iranian drug shortages in the Joint Comprehensive Plan of Action: promoting access to medicines and health diplomacy. *Glob Health* 2016;12:31.

179 Shahabi S. Sanctions in Iran disrupt cancer care. *Nature* 2015;520:157.

180 Shahabi S, Fazlalizadeh H, Stedman J, et al. The impact of international economic sanctions on Iranian cancer healthcare. *Health Policy* 2015;119:1309–18.

181 Shahabi S, Ahmadi Teymourlouy A, Shabaninehad H, et al. Physical rehabilitation in Iran after international sanctions: explored findings from a qualitative study. *Glob Health* 2020;16:86.

182 Sidel VW. Can sanctions be sanctioned? *Am J Public Health* 1999;89:1497–98.

183 Smith MC, Zaidi S. Malnutrition in Iraqi children following the Gulf war: results of a national survey. *Nutr Rev* 1993;51:74–78.

184 Sponeck HCG. Sanctions and humanitarian exemptions: a practitioner's comentary. *European Journal of International Law* 2002;13:81–87.

185 Stix G. Ban that embargo. *Sci Am* 1995;272:32–34.

186 Takian A, Raoofi A, Kazempour-Ardebili S. COVID-19 battle during the toughtest sanctions against Iran. *Lancet* 2020;395:1035–36.

187 The Cuba Neuropathy Field Investigation Team. Epidemic optic neuropathy in Cuba: clinical characterization and risk factors, *N Eng J Med* 1995;333:1176–82.

188 The Lancet. Health effects of sanctions in Iraq. *Lancet* 1995;346:1439.

189 The Lancet. Sanctions on Health in Cuba. *Lancet* 1996;348:1461.

190 Velayati AA, Jaamati H, Hashemian SM. Interim initiative for health in Iran. *Nature* 2015;521:32.

191 Wakai S. Life after sanctions: the fate of Iraq. *Lancet* 2000;356:685.

192 Wareham SJ. Economic sanctions and public health: the case of Iraq. *Med J Aust* 2000;173:438–39.

193 Weeramanthri TS, Gruen RL, Yee TF, et al. Economic sanctions and public health: the case of Cuba. *Med J Aust* 2001;174:316.

194 World Health Organization. Technical discussion. Health under difficult circumstances: the impact of war, disasters and sanctions on the health of populations. Cairo: Regional Office for the Eastern Mediterranean, 2002.

195 Yamada S, Fawzi MCS, Maskarinec GC, et al. Casualties: narrative and images of the war in Iraq. *Int J Health Serv* 2006;36:401–15.

196 Yazdi-Feyzabadi V, Amini-Rarani M, Delavari S. The health consequences of economic sanctions: call for health diplomacy and international collaboration. *Arch Iran Med* 2020;23:s51–s53.

197 Yoon HJ, Woo SH, Kim D, et al. Changes in medical research trends of North Korea after economic sanctions: a PRISMA-compliant systematic literature review of North Korean medical journals. *Medicine* 2019;98:e16500.

198 Younis MS, Aswad AM. The impact of war and economic sanctions on the mental health system in Iraq from 1990 to 2003. A preliminary report. *Intervention* 2018;16:54–58.

199 Yousefi N, Moradi N, Dinarvand R, et al. Policies to improve access to pharmaceutical products in shortage: the experience of Iran Food and Drug Administration. *Daru* 2019;27:169–77.

200 Zadeh-Cummings N, Harris L. The impact of sanctions against North Korea on humanitarian aid. *Journal of Humanitarian Affairs* 2020;2:44–52.

201 Zaidi S. War, sanctions, and humanitarian assistance: the case of Iraq 1990–1993. *Med Glob Surv* 1994;1:147–55.

202 Zakavi SR. Economic sanctions on Iran and nuclear medicine. *Asia Oceania Journal of Nuclear Medicine & Biology* 2019;7:1–3.

203 Zartab S, Nassiri Koopaei N, Abbasian H, et al. The impact of sanction and healthcare system reform on the healthcre performance and pharmaceutical market in Iran, 2001–2016. *Journal of Pharmaceutical Policy and Practice* 2020;13:50.

204 Zolotarev O, Tayebi SK, Khakimova A, et al. Analysis of the impact of economic sanctions on health research and publication activities of scientists from Iran. *International Economic Studies* 2020;50:47–60.

205 Morton B, Vercueil A, Masekela R, et al. Consensus statement on measures to promote equitable authorship in the publication of research from international partnerships. *Anaesthesia* 2022;77:264–76.
